# Design, Synthesis, and Evaluation of Anticonvulsant Activities of New Triazolopyrimidine Derivatives

**DOI:** 10.3389/fchem.2022.925281

**Published:** 2022-06-23

**Authors:** Mingxia Song, Wennan Zhao, Yangnv Zhu, Wenli Liu, Xianqing Deng, Yushan Huang

**Affiliations:** ^1^ Medical College, Jinggangshan University, Jiʼan, China; ^2^ Jiʼan Key Laboratory of Personalized Drug Research of Neuropsychiatric Diseases, Jiʼan, China; ^3^ Center for Evidence Based Medical and Clinical Research, First Affiliated Hospital of Gannan Medical University, Ganzhou, China

**Keywords:** epilepsy, triazole, anticonvulsant, GABA, benzodiazepine (BZD) receptors, docking

## Abstract

Epilepsy, a severe brain disease affecting a large population, is treated mainly by antiepileptic drugs (AEDs). However, toxicity, intolerance, and low efficiency of the available AEDs have prompted the continual attempts in the discovery of new AEDs. In this study, we discovered a skeleton of triazolopyrimidine for the development of new AEDs. The design, synthesis, *in vivo* anticonvulsant activity evaluation of triazolopyrimidines **(3a–3i** and **6a–6e)**, and pyrazolopyrimidines **(4a–4i)** are reported. We found that most triazolopyrimidines showed anticonvulsive activity in the maximal electroshock (MES) and pentetrazol (PTZ)-induced seizure models. On the contrary, pyrazolopyrimidines **(4a–4i)** showed weak or no protective effects. Among the tested derivatives, compound **6d**, holding a median effective dose (ED_50)_ of 15.8 and 14.1 mg/kg against MES and PTZ-induced seizures, respectively, was found to be the most potent one. Moreover, the protection index (PI) value of **6d** was significantly higher than that of the available AEDs such as valproate, carbamazepine, and diazepam. The antiepileptic efficacy of compound **6d** was also observed in the 3-mercaptopropionic acid and bicuculline-induced seizure models. Antagonistic effects of flumazenil and 3-MP for the anticonvulsive activity of **6d** and also the radioligand-binding assay confirmed the involvement of GABA receptors, at least benzodiazepine (BZD) receptor, in the anticonvulsant activity of compound **6d**. The docking study of compounds **4e** and **6d** with GABA_A_ receptor confirmed and explained their affinity to the BZD receptors.

## 1 Introduction

Epilepsy is a kind of common brain disease accompanied by repeated and unprovoked seizures. As one of the most common CNS disorders, it affects individuals of any age, any gender, and any ethnicity. About 3–4% of people in developed countries encountered this disease during their lifetime. For people in developing countries, this proportion is higher (Beghi and Hesdorffer, 2014). Epilepsy has a deleterious impact on society, economy, and specifically the physical and psychological well-being of patients (Perucca et al., 2018). According to the Global Burden of Disease Study (GBD) 2019, idiopathic epilepsy was ranked 20th in respect of disability-adjusted life years (DALYs) among 369 health conditions (GBD 2019 Diseases and Injuries Collaborators, 2020).

**FIGURE 1:**
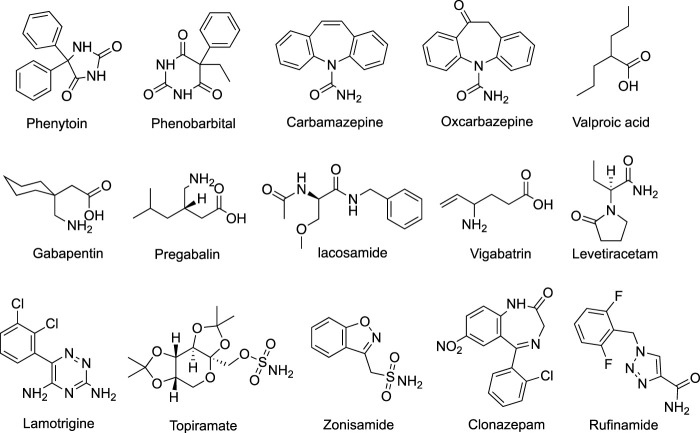
Structures of several common AEDs.

Antiepileptic drugs (AEDs) are the main method of epilepsy treatment. About two-thirds of patients with epilepsy can achieve seizure freedom. Since the 1990s, numbers of second-generation AEDs and third-generation AEDs (developed after 2000) have been introduced (Figure 1), expanding the opportunity for individual therapy for epilepsy patients. Some of them offer advantages in terms of favorable pharmacokinetics, fewer drug interactions, less adverse effects, and improved tolerability (Vidaurre and Herbst, 2019). Nevertheless, they did not considerably change the overall proportion of seizure-protected patients with epilepsy. Therefore, finding novel AEDs with high efficiency and low toxicity is still the focus of current AED therapy.

Triazole refers to a five-membered heterocycle with the molecular formula C_2_H_3_N_3_. It can be considered as an isostere of pyrazole, thiazole, imidazole, and oxazole. The electron richness and aromaticity of triazole enable it to freely bind with a wide range of biomacromolecules by interactions of pi–pi bonds, H bonds, and ion-dipole bonds. Therefore, it has been widely employed in various fields, especially in pharmaceutical research. Many drugs (such as triazolam, alprazolam, rufinamide, fluconazole, itraconazole, lamtidine, ribavirin, brassinazole, and sitagliptin) assembling the triazole moiety have already been used in clinics for the treatment of various diseases. Moreover, a terrific amount of triazole-contained derivatives have been designed and prepared to discover new anticonvulsants (Song and Deng, 2018), anti-Alzheimer (Xu et al., 2019), anticancer (Wen et al., 2020), antibacterial (Gao et al., 2019), antifungal (Peyton et al., 2015), and anti-inflammatory agents (Zhou and Wang, 2012).

Diazepam, a benzodiazepine drug, is often used to treat anxiety disorders, alcohol withdrawal syndrome, benzodiazepine withdrawal syndrome, seizures, and insomnia (Calcaterra and Barrow, 2014). The efficacy of diazepam comes from the activation of γ-aminobutyric acid (GABA) after it binds to benzodiazepine receptors (Riss et al., 2008). It is the top choice for the epileptic status to receive the intravenous or rectal administration of diazepam. Eighty percent of cases with different forms of epileptic status can be suppressed, and the effective proportion increases for generalized seizures (Loiseau, 1983). However, the side effects of diazepam including drowsiness, motor coordination disorders, and drug dependence limit its application in the clinics (Wu et al., 2016).

**FIGURE 2:**
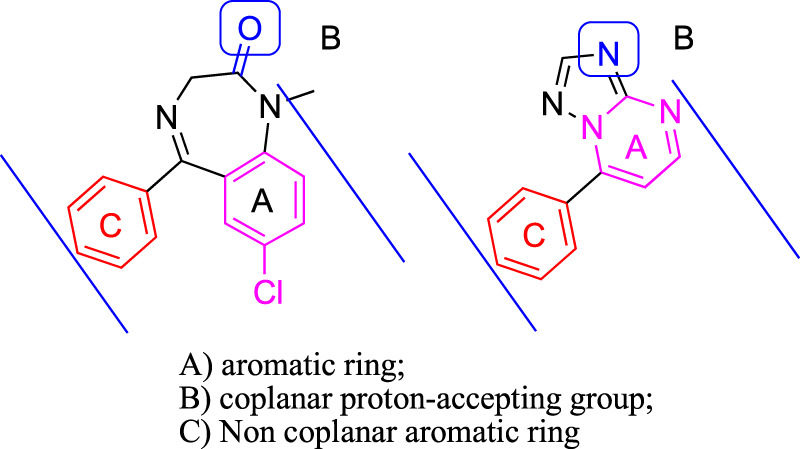
Pharmacodynamic characteristics of benzodiazepine site agonists (diazepam as a representative) and compounds reported herein (**3a** as a representative).

Based on these facts, a series of 7-substituted-[1,2,4]triazolo[1,5-α]pyrimidine derivatives (**3a–3i**) were designed and synthesized to develop new anticonvulsant agents in this study. The rationale behind this design refers to the similarity between the target compounds **3a–3i** and diazepam (Figure 2). The target compounds have the requirements for binding to benzodiazepine (BZD) receptors 1) an aromatic ring A, 2) a coplanar proton-accepting group B, and 3) a non-coplanar aromatic ring C. With an intention, the designed compounds act as diazepam to display anticonvulsant activity through binding to BZD receptors. In addition, the triazole moiety in the compounds **3a–3i** was replaced by pyrazole to give bioisosteric **4a–4i**. These bioisosteres, without the proton-accepting group in a suitable position, were designed to enrich the structure–activity relationships (SARs) and verify the rationality of the aforementioned design. To obtain more SARs around the triazole derivatives, some triazole derivatives (**6a–6e**) with higher hydrophobicity were also prepared.

## 2 Materials and Methods

### 2.1 Chemical Part

#### 2.1.1 Instruments and Reagents

Melting points were determined in open capillary tubes and were uncorrected. ^1^H NMR and ^13^C NMR spectra were measured using an AV-300 spectrometer (Bruker, Switzerland). High-resolution mass spectra were recorded using a MALDI-TOF/TOF mass spectrometer (Bruker Daltonik, Germany). The majority of chemicals were purchased from Aldrich Chemical Corporation (St Louis, United States).

#### 2.1.2 Synthesis of (*E*)-3-(Dimethylamino)-1-Phenylprop-2-en-1-Ones (**2a–2i**)

Considering compound **2a** as an example: acetophenone (1.00 g, 8 mmol) and DMF-DMA (1.50 g, 13 mmol) were mixed and stirred at 100°C for about 10 h. The reaction was monitored by TLC (30% ethyl acetate in petroleum ether). After the reaction was completed and cooled to about 40°C, 10 ml of petroleum ether was added and stirred until it was completely dissolved. The mixture was placed in a fridge to maintain at 4°C overnight. The solid precipitated was filtered and dried to obtain compound **2a**. Mp 92°C–94°C, yield 77.4%. ^1^H-NMR (CDCl_3_, 400 MHz): *δ* 2.92 (s, 3H, NCH_3_), 3.13 (s, 3H, NCH_3_), 5.73 (d, 1H, *J* = 12.3, =CH), 7.81 (d, 1H, *J* = 12.3 Hz, =CH), 7.39–7.48 (m 3H, Ph-H), and 7.89–7.92 (m 2H, Ph-H). ^13^C-NMR (CDCl_3_, 101 MHz): *δ* 188.7, 154.3, 140.5, 130.9, 128.1, 127.5, 92.2, 45.1, and 37.3. ESI-HRMS calculated for C_11_H_14_NO^+^ ([M + H]^+^): 176.1070; found: 176.1074. Compounds **2b–2i** were prepared from other substituted acetophenones under the same conditions.

#### 2.1.3 Synthesis of 7-Substituted-[1,2,4]Triazolo[1,5-a]Pyrimidines (**3a-3i**)

Considering compound **3a** as an example: compound **2a** (0.7 g, 4 mmol), 3-amino-1,2,4-triazole (0.34 g, 4 mmol), and 4 ml of glacial acetic acid were mixed and stirred at 120°C for about 7 h. The reaction was monitored by TLC (50% ethyl acetate in petroleum ether). After the reaction was completed, 40 ml of ice water was added to precipitate, which was filtered and recrystallized with 50% ethanol to obtain compound **3a**. Compounds **3b–3i** were prepared under the same conditions.

#### 2.1.4 Synthesis of 7-Phenylpyrazolo[1,5-a]Pyrimidines (**4a-4i**)

Considering compound **4a** as an example: compound **2a** (0.7 g, 4 mmol), 3-aminopyrazole (0.33 g, 4 mmol), and 4 ml of glacial acetic acid were mixed and stirred at 120°C for about 7 h. TLC (50% ethyl acetate in petroleum ether) was used to monitor the reaction process. After the reaction was completed, 40 ml of ice water was added to precipitate, which was filtered and recrystallized with 50% ethanol to obtain compound **4a**. Compounds **4b–4i** were prepared under the same conditions.

#### 2.1.5 Synthesis of 4-([1,2,4]Triazolo[1,5-a]Pyrimidin-7-yl)Phenol (5)

Compound **3i** (1.13 g, 5 mmol), BBr_3_ (5.00 g, 20 mmol), and 30 ml of dry CH_2_Cl_2_ were mixed and stirred in an ice bath for about 6 h. TLC (66% ethyl acetate in petroleum ether) was used to monitor the reaction process. After it was completed, the reaction was quenched by ice water. The organic solvent evaporated under reduced pressure to leave the product dispersed in the water, which was filtered to give compound **5**. Mp 230–232°C, yield 89%. ^1^H-NMR (DMSO-*d*_*6*_, 400 MHz): *δ* 7.4 (d, 2H, *J* = 8.6 Hz, Ph-H), 7.61 (d, 1H, *J* = 4.8 Hz, Py-H), 8.19 (d, 2H, *J* = 8.6 Hz, Ph-H), 8.80 (s, 1H, Triazole-H), and 8.89 (d, 1H, *J* = 4.8 Hz, Py-H). ^13^C NMR (101 MHz, DMSO-*d*_*6*_): *δ* 161.6, 155.8, 155.5, 155.0, 148.0, 132.1, 120.0, 116.1, and 109.0. ESI-HRMS calculated for C_11_H_9_N_4_O^+^ ([M + H]^+^): 213.0771; found: 213.0777.

#### 2.1.6 Synthesis of 7-(4-Alkoxyphenyl)-[1,2,4]Triazolo[1,5-a]Pyrimidines (**6a-6e**)

Considering compound **6a** as an example: compound **5** (0.42 g, 2 mmol), K_2_CO_3_ (0.28 g, 2 mmol), bromopropane (0.30 g, 2.4 mmol), and 30 ml of acetonitrile were mixed and stirred at 90°C for about 8 h. TLC (50% ethyl acetate in petroleum ether) was used to monitor the reaction process. After the reaction was completed, the organic solvent was evaporated, and the residue was added to 30 ml of water and extracted by dichloromethane (3 ml × 30 ml). The combined dichloromethane was dried by MgSO_4_ and evaporated in a vacuum to give a residue, which was purified by column chromatography (silica gel, petroleum ether/EtOAc, 70:30) to provide compound **6a**. Compounds **6b –6e** were prepared under the same conditions using other haloalkanes.

The physical data of target compounds (**3a**–**3i**, **4a**–**4i**, and **6a**–**6e**) are listed as follows:

7-Phenyl-[1,2,4]triazolo[1,5-a]pyrimidine (**3a**):

Mp 142–143°C, yield 65.1%. ^1^H-NMR (DMSO-*d*_6_, 300 MHz): *δ* 7.61–8.21 (m, 6H, Ph-H, pyrimidine-H), 8.73 (s, 1H, triazole-H), and 8.96 (d, 1H, *J* = 4.5 Hz, pyrimidine-H). ^13^C-NMR (DMSO-*d*_6_, 75 MHz): *δ* 156.23, 156.00, 155.53, 147.78, 132.17, 130.07, 130.01, 129.15, and 110.16. ESI-HRMS calculated for C_11_H_9_N_4_^+^ ([M + H]^+^): 197.0822; found: 197.0826.

7-(4-Fluorophenyl)-[1,2,4]triazolo[1,5-a]pyrimidine (**3b**):

Mp 241–244°C, yield 74.7%. ^1^H-NMR (CDCl_3_, 300 MHz): *δ* 7.24 (d, 1H, *J* = 4.2 Hz, pyrimidine-H), 7.28–8.20 (m, 4H, Ph-H), 8.57 (s, 1H, triazole-H), and 8.88 (d, 1H, *J* = 4.2 Hz, pyrimidine-H). ^13^

C-NMR (CDCl_3_, 75 MHz): *δ* 164.78 (d, ^1^ *J*_c-f_ = 251.3 Hz), 156.32, 155.89 (d, ^4^ *J*_c-f_ = 3.6 Hz), 154.47, 147.28, 131.77 (d, ^3^ *J*_c-f_ = 8.7 Hz), 125.73, 116.33 (d, ^2^ *J*_c-f_ = 21.8 Hz), and 108.91. ESI-HRMS calculated for C_11_H_8_FN_4_^+^ ([M + H]^+^): 215.0728; found: 215.0730.

7-(2-Chlorophenyl)-[1,2,4]triazolo[1,5-a]pyrimidine (**3c**):

Mp 148–149°C, yield 58.4%. ^1^H-NMR (CDCl_3_, 300 MHz): *δ* 7.21–7.65 (m, 5H, Ph-H, pyrimidine-H), 8.54 (s, 1H, triazole-H), and 8.95 (d, 1H, *J* = 3.7 Hz, pyrimidine-H). ^13^C-NMR (CDCl_3_, 75 MHz): *δ* 155.89, 155.59, 154.36, 146.28, 133.38, 132.45, 131.01, 130.50, 129.08, 127.25, and 111.62. ESI-HRMS calculated for C_11_H_8_ClN_4_^+^ ([M + H]^+^): 231.0432; found: 231.0437.

7-(4-Chlorophenyl)-[1,2,4]triazolo[1,5-a]pyrimidine (**3d**):

Mp 232–233°C, yield 79.2%. ^1^H-NMR (CDCl_3_, 300 MHz): *δ* 7.26 (d, 1H, *J* = 3.9 Hz, pyrimidine-H), 7.60 (d, 2H, *J* = 8.4 Hz, Ph-H), 8.11 (d, 2H, *J* = 8.4 Hz, Ph-H), 8.57 (s, 1H, triazole-H), and 8.88 (d, 1H, *J* = 3.9 Hz, pyrimidine-H). ^13^C-NMR (CDCl_3_, 75 MHz): *δ* 156.17, 155.79, 154.52, 147.17, 138.42, 130.69, 129.35, 127.93, and 109.00. ESI-HRMS calculated for C_11_H_8_ClN_4_^+^ ([M + H]^+^): 231.0432; found: 231.0433.

7-(2,4-Dichlorophenyl)-[1,2,4]triazolo[1,5-a]pyrimidine (**3e**):

Mp 168–169°C, yield 47.4%. ^1^H-NMR (CDCl_3_, 300 MHz): *δ* 7.22 (d, 1H, *J* = 4.1 Hz, pyrimidine-H), 7.50–7.67 (m, 3H, Ph-H), 8.55 (s, 1H, triazole-H), 8.96 (d, 1H, *J* = 4.1 Hz, pyrimidine-H). ^13^C-NMR (CDCl_3_, 75 MHz): *δ* 155.71, 154.43, 145.23, 145.19, 138.27, 134.34, 131.87, 130.54, 127.75, 127.41, and 111.70. ESI-HRMS calculated for C_11_H_7_Cl_2_N_4_^+^ ([M + H]^+^): 265.0042; found: 265.0047.

7-(4-Bromophenyl)-[1,2,4]triazolo[1,5-a]pyrimidine (**3f**):

Mp 226–227°C, yield 50.1%. ^1^H-NMR (CDCl_3_, 300 MHz): *δ* 7.26 (d, 1H, *J* = 4.3 Hz, pyrimidine-H), 7.76 (d, 2H, *J* = 8.4 Hz, Ph-H), 8.03 (d, 2H, *J* = 8.4 Hz, Ph-H), 8.56 (s, 1H, triazole-H), and 8.89 (d, 1H, *J* = 4.3 Hz, pyrimidine-H). ^13^C-NMR (CDCl_3_, 75 MHz): *δ* 156.27, 155.93, 154.45, 147.20, 132.32, 130.80, 128.43, 126.85, and 108.91. ESI-HRMS calculated for C_11_H_8_BrN_4_^+^ ([M + H]^+^): 274.9927; found: 274.9930.

7-(4-Nitrophenyl)-[1,2,4]triazolo[1,5-a]pyrimidine (**3g**):

Mp 248–253°C, yield 65.5%. ^1^H-NMR (CDCl_3_, 300 MHz): *δ* 7.35 (d, 1H, *J* = 3.9 Hz, pyrimidine-H), 8.36 (d, 2H, *J* = 6.6 Hz, Ph-H), 8.49 (d, 2H, *J* = 6.6 Hz, Ph-H), 8.53 (s, 1H, triazole-H), and 8.99 (d, 1H, *J* = 3.9 Hz, pyrimidine-H). ^13^C-NMR (CDCl_3_, 75 MHz): *δ* 156.13, 156.11, 154.63, 149.57, 145.78, 135.35, 130.58, 124.08, and 109.76. ESI-HRMS calculated for C_11_H_8_N_5_O_2_^+^ ([M + H]^+^): 242.0673; found: 242.0676.

7-(P-tolyl)-[1,2,4]triazolo[1,5-a]pyrimidine (**3h**):

Mp 180–181°C, yield 47.6%. ^1^H-NMR (CDCl_3_, 300 MHz): *δ* 2.49 (s, 3H, Ph-CH_3_) 7.24 (d, 1H, *J* = 4.5 Hz, pyrimidine-H), 7.43 (d, 2H, *J* = 7.8 Hz, Ph-H), 8.04 (d, 2H, *J* = 7.8 Hz, Ph-H), 8.57 (s, 1H, triazole-H), and 8.88 (d, 1H, *J* = 4.5 Hz, Pyrimidine-H). ^13^C-NMR (CDCl_3_, 75 MHz): *δ* 156.13, 155.54, 154.49, 148.54, 142.84, 129.69, 129.30, 126.69, 108.84, and 21.64. ESI-HRMS calculated for C_12_H_11_N_4_^+^ ([M + H]^+^): 211.0978; found: 211.0984.

7-(4-Methoxyphenyl)-[1,2,4]triazolo[1,5-a]pyrimidine (**3i**):

Mp 188–189°C, yield 70.0%. ^1^H-NMR (CDCl_3_, 300 MHz): *δ* 3.94 (s, 3H, OCH_3_), 7.12 (d, 2H, *J* = 8.4 Hz, Ph-H), 7.26 (d, 1H, *J* = 4.4 Hz, pyrimidine-H), 8.18 (d, 2H, *J* = 8.4 Hz, Ph-H), 8.58 (s, 1H, triazole-H), and 8.85 (d, 1H, *J* = 4.4 Hz, pyrimidine-H). ^13^C-NMR (CDCl_3_, 75 MHz): *δ* 162.61, 156.21, 155.45, 154.37, 148.14, 131.25, 121.62, 114.43, 108.25, and 55.59. ESI-HRMS calculated for C_12_H_11_N_4_O^+^ ([M + H]^+^): 227.0927; found: 227.0935.

7-Phenylpyrazolo[1,5-a]pyrimidine (**4a**):

Mp 70–71°C, yield 60.0%. ^1^H-NMR (DMSO-*d*_6_, 300 MHz): *δ* 6.84 (d, 1H, *J* = 2.1 Hz, pyrazolo-H), 7.24 (d, 1H, *J* = 4.5 Hz, pyrimidine-H), 7.59–8.15 (m, 5H, Ph-H), 8.27 (d, 1H, *J* = 2.1 Hz, pyrazolo-H), and 8.62 (d, 1H, *J* = 4.5 Hz, pyrimidine-H). ^13^C-NMR (DMSO-*d*_6_, 75 MHz): *δ* 150.03, 149.84, 146.07, 144.94, 131.46, 131.13, 129.85, 128.94, 108.11, and 96.99. ESI-HRMS calculated for C_12_H_10_N_3_^+^ ([M + H]^+^): 196.0869; found: 196.0875.

7-(4-Fluorophenyl)pyrazolo[1,5-a]pyrimidine (**4b**):

Mp 133–134°C, yield 76.4%. ^1^H-NMR (CDCl_3_, 300 MHz): *δ* 6.83 (d, 1H, *J* = 2.1 Hz, pyrazolo-H), 6.91 (d, 1H, *J* = 4.3 Hz, pyrimidine-H), 7.22–8.13 (m, 4H, Ph-H), 8.20 (d, 1H, *J* = 2.1 Hz, pyrazolo-H), and 8.55 (d, 1H, *J* = 4.3 Hz, pyrimidine-H). ^13^C-NMR (CDCl_3_, 75 MHz): *δ* 164.28 (d, ^1^ *J*_c-f_ = 251.1 Hz), 149.55, 148.75, 146.01, 144.87, 131.56 (d, ^3^ *J*_c-f_ = 8.6 Hz), 126.99, 115.96 (d, ^2^ *J*_c-f_ = 21.8 Hz), 107.02, and 97.16. ESI-HRMS calculated for C_12_H_9_FN_3_^+^ ([M + H]^+^): 214.0775; found: 214.0781.

7-(2-Chlorophenyl)pyrazolo[1,5-a]pyrimidine (**4c**):

Mp 113–114°C, yield 58.7%. ^1^H-NMR (CDCl_3_, 300 MHz): *δ* 6.85 (d, 1H, *J* = 2.3 Hz, pyrazolo-H), 6.88 (d, 1H, *J* = 4.1 Hz, pyrimidine-H), 7.47–7.63 (m, 4H, Ph-H), 8.18 (d, 1H, *J* = 2.3 Hz, pyrazolo-H), and 8.60 (d, 1H, *J* = 4.1 Hz, pyrimidine-H). ^13^C-NMR (CDCl_3_, 75 MHz): *δ* 148.85, 148.41, 145.11, 145.01, 133.50, 131.78, 130.94, 130.52, 130.29, 127.12, 108.99, and 97.22. ESI-HRMS calculated for C_12_H_9_ClN_3_^+^ ([M + H]^+^): 230.0480; found: 230.0483.

7-(4-Chlorophenyl)pyrazolo[1,5-a]pyrimidine (**4d**):

Mp 146–147°C, yield 69.1%. ^1^H-NMR (CDCl_3_, 300 MHz): *δ* 6.83 (d, 1H, *J* = 2.3 Hz, pyrazolo-H), 6.91 (d, 1H, *J* = 4.1 Hz, pyrimidine-H), 7.56 (d, 2H, *J* = 8.7 Hz, Ph-H), 8.03 (d, 2H, *J* = 8.7 Hz, Ph-H), 8.18 (d, 1H, *J* = 2.3 Hz, pyrazolo-H), and 8.55 (d, 1H, *J* = 4.1 Hz, pyrimidine-H). ^13^C-NMR (CDCl_3_, 75 MHz): *δ* 149.58, 148.77, 145.83, 144.86, 137.36, 130.62, 129.32, 129.05, 107.07, and 97.25. ESI-HRMS calculated for C_12_H_9_ClN_3_^+^ ([M + H]^+^): 230.0480; found: 230.0483.

7-(2,4-Dichlorophenyl)pyrazolo[1,5-a]pyrimidine (**4e**):

Mp 138–139°C, yield 55.5%. ^1^H-NMR (CDCl_3_, 300 MHz): *δ* 6.84 (d, 1H, *J* = 2.1 Hz, pyrazolo-H), 6.87 (d, 1H, *J* = 3.3 Hz, pyrimidine-H), 7.28–7.64 (m, 3H, Ph-H), 8.60 (d, 1H, *J* = 2.1 Hz, pyrazolo-H), and 8.59 (d, 1H, *J* = 3.3 Hz, pyrimidine-H). ^13^C-NMR (CDCl_3_, 75 MHz): *δ* 149.05, 148.48, 145.07, 143.69, 137.38, 134.46, 131.83, 130.31, 128.96, 127.59, 109.06, and 97.47. ESI-HRMS calculated for C_12_H_8_Cl_2_N_3_^+^ ([M + H]^+^): 264.0090; found: 264.0097.

7-(4-Bromophenyl)pyrazolo[1,5-a]pyrimidine (**4f**):

Mp 172–174°C, yield 50.3%. ^1^H-NMR (CDCl_3_, 300 MHz): *δ* 6.87 (s, 1H, pyrazolo-H), 6.95 (s, 1H, pyrimidine-H), 7.74 (d, 2H, *J* = 7.8 Hz, Ph-H), 7.98 (d, 2H, *J* = 7.8 Hz, Ph-H), 8.21 (s, 1H, pyrazolo-H), and 8.58 (s, 1H, pyrimidine-H). ^13^C-NMR (CDCl_3_, 75 MHz): *δ* 149.37, 148.70, 146.10, 144.96, 132.05, 130.82, 129.73, 125.86, 107.02, and 97.24. ESI-HRMS calculated for C_12_H_9_BrN_3_^+^ ([M + H]^+^): 273.9974; found: 273.9979.

7-(4-Nitrophenyl)pyrazolo[1,5-a]pyrimidine (**4g**):

Mp 252–253°C, yield 51.1%. ^1^H-NMR (DMSO-*d*_6_ + CDCl_3_, 300 MHz): *δ* 7.14 (d, 1H, *J* = 3.8 Hz, pyrimidine-H), 7.67 (s, 1H, pyrazolo-H), 8.22 (s, 1H, pyrazolo-H), 8.37 (d, 2H, *J* = 8.7 Hz, Ph-H), 8.46 (d, 2H, *J* = 8.7 Hz, Ph-H), and 8.66 (d, 1H, *J* = 3.8 Hz, pyrimidine-H). ^13^C-NMR (DMSO-*d*_6_ + CDCl_3_, 75 MHz): *δ* 156.93, 153.82, 149.54, 135.36, 130.65, 128.45, 124.37, 118.29, 112.88, and 102.30. ESI-HRMS calculated for C_12_H_9_N_4_O_2_^+^ ([M + H]^+^): 241.0720; found: 241.0726.

7-(P-tolyl)pyrazolo[1,5-a]pyrimidine (**4h**):

Mp 88–90°C, yield 46.5%. ^1^H-NMR (CDCl_3_, 300 MHz): *δ* 2.47 (s, 3H, Ph-CH_3_), 6.80 (d, 1H, *J* = 2.3 Hz, pyrazolo-H), 6.90 (d, 1H, *J* = 4.3 Hz, pyrimidine-H), 8.39 (d, 2H, *J* = 7.8 Hz, Ph-H), 7.96 (d, 2H, *J* = 7.8 Hz, Ph-H), 8.18 (d, 1H, *J* = 2.3 Hz, pyrazolo-H), and 8.52 (d, 1H, *J* = 4.3 Hz, pyrimidine-H). ^13^C-NMR (CDCl_3_, 75 MHz): *δ* 148.79, 144.72, 141.65, 129.71, 129.41, 129.18, 128.10, 127.19, 106.88, 96.88, and 21.58. ESI-HRMS calculated for C_13_H_12_N_3_^+^ ([M + H]^+^): 210.1026; found: 210.1031.

7-(4-Methoxyphenyl)pyrazolo[1,5-a]pyrimidine (**4i**):

Mp 119–121°C, yield 46.9%.^1^H-NMR (CDCl_3_, 300 MHz): *δ* 3.92 (s, 3H, Ph-OCH_3_), 6.80 (d, 1H, *J* = 2.3 Hz, pyrazolo-H), 6.91 (d, 1H, *J* = 4.4 Hz, pyrimidine-H), 7.10 (d, 2H, *J* = 8.7 Hz, Ph-H), 8.10 (d, 2H, *J* = 8.7 Hz, Ph-H), 8.20 (d, 1H, *J* = 2.3 Hz, pyrazolo-H), and 8.52 (d, 1H, *J* = 4.4 Hz, pyrimidine-H). ^13^C-NMR (CDCl_3_, 75 MHz): *δ* 161.95, 149.45, 148.57, 147.04, 144.76, 131.08, 123.04, 114.17, 106.37, 96.74, and 55.52. ESI-HRMS calculated for C_13_H_12_N_3_O^+^ ([M + H]^+^): 226.0975; found: 226.0980.

7-(4-Propoxyphenyl)-[1,2,4]triazolo[1,5-a]pyrimidine **(6a)**:

Mp 120–121°C, yield 80%. ^1^H-NMR (CDCl_3_, 300 MHz): *δ* 1.08 (t, 3H, *J* = 7.4, CH_3_), 1.84–1.91 (t, 2H, CH_2_), 4.04 (t, 2H, *J* = 6.5 Hz, CH_2_), 7.08–7.11 (dd, 2H, *J*_*1*_ = 6.9 Hz, *J*_*2*_ = 2.1 Hz, Ph-H), 7.21 (d, 1H, *J* = 4.7 Hz, Ph-H), 8.14–8.17 (dd, 2H, *J*_*1*_ = 6.9 Hz, *J*_*2*_ = 2.1 Hz, Ph-H), 8.55 (s, 1H, triazole-H), and 8.82 (d, 1H, *J* = 4.7 Hz, N=C-H). ^13^C NMR (CDCl_3_, 75 MHz): *δ* 162.06, 156.30, 155.56, 154.09, 148.00, 131.07, 121.27, 114.73, 107.95, 69.71, 22.31, and 10.37. ESI-HRMS calculated for C_14_H_15_N_4_O^+^ ([M + H]^+^): 255.1240; found: 255.1228.

7-(4-Butoxyphenyl)-[1,2,4]triazolo[1,5-a]pyrimidine **(6b)**:

Mp 95–97°C, yield 79%. ^1^H-NMR (CDCl_3_, 300 MHz): *δ* 1.00 (t, 3H, *J* = 7.3, CH_3_), 1.49–1.57 (m, 2H, CH_2_), 1.78–1.85 (m, 2H, CH_2_), 4.08 (t, 2H, *J* = 6.5 Hz, CH_2_), 7.08–7.11 (dd, 2H, *J*_*1*_ = 6.9 Hz, *J*_*2*_ = 2.1 Hz, Ph-H), 7.21 (d, 1H, *J* = 4.7 Hz, Ph-H), 8.14–8.17 (dd, 2H, *J*_*1*_ = 6.9 Hz, *J*_*2*_ = 2.0 Hz, Ph-H), 8.55 (s, 1H, triazole-H), and 8.82 (d, 1H, *J* = 4.7 Hz, N=C-H). ^13^C NMR (CDCl_3_, 75 MHz): *δ* 162.06, 156.31, 155.57, 154.08, 147.99, 131.06, 121.26, 114.73, 107.94, 67.93, 30.98, 19.07, and 13.70. ESI-HRMS calculated for C_15_H_17_N_4_O^+^ ([M + H]^+^): 269.1397; found: 269.1388.

7-(4-(Pentyloxy)phenyl)-[1,2,4]triazolo[1,5-a]pyrimidine **(6c)**:

Mp 102–103°C, yield 75%. ^1^H-NMR (CDCl_3_, 300 MHz): *δ* 0.96 (t, 3H, *J* = 7.0, CH_3_), 1.35–1.53 (m, 2H, CH_2_CH_2_), 1.80–1.89 (m, 2H, CH_2_), 4.07 (t, 2H, *J* = 6.5 Hz, CH_2_), 7.08–7.11 (dd, 2H, *J*_*1*_ = 7.0 Hz, *J*_*2*_ = 2.0 Hz, Ph-H), 7.21 (d, 1H, *J* = 4.7 Hz, Ph-H), 8.14–8.17 (dd, 2H, *J*_*1*_ = 6.9 Hz, *J*_*2*_ = 2.0 Hz, Ph-H), 8.55 (s, 1H, triazole-H), and 8.82 (d, 1H, *J* = 4.7 Hz, N=C-H). ^13^C NMR (CDCl_3_, 75 MHz): *δ* 162.06, 156.30, 155.56, 154.08, 147.99, 131.06, 121.25, 114.72, 107.94, 68.23, 28.65, 28.00, 22.31, and 13.91. ESI-HRMS calculated for C_16_H_19_N_4_O^+^ ([M + H]^+^): 283.1553; found: 283.1549.

7-(4-(Hexyloxy)phenyl)-[1,2,4]triazolo[1,5-a]pyrimidine **(6d)**:

Mp 96–97°C, yield 78%. ^1^H-NMR (CDCl_3_, 300 MHz): *δ* 0.92 (t, 3H, *J* = 7.0, CH_3_), 1.33–1.39 (m, 2H, CH_2_CH_2_), 1.44–1.54 (m, 2H, CH_2_), 1.79–1.89 (m, 2H, CH_2_), 4.07 (t, 2H, *J* = 6.5 Hz, CH_2_), 7.08–7.11 (dd, 2H, *J*_*1*_ = 6.9 Hz, *J*_*2*_ = 2.1 Hz, Ph-H), 7.21 (d, 1H, *J* = 4.7 Hz, Ph-H), 8.13–8.18 (dd, 2H, *J*_*1*_ = 6.9 Hz, *J*_*2*_ = 2.1 Hz, Ph-H), 8.55 (s, 1H, triazole-H), and 8.83 (d, 1H, *J* = 4.7 Hz, N=C-H). ^13^C NMR (CDCl_3_, 75 MHz): *δ* 162.07, 156.33, 155.58, 154.08, 148.01, 131.07, 121.26, 114.74, 107.94, 68.25, 31.41, 28.92, 25.54, 22.48, and 13.93. ESI-HRMS calculated for C_17_H_21_N_4_O^+^ ([M + H]^+^): 297.1710; found: 297.1702.

7-(4-(Benzyloxy)phenyl)-[1,2,4]triazolo[1,5-a]pyrimidine **(6e)**:

Mp 120–122°C, yield 87%. ^1^H-NMR (CDCl_3_, 300 MHz): *δ* 5.19 (s, 2H, CH_2_), 7.17–7.21 (dd, 2H, *J*_*1*_ = 6.9 Hz, *J*_*2*_ = 2.0 Hz, Ph-H), 7.26 (s, 1H, C=C-H), 7.36–7.41 (m, 5H, Ph-H), 8.15–8.18 (dd, 2H, *J*_*1*_ = 6.9 Hz, *J*_*2*_ = 2.0 Hz, Ph-H), 8.56 (s, 1H, triazole-H), and 8.83 (d, 1H, *J* = 4.7 Hz, N=C-H). ^13^C NMR (CDCl_3_, 75 MHz): *δ* 161.64, 156.36, 155.60, 154.05, 147.88, 136.05, 131.13, 128.61, 128.17, 127.32, 121.88, 115.19, 107.98, and 70.20. ESI-HRMS calculated for C_18_H_15_N_4_O^+^ ([M + H]^+^): 303.1240; found: 303.1227.

### 2.2 Pharmacology

#### 2.2.1 Animals

Male Kunming mice with weights from 18 to 22 g were used in all the pharmacological experiments. The mice were housed in the standard cages with 10 each, where a controlled temperature (25°C ± 2°C) with a 12 h light/dark cycle was maintained. All animals had free access to food and water. All experiments and procedures involving animals were carried out according to the Guide for the Care and Use of Laboratory Animals (National Academies Press, Washington, D.C., United States). Approval from the local medical ethical committee was obtained before the experiment.

#### 2.2.2 Maximal Electroshock Seizure Test

In the MES test, mice were subjected to ear stimulation using a 0.2 s 60-Hz 50-mA alternating current to elicit seizures. Tonic hind limb extension was considered as the seizure occurring, and the vanishing of tonic hind limb extension indicates the protection against the MES-induced seizures. Mice were injected intraperitoneally with test compounds at doses of 300, 100, and 30 mg/kg to evaluate their general anticonvulsant activity. Half and 4 h intervals were chosen to test the anti-MES activity. Based on the rough effective dose obtained in the aforementioned assay, compounds effective at the dose of 30 mg/kg were subjected to the quantitative assay to determine their median effective dose (ED_50_). A series of doses of the tested compounds were injected into the mice with 10 in one group. When not less than three doses were obtained at which the mice were protected in 10–90%, the respective ED_50_, and 95% confidence intervals were calculated using these data by probit analysis.

#### 2.2.3 Subcutaneous Pentylenetetrazole Test

In the PTZ test, mice were subcutaneously administered with PTZ at the dose of 85 mg/kg. At this dose, mice can be elicited a threshold seizure characterized by an episode of clonic spasms and tonic spasms. After being treated with test compounds at doses of 300, 100, and 30 mg/kg, the mice were considered protected if a single episode of clonic spasms of more than 5 s duration was not obtained. The quantitative anticonvulsant activity (ED_50_) in the PTZ model was determined and calculated with the same method described in the MES test.

#### 2.2.4 Neurotoxicity Screening

The rotarod test was used to evaluate the neurotoxicity of the synthesized compounds. Before the formal experiments, mice were trained to climb on a rotating rod of diameter 3.2 cm with the rotation speed of 10 rpm. Test compounds in the three doses were intraperitoneally injected into the trained mice. After 30 min, they were put on the rotating rod to observe their ability to maintain equilibrium. The rough neurotoxic dose was obtained in which mice cannot maintain equilibrium on the rod for at least 1 min. The quantitative neurotoxic dose (TD_50_) of the test compounds was measured and calculated with the same method described in the MES test.

#### 2.2.5 Bicuculline Test

In the BIC-induced seizure test, groups of 10 mice were administered with 30 mg/kg of test samples or vehicle. Then, 30 min later, the minimum dose of BIC (5.4 mg/kg) that could completely induce seizures for mice was subcutaneously injected into the test mice. The mice were placed into separate cages each, and the number of clonic seizures, tonic seizures, and mortality was recorded for 30 min.

#### 2.2.6 3-Mercaptopropionic Acid Test

In 3-MP–induced seizure test, groups of 10 mice were administered with 30 mg/kg of test samples or vehicle. After 30 min, the minimum dose of 3-MP (60 mg/kg) that could completely induce seizures for mice was intraperitoneally injected into the test mice. The mice were placed into separate cages each, and the number of clonic seizures, tonic seizures, and mortality was recorded for 30 min.

#### 2.2.7 Docking

Molecular docking was performed by Discovery Studio (release 2019). The three-dimensional (3D) GABA_A_ receptor binding with diazepam was obtained from Richter’s publication (Richter et al., 2012). The hydrogen atoms of the GABA_A_ receptor were added, and water and diazepam were removed. The 3D structures of test molecules were constructed by ChemDraw 16.0 software; then, they were transferred into the Discovery Studio platform to minimize energy. After the aforementioned preparation finished, the molecular docking was performed for the test molecules and GABA_A_. After the completion of molecular docking, the docking gave a batch of poses, which were ranked and selected by CDOCKER interaction energy. The lowest energy conformation of the ligand–receptor complex was evaluated, and the interaction type between GABA_A_ receptor and test molecules was analyzed by Discovery Studio (release 2019).

#### 2.2.8 Prediction of Drug Likeliness Parameters

Computational calculations were carried out to predict the drug likeliness properties of target compounds. The polar surface area (TPSA), number of rotatable bonds (n-ROTB), miLogP, number of hydrogen donor (HBD) and acceptor (HBA), and violations of Lipinski’s rule of five were calculated by using Molinspiration Property Calculator (Molinspiration Cheminformatics, 2021), which is a free online property calculating toolkit. Absorption (%ABS) was calculated using an equation % ABS = 109—(0.345 * PSA) (Zhao et al., 2002). LogBB, a ratio of the steady-state concentrations of a compound in the brain to that in the blood [i.e., log (C_brain_/C_blood_)], was calculated using Clark’s equation as follows: logBB = −0.0148 × PSA + 0.152 × clogP + 0.139 (Clark, 1999).

#### 2.2.9 Radioligand Receptor Binding Assay

The radioligand receptor binding studies were performed according to the procedure described by Ahmadi et al. (2014). Selected compounds were evaluated for their affinity to the GABA_A_ BZD receptor by the radioligand receptor binding assay. Male SD rats with weights ranging from 220 to 260 g were sacrificed after a spinal dislocation. The cerebral tissues except the brainstem was immediately removed and homogenized for 30 s in 10 times the volume of ice-cold Tris–HCl buffer (50 mM, pH 7.4). The homogenates were centrifuged at 1,000 g for 10 min. The resulting supernatant was centrifuged at 30,000 g for 20 min. The precipitate was washed three times with ice-cold buffer by re-suspension and re-centrifugation. All of the centrifugation procedures were performed at 4°C. The obtained membrane preparation was maintained under −4 Fahrenheit until it was used in 20 days. The protein concentration was estimated by the Bradford method. After the membrane preparation, saturation and competition experiments were conducted to obtain the inhibition of radioligand-specific binding (IC_50_) and affinity (K_i_) of the test compounds and diazepam. In the saturation experiment, 200 μg of membrane protein was incubated with nine different concentrations (0.25–25 nM) of [^3^H]- flumazenil at 30°C for 35 min. The total binding (TB) (receptor + radioligand), non-specific binding (NSB) (receptor + radioligand + excess diazepam), and specific binding (SB) (TB-NSB) were measured at the nine radioligand concentrations. The receptor-binding affinity (K_d_) of [^3^H]-flumazenil and the maximum binding capacity of BZD receptors (Bmax) were calculated from the saturation experiment data. In the competition experiment, 200 μg of membrane protein in Tris–HCl buffer (50 mM, pH 7.4) was incubated with 0.5 nM [^3^H]-flumazenil and different concentrations (50 pm-5 mM) of test compounds at 30°C for 35 min. After incubation, the assay was terminated by an ice bath. The activity of each tube was measured by a liquid scintillation counter after centrifugation and wash treatment. The competition curve was achieved by plotting the %SB of radioligand vs. the concentrations of test compounds. %SB = 100 (BC-NSB)/(TB-NSB). BC represents the binding in the presence of the test compound. IC_50_ inhibiting the [^3^H]-flumazenil-specific binding and affinity for BZD receptors (K_i_) of test compounds were calculated according to the Cheng–Prusoff equation. All the experiments were carried out in triplicates.

## 3 Results

### 3.1 Chemistry

**SCHEME 1:**
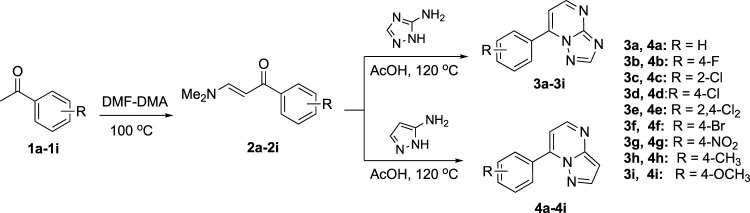
Synthetical route of target compounds **3a–3i** and **4a–4i**.

**SCHEME 2:**
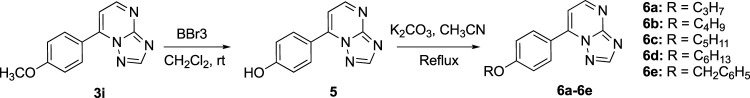
Synthetical route of target compounds **6a–6e**.

The synthetical route of compounds **3a–3i** was outlined in Scheme 1. The pathway started from the reaction of acetophenones (**1a–1i**) with *N,N*-dimethylformamide dimethyl acetal (DMF-DMA) at 100°C without solvent (Ma et al., 2018; Gado et al., 2020), which gave the intermediates (**2a–2i**). Next, compounds (**2a–2i**) were reacted with 3-amino-1,2,4-triazole and 3-aminopyrazole in AcOH to afford the target compounds **3a–3i** and **4a–4i**, respectively (Frizzo et al., 2014). Compounds (**6a–6e**) were prepared according to Scheme 2. Compound **3i** was treated with BBr_3_ in CH_2_Cl_2_ to give the demethylated derivative **5**, which was alkylated by haloalkanes to obtain the target compounds **6a–6e**. ^1^H-NMR, ^13^C NMR, and mass spectrometry were used to characterize the chemical structures of targets. The physical/analysis data in detail and the yield of the targets are provided in Section 2.

### 3.2 Evaluation of the Anticonvulsant Activity and Neurotoxicity

The anticonvulsant activities of targets were evaluated using seizure models induced by MES and PTZ. The neurotoxicity was evaluated *via* the rotarod test. Structurally, target molecules could be categorized into three groups: **3a–3i** (containing the triazole moiety), **4a–4i** (containing the pyrazole moiety), and **6a–6e** (containing the triazole moiety and higher hydrophobicity than the series of **3a–3i**). In each series, substituents on the benzene ring at position 7 were altered to optimize the anticonvulsant activity.

Initially, all the target compounds were screened at three dosages (30, 100, and 300 mg/kg). As exhibited in Table 1, compounds **3a**, **3b**, **3c**, **3e**, **3h**, and **3i** from the first series of compounds displayed medium-to-weak anticonvulsant activity at 100 or 300 mg/kg at 0.5 h in the MES test. None in the second series of compounds (**4a–4i**) displayed anticonvulsant activity even at the highest dose of 300 mg/kg in the MES test. On the contrary, all compounds in the series of **6a–6e** showed anticonvulsant activity in three dosages. Compounds **6c**, **6d**, and **6e** displayed the best protective effect at the dosage of 30 mg/kg and remained active at 100 or 300 mg/kg after 4 h.

**TABLE 1:** Anticonvulsant activity and neurotoxicity of compounds **3a–3i, 4a–4i, and 6a–6e** administered intraperitoneally in mice.


Compound	R	Intraperitoneal injection in mice^a^					
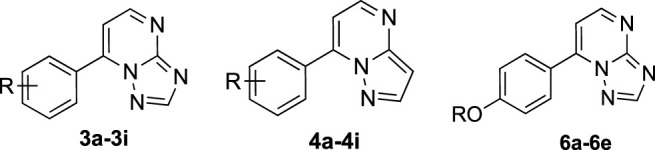		MES screening^b^		PTZ screening^c^		NT screening^d^	
		0.5 h	4 h	0.5 h	4 h	0.5 h	4 h
**3a**	H	300	—	100	—	—	—
**4a**	H	—	—	—	—	—	—
**3b**	4-F	300	—	300	—	—	—
**4b**	4-F	—	—	—	—	—	—
**3c**	2-Cl	100	—	100	—	—	—
**4c**	2-Cl	—	—	—	—	—	—
**3d**	4-Cl	—	—	—	—	—	—
**4d**	4-Cl	—	—	—	—	—	—
**3e**	2,4-Cl_2_	100	—	100	—	—	—
**4e**	2,4-Cl_2_	—	—	300	—	—	—
**3f**	4-Br	—	—	—	—	—	—
**4f**	4-Br	—	—	—	—	—	—
**3g**	4-NO_2_	—	—	—	—	—	—
**4g**	4-NO_2_	—	—	—	—	—	—
**3h**	4-CH_3_	300	—	100	—	—	—
**4h**	4-CH_3_	—	—	300	—	—	—
**3i**	4-OCH_3_	300	—	100	—	—	—
**4i**	4-OCH_3_	—	—	300	—	—	—
**6a**	C_3_H_7_	300	—	300	—	—	—
**6b**	C_4_H_9_	100	—	300	—	—	—
**6c**	C_5_H_11_	30	300	30	—	—	—
**6d**	C_6_H_13_	30	100	30	—	300	—
**6e**	CH_2_C_6_H_5_	30	300	30	—	300	—

a Animal number used = 3–5. Dosages administered were 30, 100, and 300 mg/kg. The figure in the table indicates the minimum dose, whereby bioactivity was demonstrated in half or more of the mice. The animals were examined at 0.5 and 4 h after injection was administered. A dash indicates the absence of anticonvulsant activity and neurotoxicity at the maximum dose administered (300 mg/kg).

b Maximal electroshock test.

c Subcutaneous pentylenetetrazole test.

d Neurotoxicity screening (rotarod test).

The performance of designed compounds in the PTZ model was generally similar to that in the MES test. Compounds **3a**, **3b**, **3c**, **3e**, **3h**, and **3i** from the first series of compounds displayed protection at the dosage of 100 or 300 mg/kg in the PTZ model at 0.5 h. Compounds **4e**, **4h**, and **4i** in the second series exhibited anticonvulsant activity at the highest dose of 300 mg/kg at 0.5 h intervals. Compounds **6a** and **6b** protected the mice against PTZ-induced seizure at 300 mg/kg at 0.5 h interval. Compounds **6c**, **6d**, and **6e** showed protection for mice at the dose of 30 mg/kg at 0.5 h interval.

None of the target molecules except **6d** and **6e** exhibited neurotoxicity at the maximum dosage of 300 mg/kg in the rotarod test. Some mice injected with **6d** or **6e** in 300 mg/kg could not maintain balance on a rotating rod. In addition, no compounds were observed to have side effects in common with antiepileptics, such as sedation, hypnosis, and anxiety (with features of bradykinesia or running) at the dosage of 100 mg/kg.

According to the visible antiepileptic activity of compounds **6c**, **6d**, and **6e** indicated in the initial trials, the quantitative evaluation trials were conducted to obtain the ED_50_ and TD_50_ values. The quantitative data of compounds **6c**, **6d**, and **6e** and those of the positive drugs valproate, carbamazepine, and diazepam, are listed in Table 2. Compound **6d** with an ED_50_ value of 15.8 mg/kg was found to have the strongest effect against MES-induced seizures in mice. Meanwhile, compounds **6c** and **6e** displayed ED_50_ values of 25.3 and 38.0 mg/kg, respectively. All compounds tested were more potent than valproate but less active than that of carbamazepine and diazepam in the MES-induced seizure model. In the PTZ model, compounds **6c**, **6d**, and **6e** gave ED_50_ values of 23.7, 14.1, and 28.4 mg/kg, respectively. They were more active than valproate and carbamazepine but less active than that of diazepam in the PTZ-induced seizure model.

**TABLE 2:** Quantitative anticonvulsant date of **6c**, **6d**, and **6e** (anti-MES and anti-PTZ) in mice administered intraperitoneally.

Compound	ED_50_ ^a^		TD_50_ ^b^	PI^c^	
	MES	scPTZ		MES	scPTZ
**6c**	25.3 (23.0–27.9)	23.7 (21.5–26.0)	380 (346–418)	15.0	16.0
**6d**	15.8 (14.3–17.4)	14.1 (13.1–15.3)	317 (290–346)	20.1	22.5
**6e**	38.0 (34.6–41.8)	28.4 (25.8–31.2)	366 (333–403)	9.6	12.9
Valproate	264 (247–338)	149 (123–177)	418 (369–450)	1.6	2.8
Carbamazepine	9.8 (8.9–10.8)	>100	44.0 (40.2–48.1)	4.5	<0.44
Diazepam	10.1 (8.9–11.5)	0.5 (0.3–0.7)	3.3 (2.9–3.7)	0.33	6.6

a ED_50_—median effective dosage needed to assure anticonvulsant protection in 50% animals.

b TD_50_—median toxic dosage eliciting minimal neurological toxicity in 50% animals.

c PI, protective index (TD_50_/ED_50_).

As for the neurotoxicity, all synthesized compounds, including the standard drug valproate, showed neurotoxicity at very high dosages (TD_50_ ≥ 317 mg/kg). However, the TD_50_ values for the positive controls carbamazepine and diazepam were 44.0 and 3.3 mg/kg, respectively, being remarkably less than those of the synthesized compounds. Based on the obtained results from MES, PTZ, and rotarod tests, the protective index (PI = TD_50_/ED_50_) was calculated and is presented in Table 2. The results revealed that the designed compounds were able to display good anticonvulsant activity with a high safety profile. Although the anti-MES and anti-PTZ activities of the compounds **6c–6e** were lower than those of diazepam, their PI values were significantly higher than those of the latter.

### 3.3 Antiepileptic Activity Evaluation of Compound **6d** in Other Seizure Models

To further confirm the antiepileptic activity, two seizure models induced by 3-MP and BIC were used. 3-MP, a competitive inhibitor of glutamate decarboxylase (GAD), can decrease the level of GABA in the brain by inhibiting its synthesis (Crick et al., 2014; Enrique et al., 2017). As shown in Table 3, 3-MP induced convulsions in 100% of the mice at 60 mg/kg. 3-MP and **6d** combination therapy showed complete inhibition on mice’s clonic seizures, tonic seizures, and death in comparison with the results following treatment of 3-MP alone. BIC could induce seizures in mice as a competitive GABA_A_ receptor antagonist (Velisek, 2006). BIC caused seizures in 100% of the mice at 5.4 mg/kg. As indicated in Table 4, carbamazepine significantly inhibited tonic seizure and death. Similarly, compound **6d** inhibited tonic seizure, death (both from 100% to 0%, *p* < 0.001 for seizures and death), and clonic seizure (from 100% to 10%, *p* < 0.05) significantly. The superiority of compound **6d** in inhibiting clonic seizures, tonic seizures, and death in seizure models caused by 3-MP and BIC, compared to carbamazepine, further identified the antiepileptic effect of **6d**.

**TABLE 3:** Effect of compound **6d** on 3-mercaptopropionic acid-induced seizures in mice.

Compound	Dose (mg/kg)	Test time (h)	Clonic seizure (%)	Tonic seizure (%)	Lethality (%)
DMSO	—	0.5	100	100	70
Carbamazepine	30	0.5	100	0***	0**
**6d**	30	0.5	0***	0***	0**

Results are expressed as the percentage of animals showing clonic and tonic convulsions and death in all animals tested. Ten mice were included in each group. Significance was determined with Fisher’s exact test. **p < 0.01 and ***p < 0.001 vs. group of 3-MP (60 mg/kg).

**TABLE 4:** Efficacy of compound **6d** on bicuculline-induced seizures in mice.

Compound	Dose (mg/kg)	Test time (h)	Clonic seizure (%)	Tonic seizure (%)	Lethality (%)
DMSO	—	0.5	100	100	100
Carbamazepine	30	0.5	100	0***	20**
**6d**	30	0.5	10**	0***	0***

Results are expressed as a percentage of animals showing clonic and tonic convulsions and death among all animals tested. The number of animals tested in each group was ten. Significance was determined with Fisher’s exact test. **p < 0.05 and ***p < 0.001 vs. group of BIC (5.4 mg/kg).

### 3.4 Study on the Possible Mechanism of Action

As is known, diazepam belongs to BZDs. The anticonvulsant activity of diazepam can be reversed by the BZD-receptor antagonist flumazenil (Kim et al., 2020). To understand the possible mechanism of the anticonvulsant activity of the most potent compound **6d**, we checked if the protection activity of the compound **6d** in the MES and PTZ tests could be antagonized by flumazenil. As shown in Table 5, administration of compound **6d** at 30 mg/kg alone gave full protection to mice against the MES and PTZ-induced seizures, while pretreatment of flumazenil completely reversed the anti-MES and anti-PTZ activities of compound **6d**. These results suggested that the anticonvulsant action of compound **6d** might be related to BZD receptors.

**TABLE 5:** Effects of flumazenil (FMZ) and thiosemicarbazide (TSC) on the anticonvulsive action of **6d** against MES and PTZ-induced seizures in mice.

Compound	Dose (mg/kg)	Test time (h)	MES model	PTZ model
**Saline + 6d**	30	0.5	5/5^c^	5/5
**FMZ + 6d** ^a^	30	0.5	0/5***	0/5***
**Saline + 6d**	30	0.5	5/5	5/5
**TSC + 6d** ^b^	30	0.5	0/5***	0/5***

The number of animals tested per group was five. Significance was determined by Fisher’s exact test. ***p < 0.001 vs. group of **6d** (pretreatment with saline).

a Mice was pretreated with flumazenil (10 mg/kg, single administration) 15 min before seizure induction.

b Mice was pretreated with TSC (25 mg/kg/day for 3 days).

c Number of animals protected/number of animals tested.

Thiosemicarbazide (TSC), a competitive inhibitor of the GABA synthesis enzyme, can decrease the GABA level in the brain by inhibiting the synthesis of GABA (Collins, 1973). The influence of TSC on the antiseizure activity of **6d** was investigated to further confirm that the GABAergic system was involved in the antiseizure mechanism of **6d**. As displayed in Table 5, the normal mice were completely protected in the MES model at 30 mg/kg, while the effects vanished in the treated mice. The anticonvulsant activity of **6d** was completely reversed after the pretreatment with TSC at the dosage of 25 mg/kg for 3 days in the MES and PTZ models. Based on this finding, it could be concluded that compound **6d** acts its antiepileptic activity by regulating the GABA function in the brain.

The radioligand receptor–binding study is a very powerful tool in the study of receptors and their ligands. To verify the hypothesis that these compounds are binding and affecting the GABA_A_ receptor chloride ionophore complex *via* the BZP receptor, the affinity of compound **6d** to the BZD receptor was evaluated using [^3^H] flumazenil as the specific ligand. The results showed that compound **6d** and diazepam have an affinity to the BZD receptor in nanomolar concentrations (IC_50_ = 8.4 and 1.3 nM and K_i_ values = 3.9 and 0.61 nM, respectively). This result further confirmed that the anticonvulsant activity of **6d** was mediated by BZD receptors (Table 6).

**TABLE 6:** Binding affinity of compound **6d** and diazepam competing on ^3^H-flumazenil to the benzodiazepine receptor.

Compound	IC_50_ (95% CI nM)	K_i_ (nM)
**6d**	8.4 (2.7–21.2)	3.9
Diazepam	1.3 (0.48–2.93)	0.61

### 3.5 Molecular Docking Study

**FIGURE 3:**
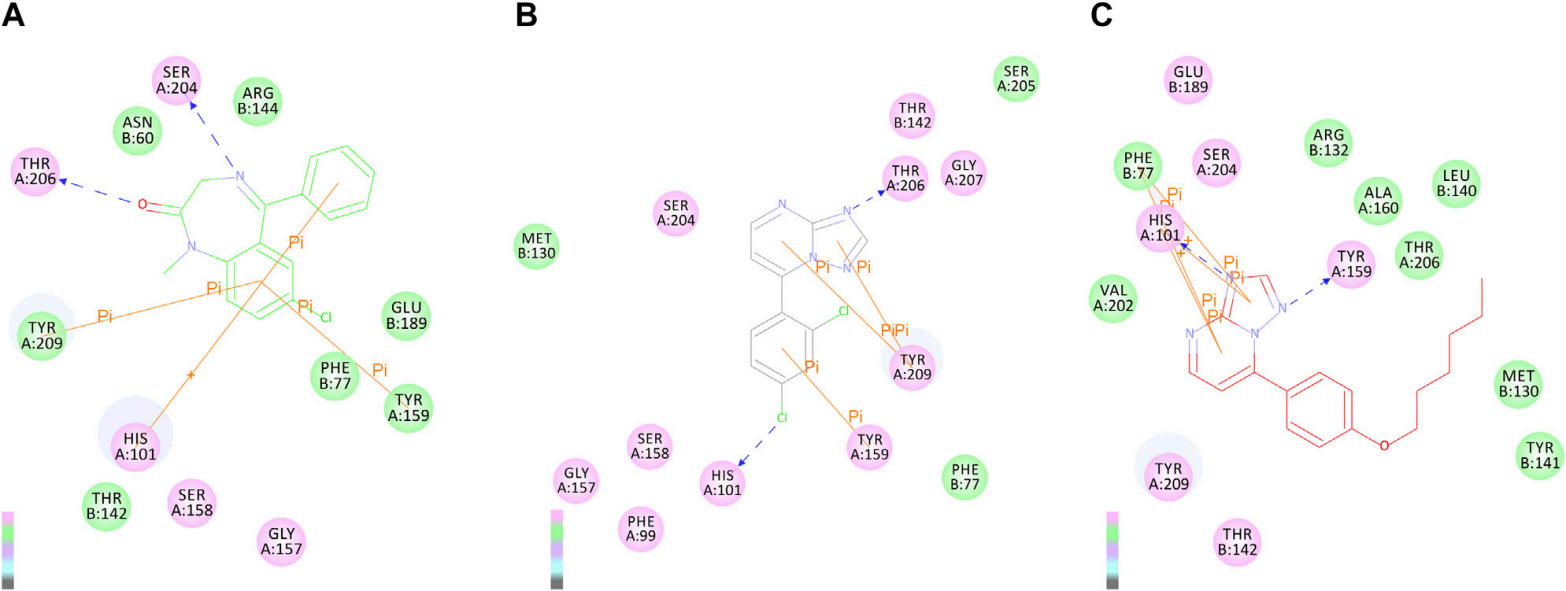
2D binding modes of diazepam **(A)**, compound **4e (B)**, and **6d (C)** in the BZD-binding pocket of the GABA_A_ receptor (α1β2γ2).

To explore the interaction pattern of the synthesized compounds to the BZD-binding site of the GABA_A_ receptor, the docking of active compounds **3e** and **6d** with the BZD-binding site of the GABA_A_ receptor (α1β2γ2) was conducted by Discovery Studio 4.0 Client. Interactions of the diazepam and compounds **3e** and **6d** with amino acids of BZD-binding pocket of the GABA_A_ receptor are illustrated and shown in Figure 3. As shown in Figure 3, the most important residues in the binding mode of diazepam are Ser204, Thr206, Tyr209, His101, and Tyr159. These results are consistent with those reported by Ernst and Foroumadi (Richter et al., 2012; Mohammadi-Khanaposhtani et al., 2016). Interactions of compound **3e** and the GABA_A_ receptor were identical with those of diazepam. Critical amino acid residues SER204, Thr206, Tyr209, His101, and Tyr159 were all involved, but the amino acid residue Thr206 formed a hydrogen bonding with the N atom in position 3. The benzene ring in compound **3e** was responsible for π–π interaction with His101 and Tyr159. The binding model of compound **6d** was different from diazepam and compound **3e**. Amino acid residues Phe77, His101, Arg132, and Tyr 209 were involved in the interactions between compound **6d** and the GABA_A_ receptor. It is worth mentioning that the N atom in position 3 was also a critical one that was involved in hydrogen-bonding interaction with His 101.

### 3.6 Prediction of Drug-Like Parameters

Drug-like property is an important basis for evaluating the potential of drug development. The drug-like properties of targeted compounds, such as molecular weight (MW), octanol/water partition coefficients (ClogP), the number of H-bond donors (HBD), the number of H-bond acceptors including (HBA), the number of rotatable bonds (ROTB), and the polar surface area (tPSA), were predicted by the computational study. The PSA of a molecule effectively represents the portion of its surface belonging to polar atoms, such as oxygen, nitrogen, and attached hydrogen, and is a descriptor related to the passive molecular transport of a molecule through membranes. From the PSA, intestinal absorption (%ABS) was calculated according to the algorithm described by Zhao et al. (2002). It has been proven by Palm et al. (1996) that drugs with a PSA value below 60 were completely absorbed in the intestine based on Caco-2 cell studies. The PSA values of most target compounds were all below 60 except for compounds **3g** and **4g**, and the level of intestinal absorption (%ABS) was bigger than 90% except compounds **3g** and **4g**. The following conclusions can be drawn that these compounds might display good transport properties in the intestines.

The “rule of five” is a set of simple molecular descriptors, which was established by Lipinski, which states that most “drug-like” molecules have common parameters, including LogP ≤ 5, Mw ≤ 500, HAB ≤ 10, HBD ≤ 5, and ROTB ≤ 10 (Lipinski et al., 2001). Molecules that violate one of the aforementioned rules may have bioavailability problems. It can be seen from Table 7 that all synthetic compounds meet all parameters of Lipinski and have good drug-likeliness.

**TABLE 7:** Pharmacokinetic parameters important for good oral bioavailability and drug likeness of targets compounds **3a–3i**, **4a–4i**, and **6a–6e**.

Compound	% ABS	TPSA (Å^2^)	n-ROTB	MW	miLogP	HBD	HBA	Lipinski’s violation	LogBB
**Rule**	—	—	<10	<500	≤5	≤5	≤10	≤1	—
**3a**	94.1	43.09	1	196.21	1.51	0	4	0	−0.27
**3b**	94.1	43.09	1	214.20	1.68	0	4	0	−0.24
**3c**	94.1	43.09	1	230.66	2.19	0	4	0	−0.17
**3d**	94.1	43.09	1	230.66	2.15	0	4	0	−0.17
**3e**	94.1	43.09	1	265.10	2.80	0	4	0	−0.07
**3f**	94.1	43.09	1	275.11	2.32	0	4	0	−0.15
**3g**	78.3	88.92	2	241.21	1.47	0	7	0	−0.95
**3h**	94.1	43.09	1	210.24	1.96	0	4	0	−0.20
**3i**	90.9	52.33	2	226.24	1.57	0	5	0	−0.40
**4a**	98.6	30.20	1	195.22	2.25	0	3	0	0.03
**4b**	98.6	30.20	1	213.22	2.41	9	3	0	0.06
**4c**	98.6	30.20	1	229.67	2.88	0	3	0	0.13
**4d**	98.6	30.20	1	229.67	2.92	0	3	0	0.14
**4e**	98.6	30.20	1	264.12	3.53	0	3	0	0.23
**4f**	98.6	30.20	1	274.12	3.06	0	3	0	0.16
**4g**	82.8	76.02	2	240.22	2.21	0	6	0	−0.65
**4h**	98.6	30.20	1	209.25	2.69	0	3	0	0.10
**4i**	95.4	39.43	2	225.25	2.30	0	4	0	−0.09
**6a**	90.9	52.33	4	254.29	2.45	0	5	0	−0.26
**6b**	90.9	52.33	5	268.32	3.01	0	5	0	−0.18
**6c**	90.9	52.33	6	282.35	3.52	0	5	0	−0.10
**6d**	90.9	52.33	7	296.37	4.02	0	5	0	−0.02
**6e**	90.9	52.33	4	302.34	3.17	0	5	0	−0.15

%ABS, percentage of absorption; TPSA, topological polar surface area; MW, molecular weight; miLogP, Log P calculated by Molinspiration online services; HBD, number of H-bond donors; HBA, number of H-bond acceptors; n-ROTB, number of rotatable bonds; LogBB, the ratio of the steady-state concentrations of a compound between the brain and the blood [i.e., log (C_brain_/C_blood_)]. logBB = −0.0148 × PSA + 0.152 × clogP + 0.139.

Good blood–brain barrier (BBB) permeability is necessary for CNS drugs (Fu, 2018) To predict the BBB penetration, logBB of the compounds was calculated according to the algorithm described by Clark (1999), which refers to the ratio of the steady-state concentrations of compounds in the brain and blood [i.e., log (C_brain_/C_blood_)]. As reported, compounds with logBB < −1.0 are poorly distributed in the brain (Abraham et al., 1997). The logBB values of synthesized compounds are calculated and presented in Table 6. The measured logBB values for compounds **3a–3i**, **4a–4i**, and **6a–6e** were ranging from −0.95 to 0.23. The most active compound **6d** had logBB of −0.05, being the acceptable value for a CNS-active compound.

## 4 Discussion

Diazepam, a classic benzodiazepine drug, is widely used to treat epilepsy, especially the epileptic status (Calcaterra and Barrow, 2014). However, the side effects of diazepam including drowsiness, motor coordination disorders, and drug dependence limit its application in the clinics (Wu et al., 2016). To obtain new anticonvulsants, which are highly safe, triazole derivatives **3a–3i** were designed and synthesized based on the pharmacophoric features of benzodiazepine agonists. The MES and PTZ models, as the most popular and widely used animal seizure models, were used in our anticonvulsant activity screen. As reported, the MES test is considered to be a predictor of the possible therapeutic effect on generalized tonic–clonic seizures, while the PTZ test represents an effective model for selecting candidates for human generalized myoclonic seizures (Loscher, 2011).

The preliminary study showed that some triazole derivatives synthesized displayed anticonvulsant activity in both seizure models. To verify whether the triazole moiety is necessary for the anticonvulsant activity, a series of pyrazole derivatives (**4a–4i**), as their bioisosterics, were prepared, and their anticonvulsant activity was screened. It was disappointing, but as expected, pyrazole derivatives (**4a–4i**) did not display any anticonvulsant activity in the MES test even at the highest dose. This suggested that triazole plays a critical role in anticonvulsant activity.

Considering that the BBB permeability is necessary for the compounds to work *in vivo*, we prepared additional compounds (**6a–6e**) with higher hydrophobicity. It is gratifying that these compounds (**6a–6e**) with higher hydrophobicity showed better performance in the seizure models. Compound **6d**, whose ED_50_ values are 15.8 and 14.1 mg/kg against MES- and PTZ-induced seizures, respectively, in mice, was the most promising one in this study. Hydrophobicity is an important parameter for the anticonvulsant activity of triazole derivatives (Song and Deng, 2018; Aggarwal and Sumran, 2020). Many triazole derivatives holding a suitable aryl or alkyl group gave a better anticonvulsant activity in their analogs (Deng et al., 2014a; Deng et al., 2014b). From Table 1, it was noticeable that the hydrophobicity of compounds **6a–6e** affects their anticonvulsant activity. For compounds **6a–6d**, with the extension of the alkyl chain, the hydrophobicity of the compounds increased gradually, and the activity increased accordingly. The ClogP values of **6a**, **6b**, **6c**, **6d**, and **6e** were calculated by ChemDraw 16.0 software as 1.56, 2.09, 2.62, 3.15, and 2.27, respectively. The bigger hydrophobicity of **6d** gives it higher BBB permeability and higher *in vivo* anticonvulsant activity.

The comparison of the PTZ and MES test results obtained showed that all active molecules possessed better performance in the PTZ model than in the MES model. Some studies indicated that PTZ diminishes the GABAergic function (Macdonald and Barker, 1977), through competitive antagonism to the BZD receptor (Rehavi et al., 1982). Correspondingly, drugs that enhance GABA_A_ receptor neurotransmission, such as BZDs (Shafie et al., 2020; Navidpour et al., 2021), can inhibit seizures induced by PTZ. PTZ, 3-MP, and BIC also induced seizures through GABA_A_ receptor neurotransmission. The ability of compound **6d** to block PTZ, 3-MP, and BIC-induced seizures may be attributed to its modulatory effect on GABA_A_ receptor neurotransmission.

The anticonvulsant effect of compound **6d** against MES- and PTZ-induced seizures was completely reversed by the BZD-receptor antagonist flumazenil, which suggested that BZD receptors might be involved in the anticonvulsant effect of compound **6d**. The radioligand-binding assay further confirmed that the anticonvulsant activity of compound **6d** can be attributed to the direct modulation of the BZD receptor. This result is consistent with our initial design and also supported by the docking study. Interactions of compound **3e** and GABA_A_ receptor were identical with those of diazepam. The amino acid residue Thr206 formed a hydrogen bond with the N atom in position 3, which was missed in the inactive derivatives **4a–4i**. The binding model of compound **6d** was different from the diazepam and compound **3e**. But the N atom in position 3 was also a critical atom, which was involved in a hydrogen bonding interaction with His101 in the interactions of compound **6e** and the GABA_A_ receptor.

BZDs represent one of the important classes of anticonvulsant agents with excellent activity against generalized tonic–clonic and partial seizures. It has been clarified that it works by binding to the GABA_A_ receptors. This binding increases the frequency of chloride ion channel opening, which facilitates the inhibitory effects of GABA. However, certain side effects are associated with the short- and long-term use of benzodiazepines, which includes sedation, confusion, drowsiness, ataxia, and memory impairment (Schmitz, 2016). Since chlordiazepoxide was found in 1960, a large number of BZD drugs have been found and approved, among which clobazam, clonazepam, nordazepam, nimetazepam, lorazepam, and diazepam are used in the treatment of epilepsy. All of them shared the common skeleton, that is, the benzodiazepine moiety, and gave the common SARs (Riss et al., 2008). In the last decade, new BZDs were designed and reported to find novel anticonvulsant agents with less adverse effects (Arora et al., 2020; Maramai et al., 2020). In 2017, Shao et al. (2018)reported a series of 7-(benzylamino)-1H-benzo[b] [1,4]diazepine-2,4(3H,5H)-dione derivatives. It was found that the most active compound was substituted by 4-fluoro, which holds an ED_50_ value of 36.5 mg/kg in the MES test and 68.2 mg/kg in the scPTZ test. Compared to that, compound **6d** reported here exhibited better anti-MES and anti-PTZ activities. More important is that the structural skeleton jumped out of the bondage of benzodiazepine, which provides a new skeleton for the research of antiepileptic drugs based on the BZD-binding site of the GABA receptor.

## 5 Conclusion

In conclusion, the design, synthesis, and *in vivo* anticonvulsant activity of a series of triazole derivatives (**3a–3i**), pyrazole-contained bioisosterics (**4a–4i**), and analogs with higher hydrophobicity (**6a–6e**) were reported. More than half in the series of **3a–3i** and all in the series of **6a–6e** showed anticonvulsant activity in both MES- and PTZ-induced seizure models. On the contrary, pyrazole-containing bioisosterics (**4a–4i**) showed weak or no protective effects in both models. The triazole moiety played an important role in their anticonvulsant activity. This supposition was supported by the docking study. Among the synthesized compounds, the triazole derivative **6d** was the most potent compound in the MES and PTZ tests. Although the anti-MES and anti-PTZ activities of the compound **6d** were lower than those of diazepam, their PI values were significantly higher than those of the latter. Moreover, the experimental investigation of the compound **6d’s** mechanism of action suggested that compound **6d** works as an anticonvulsant agent *via* regulating the GABA function such as the activation of the BZD receptor. Prominent anticonvulsive performance and low neurotoxicity, combined with excellent drug-like properties of the triazolopyrimidines reported in this work, indicate their potential in the development of new anticonvulsant agents.

## Data Availability

The original contributions presented in the study are included in the article/Supplementary Material; further inquiries can be directed to the corresponding authors.
