# Supplementary material for: Design, Synthesis, and Evaluation of Anticonvulsant Activities of New Triazolopyrimidine Derivatives
Source: Front Chem. 2022 Jun 23;10:925281. doi: 10.3389/fchem.2022.925281 (PMC9260081; doi:10.3389/fchem.2022.925281)
Supplement: Supplementary file 1 [file DataSheet1.PDF]

# *Supplementary Material*

## 1 Supplementary Data

Supplementary Material should be uploaded separately on submission. Please include any supplementary data, figures and/or tables. All supplementary files are deposited to FigShare for permanent storage and receive a DOI.

Supplementary material is not typeset so please ensure that all information is clearly presented, the appropriate caption is included in the file and not in the manuscript, and that the style conforms to the rest of the article. To avoid discrepancies between the published article and the supplementary material, please do not add the title, author list, affiliations or correspondence in the supplementary files.

## 2 Supplementary Figures and Tables

For more information on Supplementary Material and for details on the different file types accepted, please see here. Figures, tables, and images will be published under a Creative Commons CC-BY licence and permission must be obtained for use of copyrighted material from other sources (including re-published/adapted/modified/partial figures and images from the internet). It is the responsibility of the authors to acquire the licenses, to follow any citation instructions requested by third-party rights holders, and cover any supplementary charges.

### 2.1 Supplementary Figures

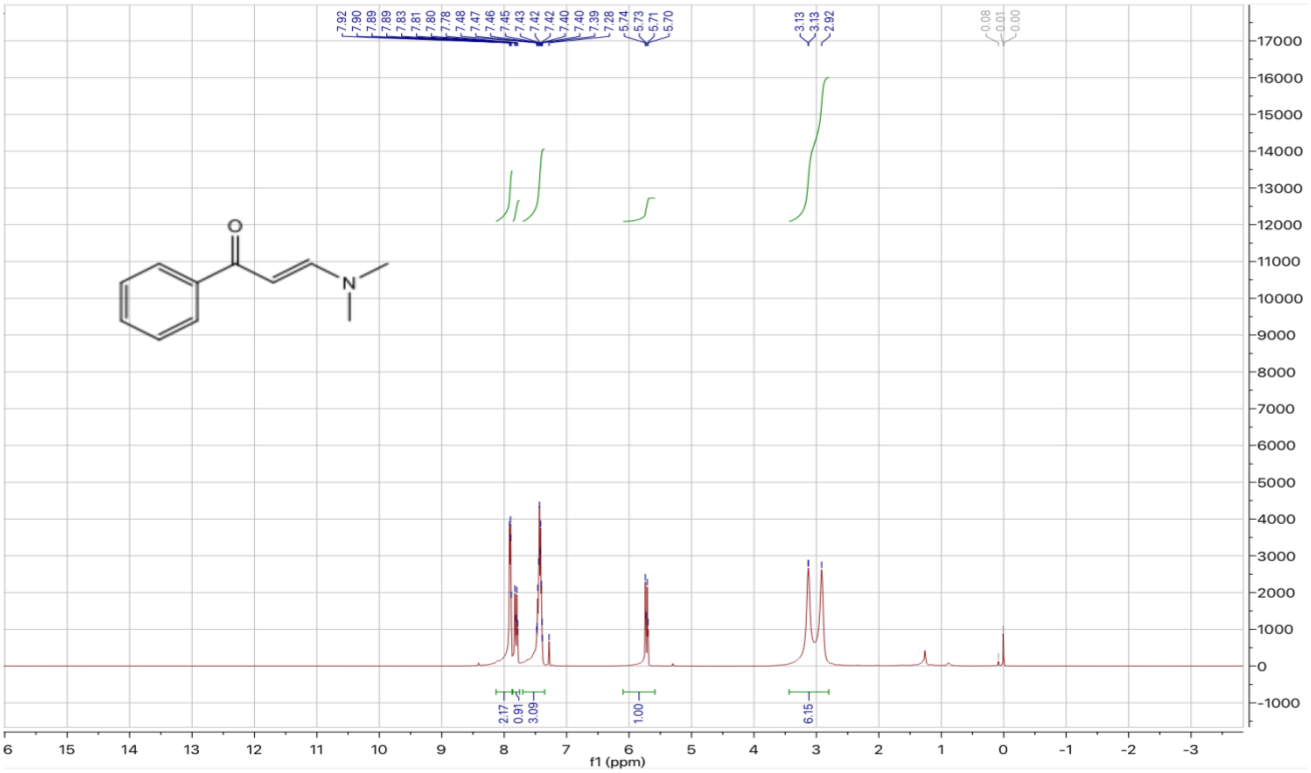

Figure 1 The H NMR spectrum of compound 2a

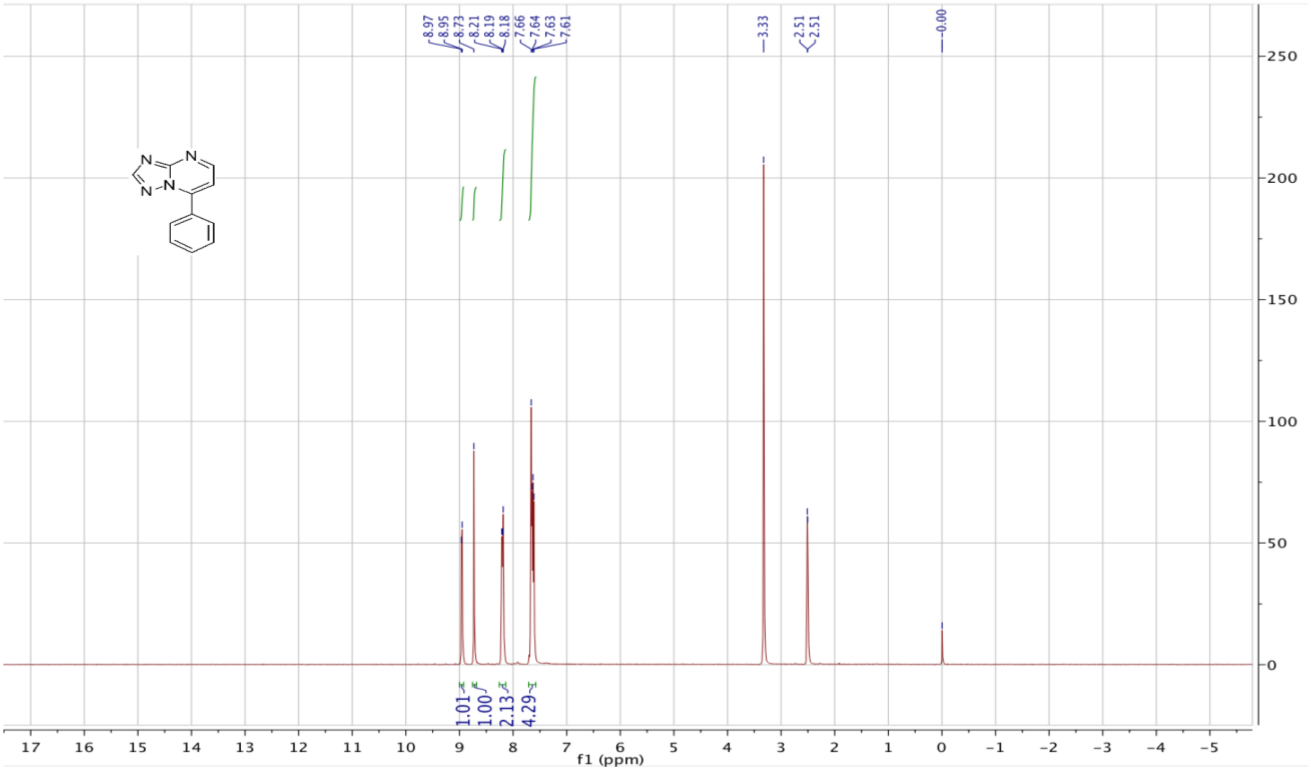

Figure 2 The H NMR spectrum of compound 3a

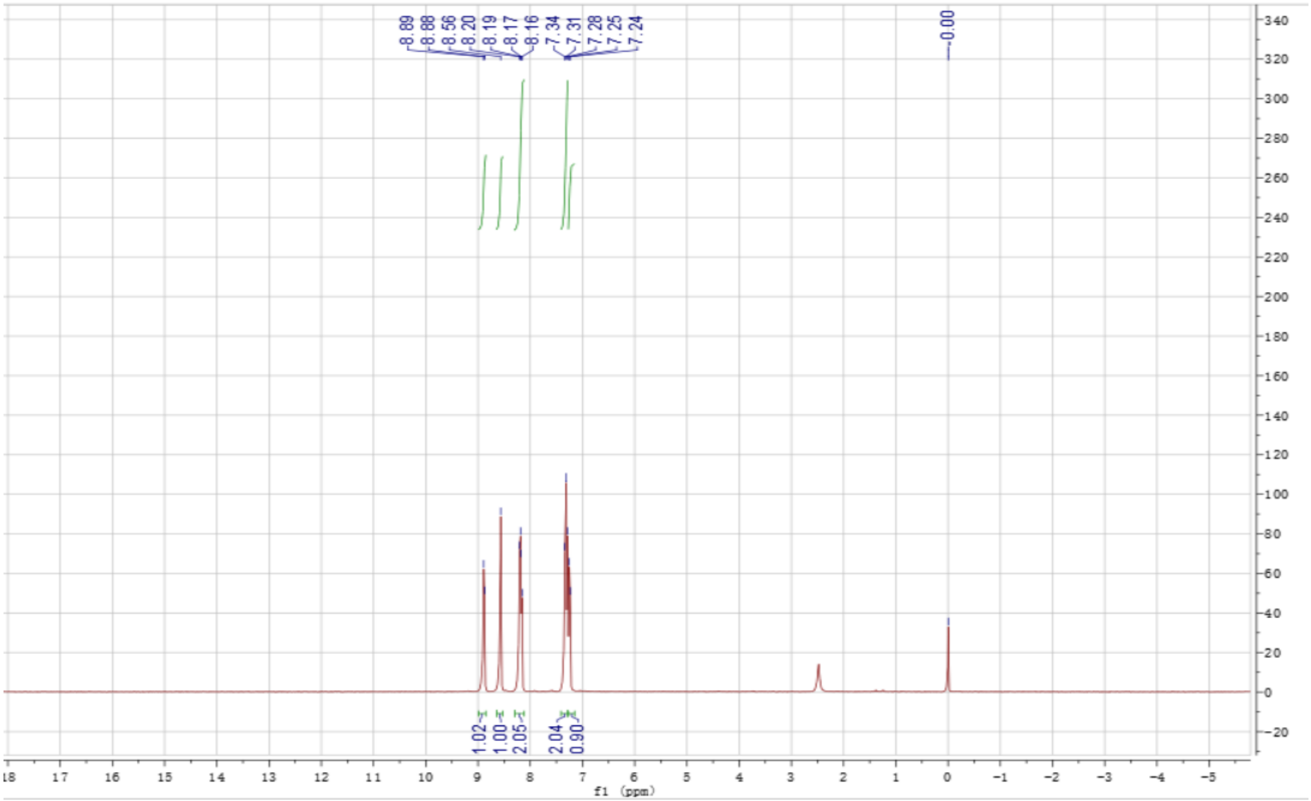


Figure 3 The H NMR spectrum of compound 3b

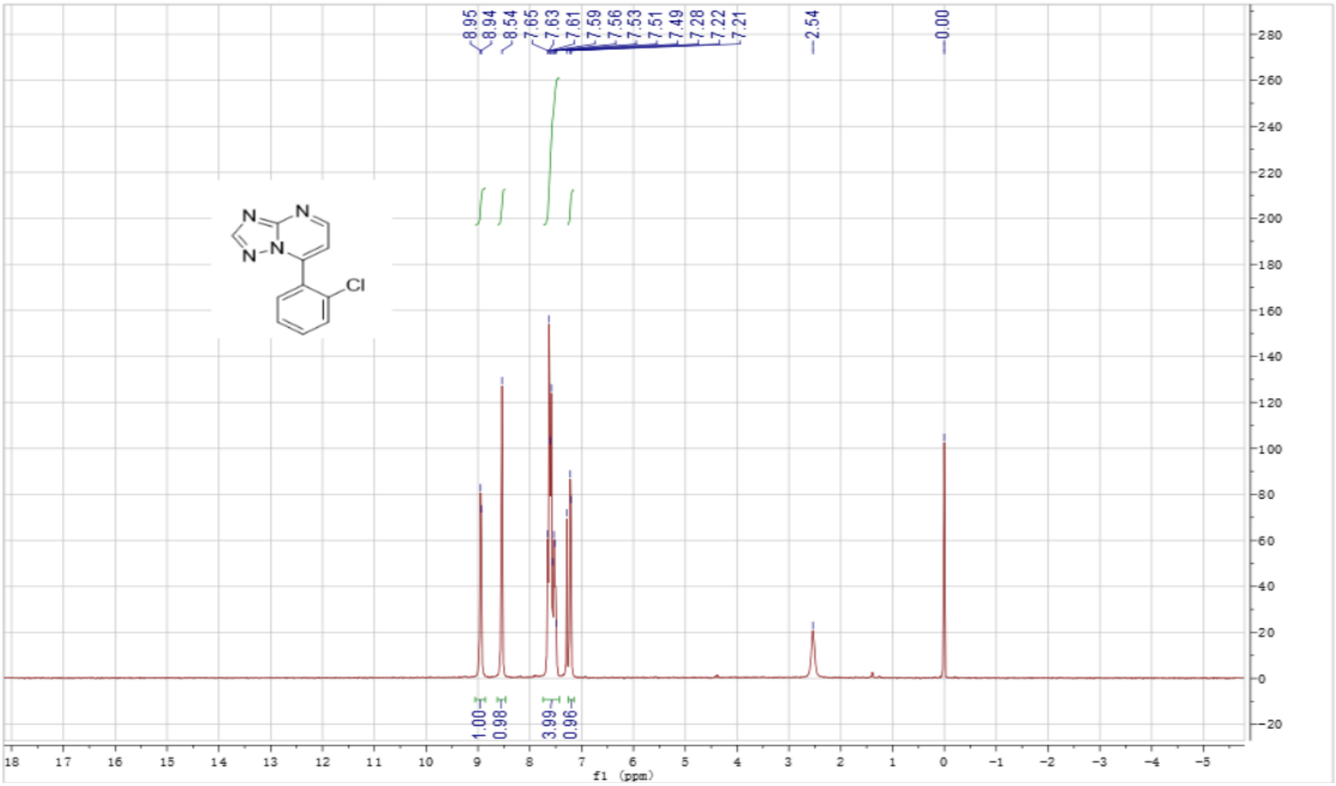


Figure 4 The H NMR spectrum of compound 3c

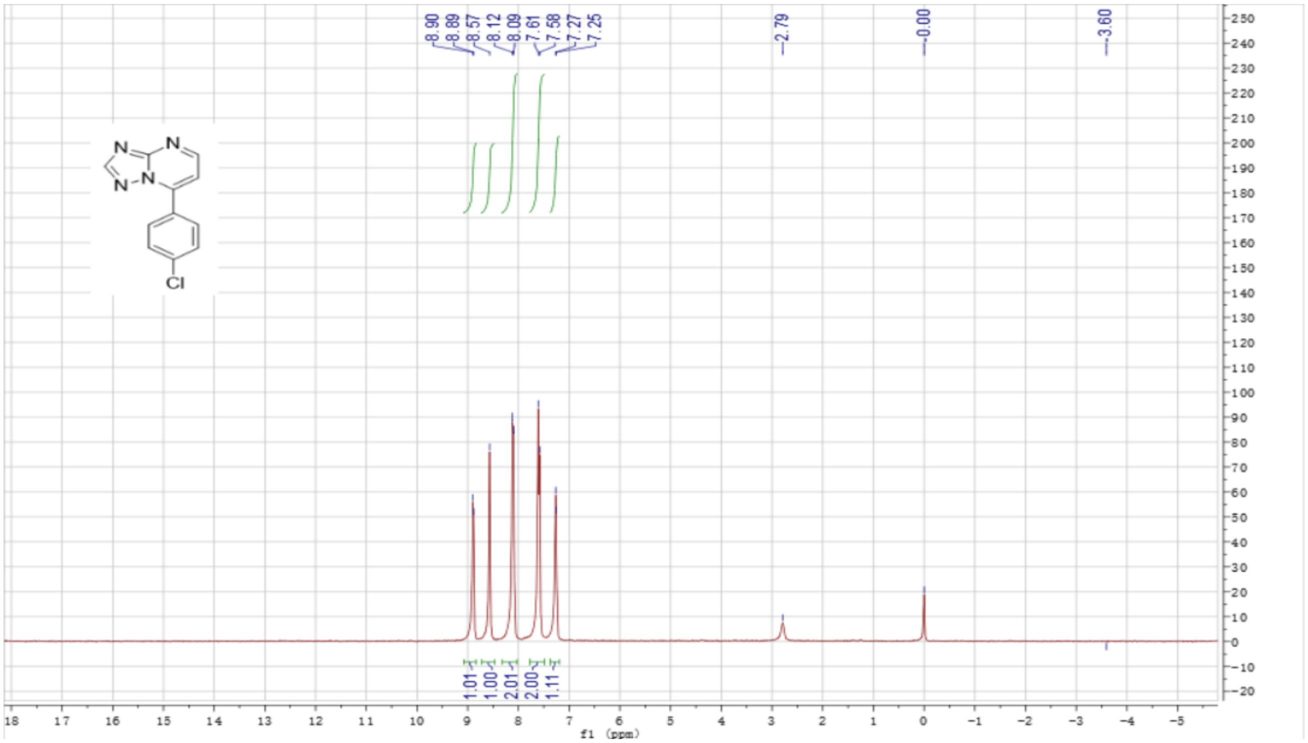

Figure 5 The H NMR spectrum of compound 3d

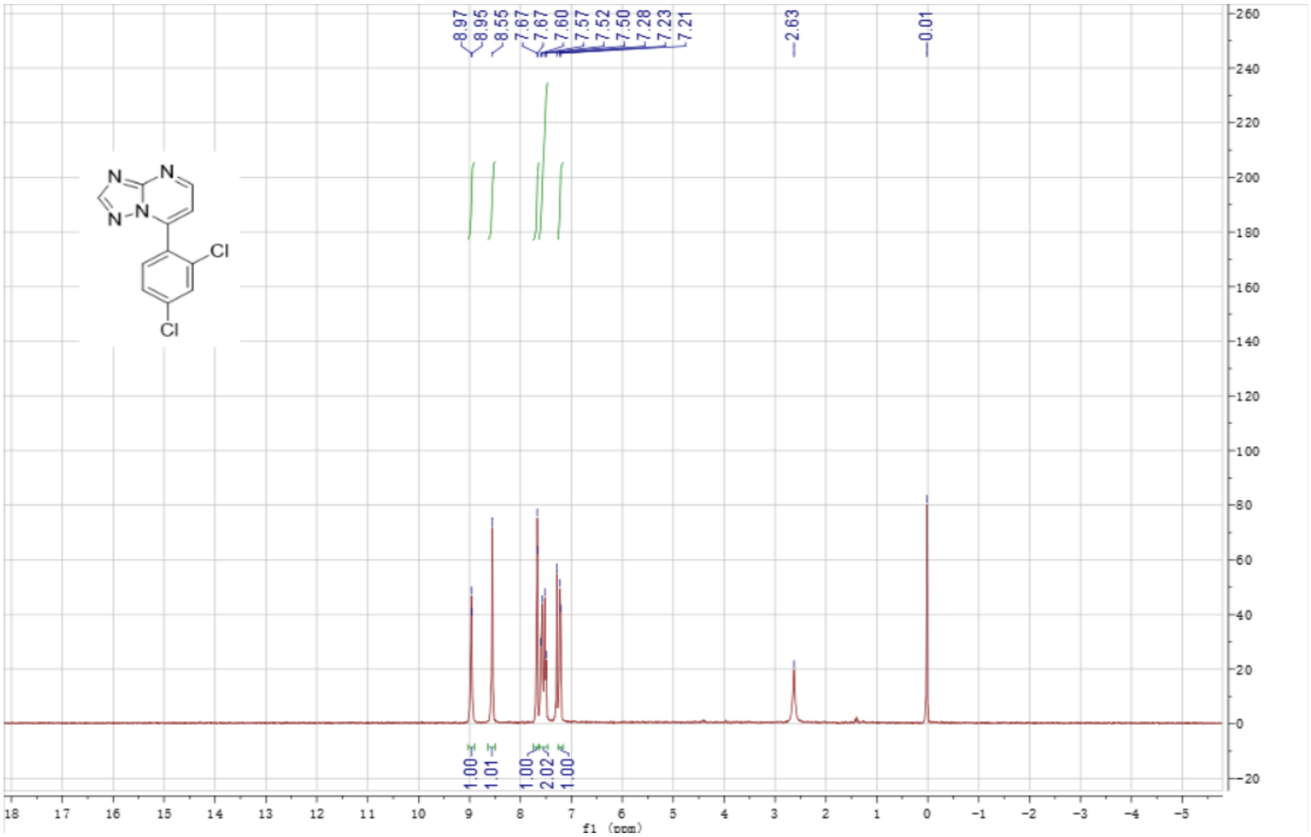

Figure 6 The H NMR spectrum of compound 3e

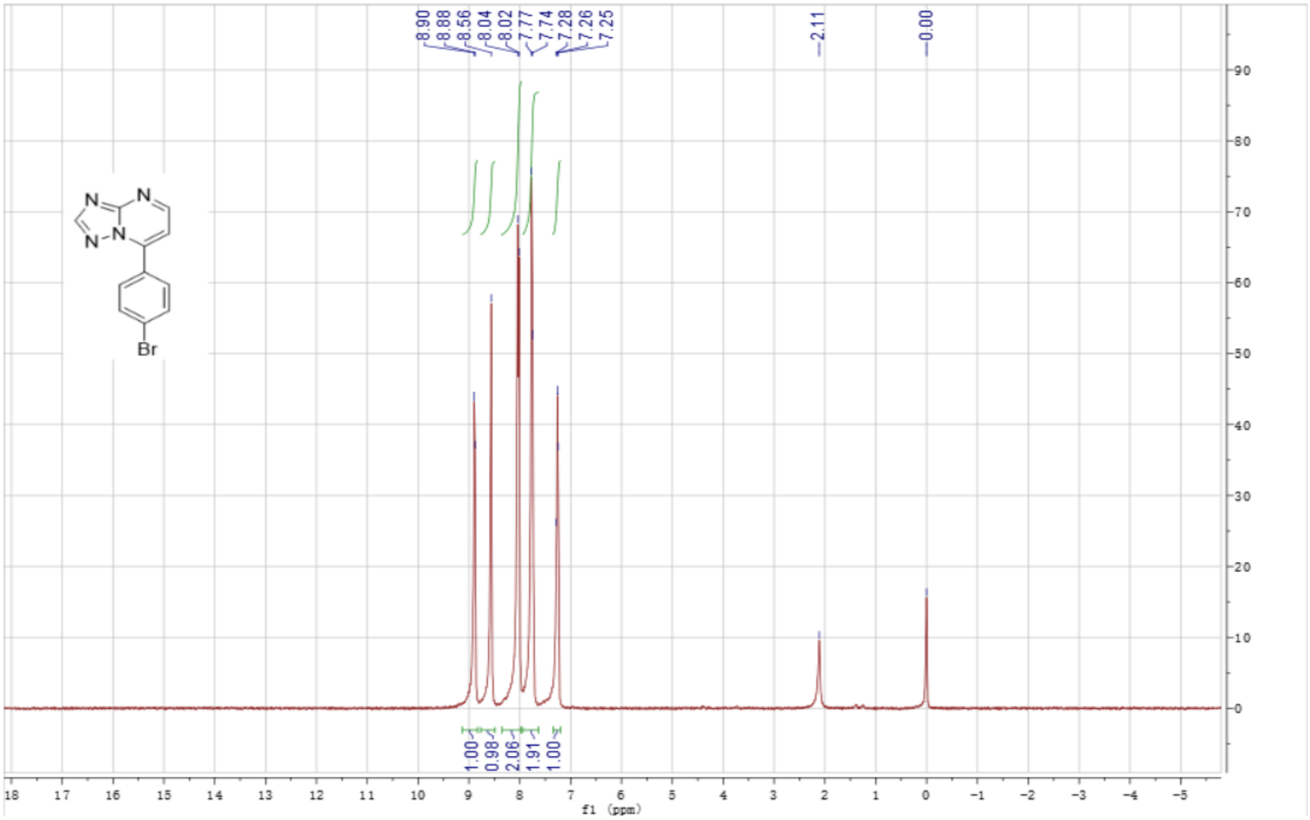

Figure 7 The H NMR spectrum of compound 3f

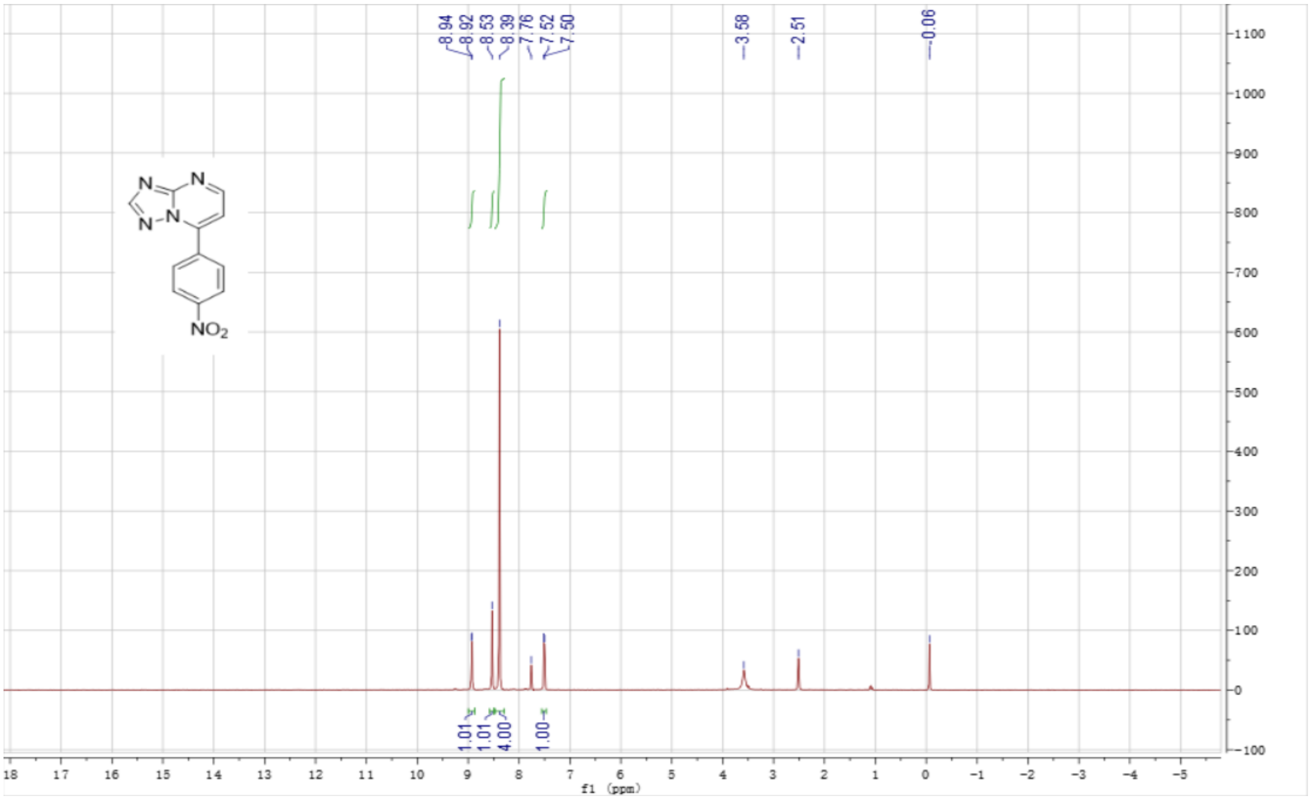

Figure 8 The H NMR spectrum of compound 3g

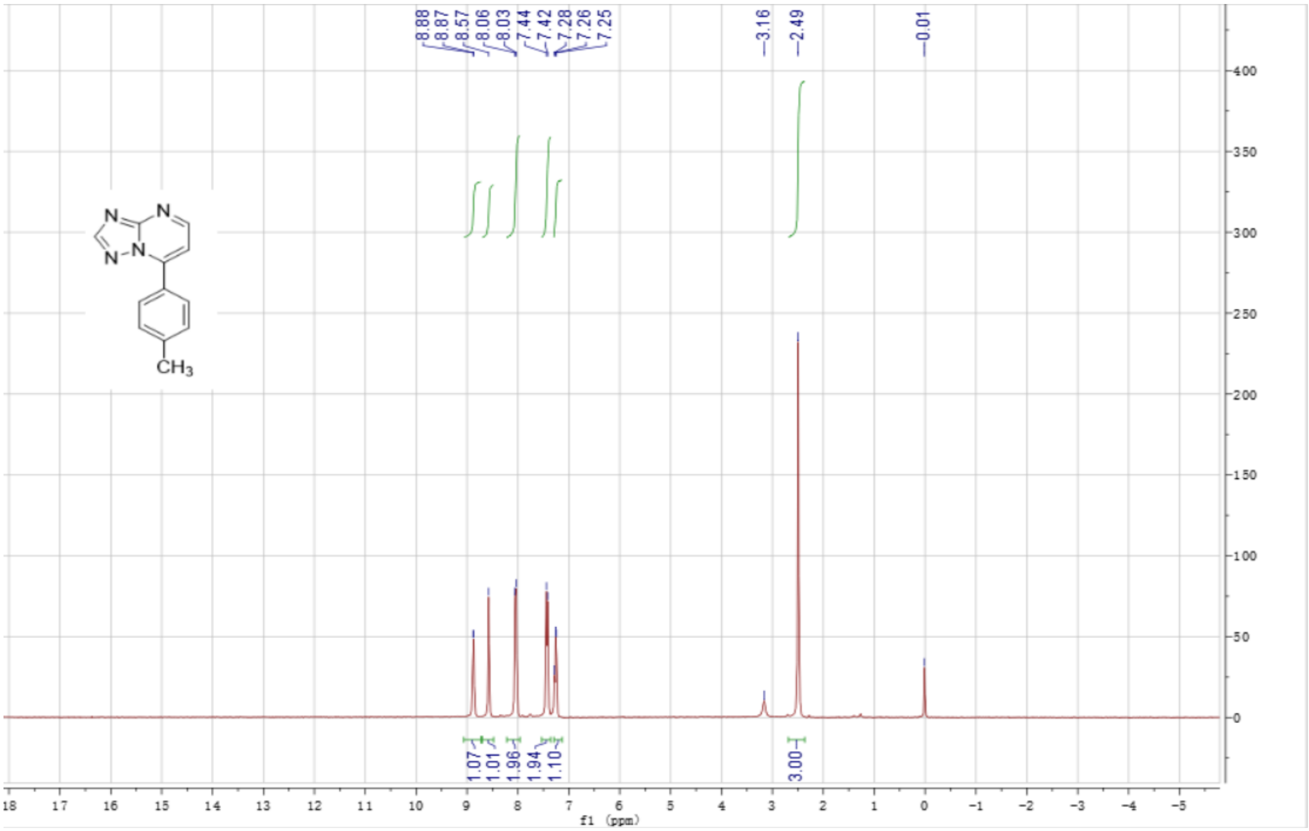


Figure 9 The H NMR spectrum of compound 3h

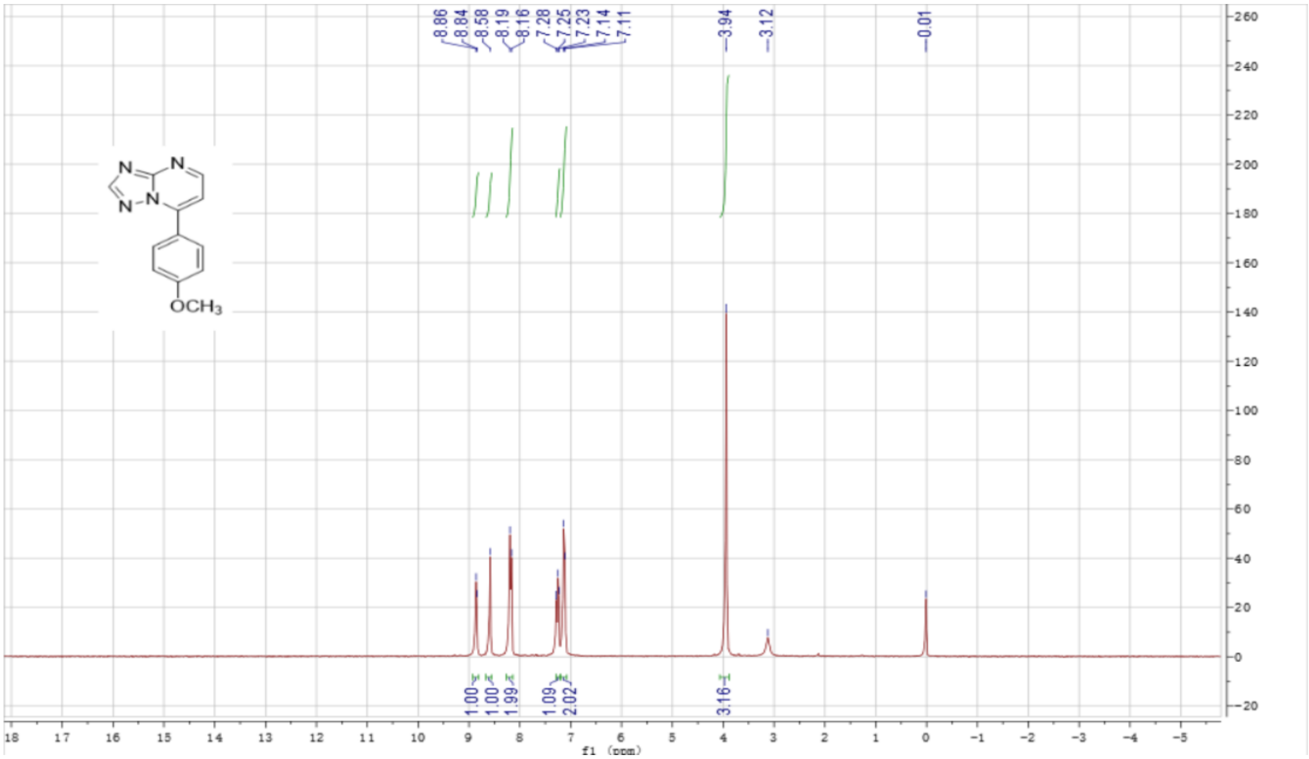


Figure 10 The H NMR spectrum of compound 3i

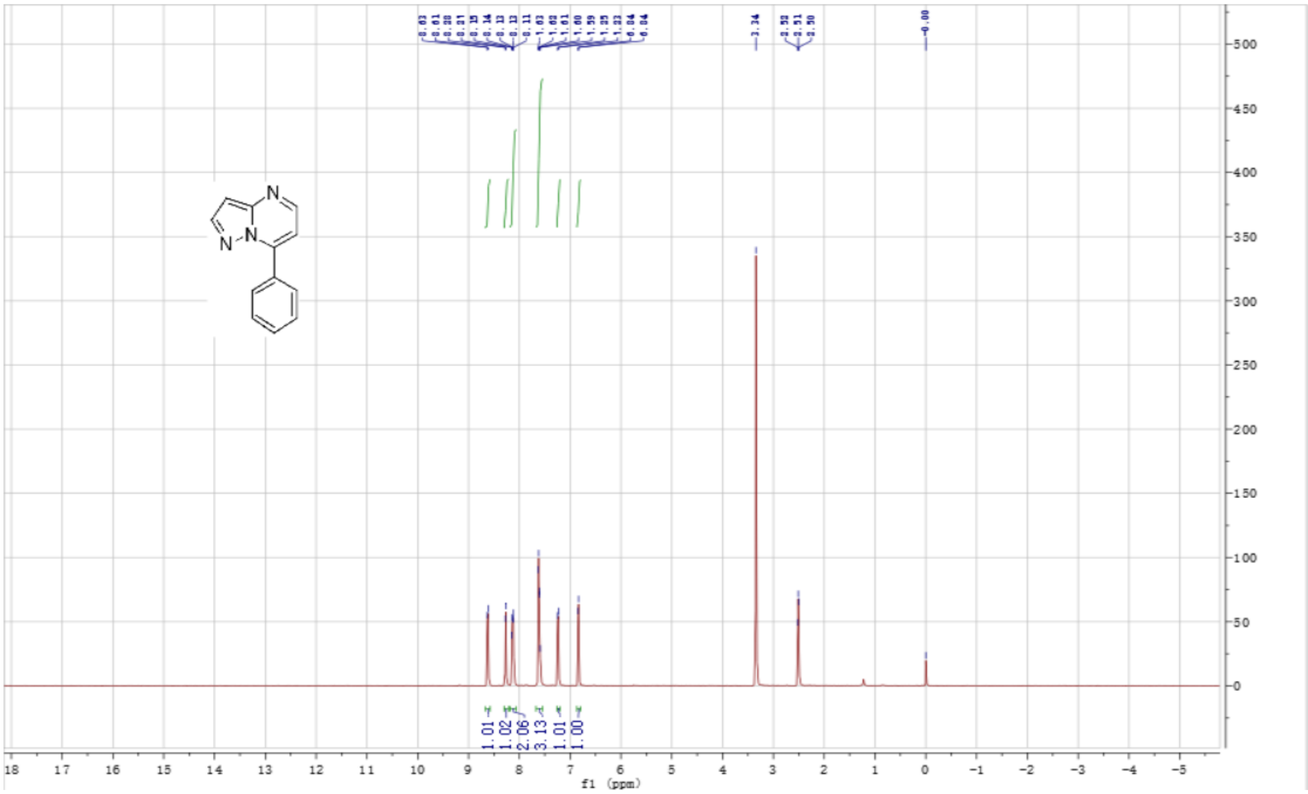


Figure 11 The H NMR spectrum of compound 4a

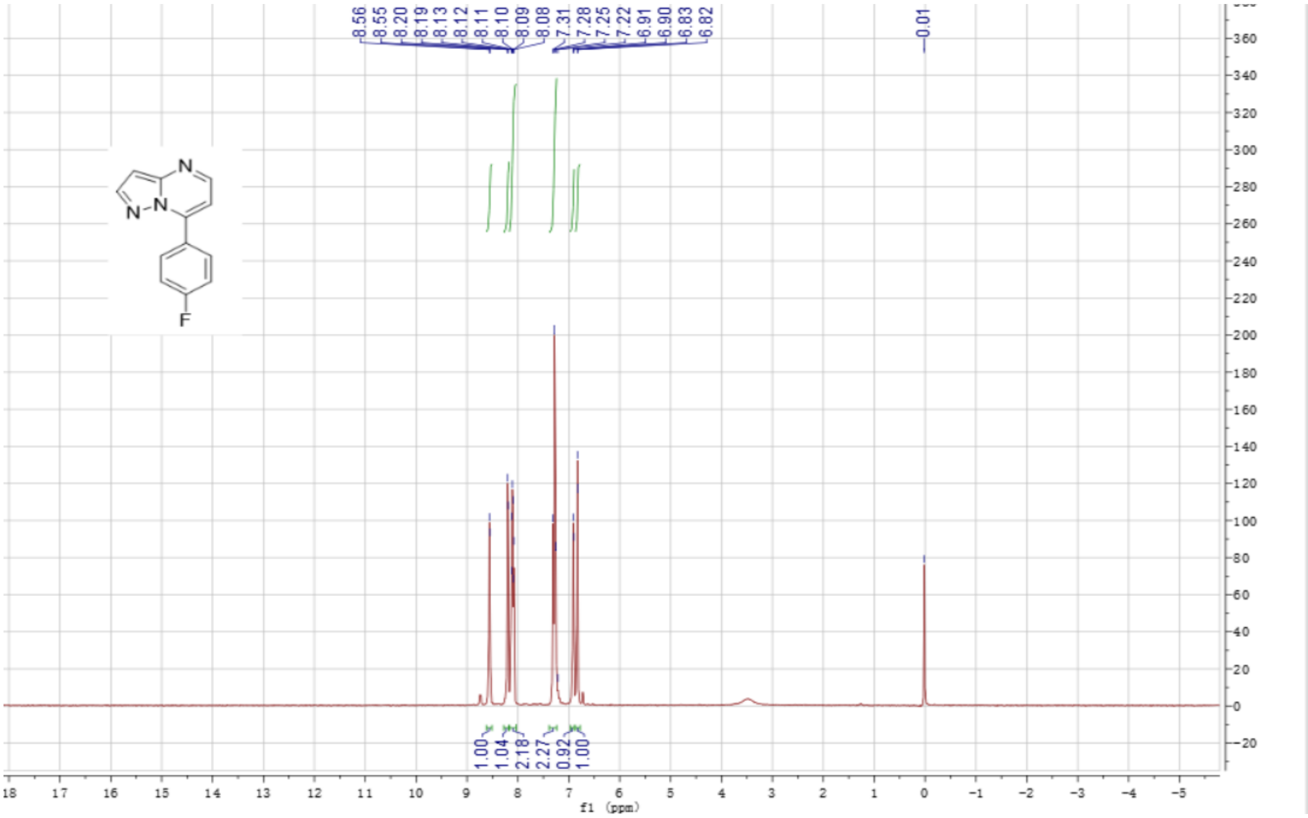


Figure 12 The H NMR spectrum of compound 4b

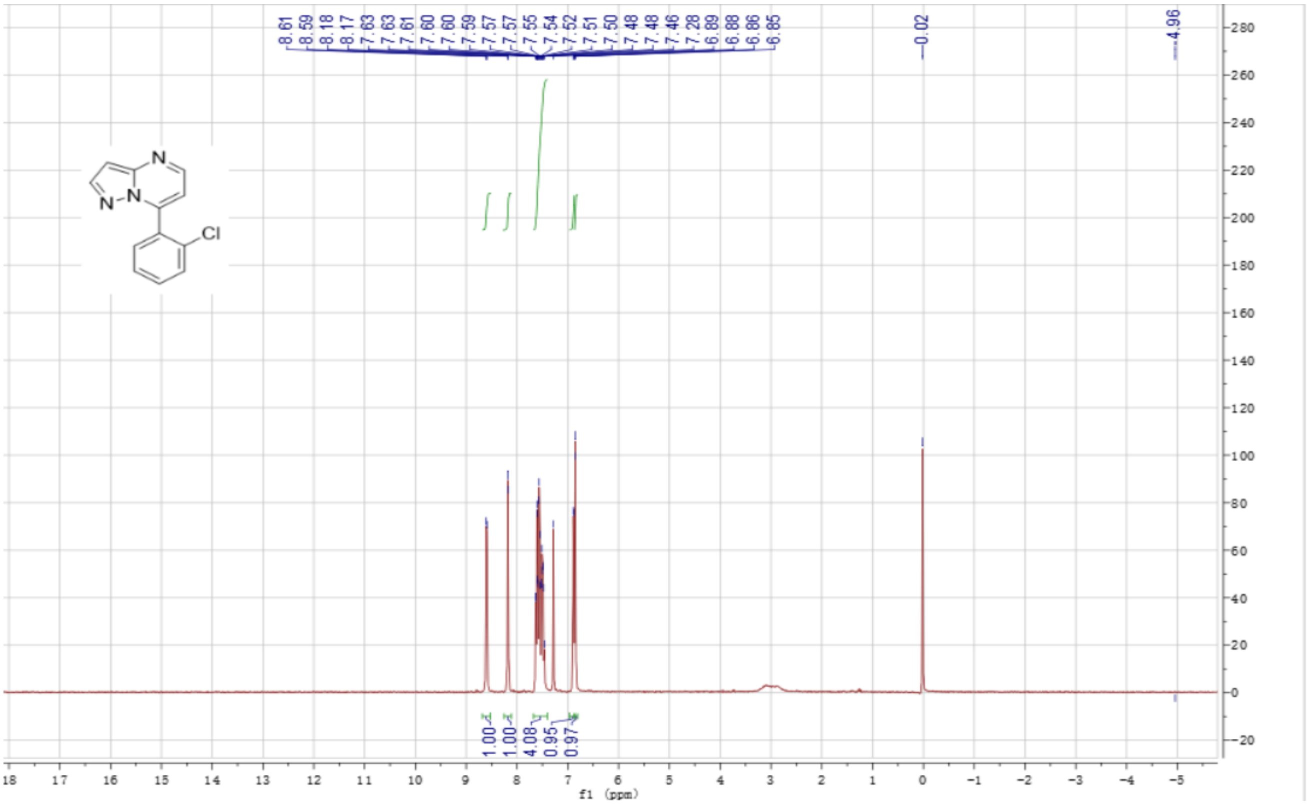


Figure 13 The H NMR spectrum of compound 4c

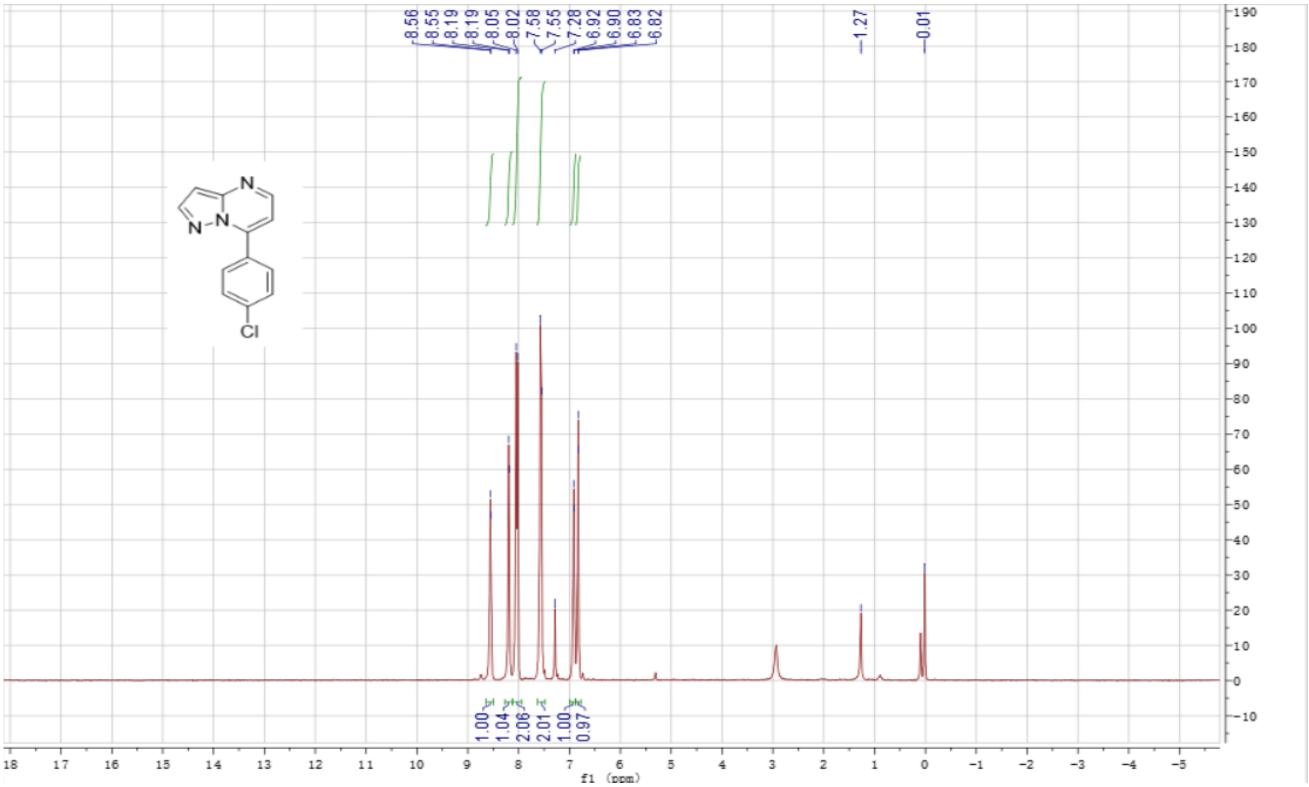


Figure 14 The H NMR spectrum of compound 4d

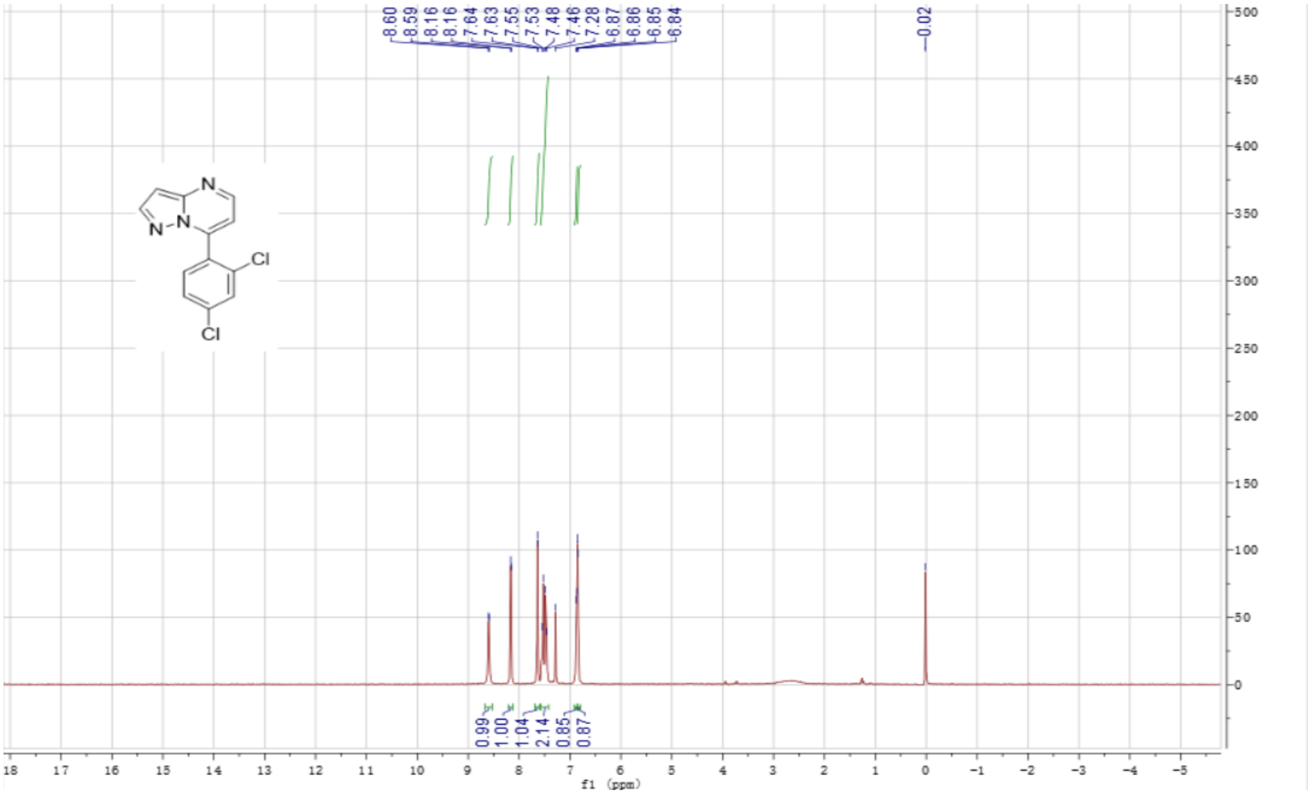

Figure 15 The H NMR spectrum of compound 4e

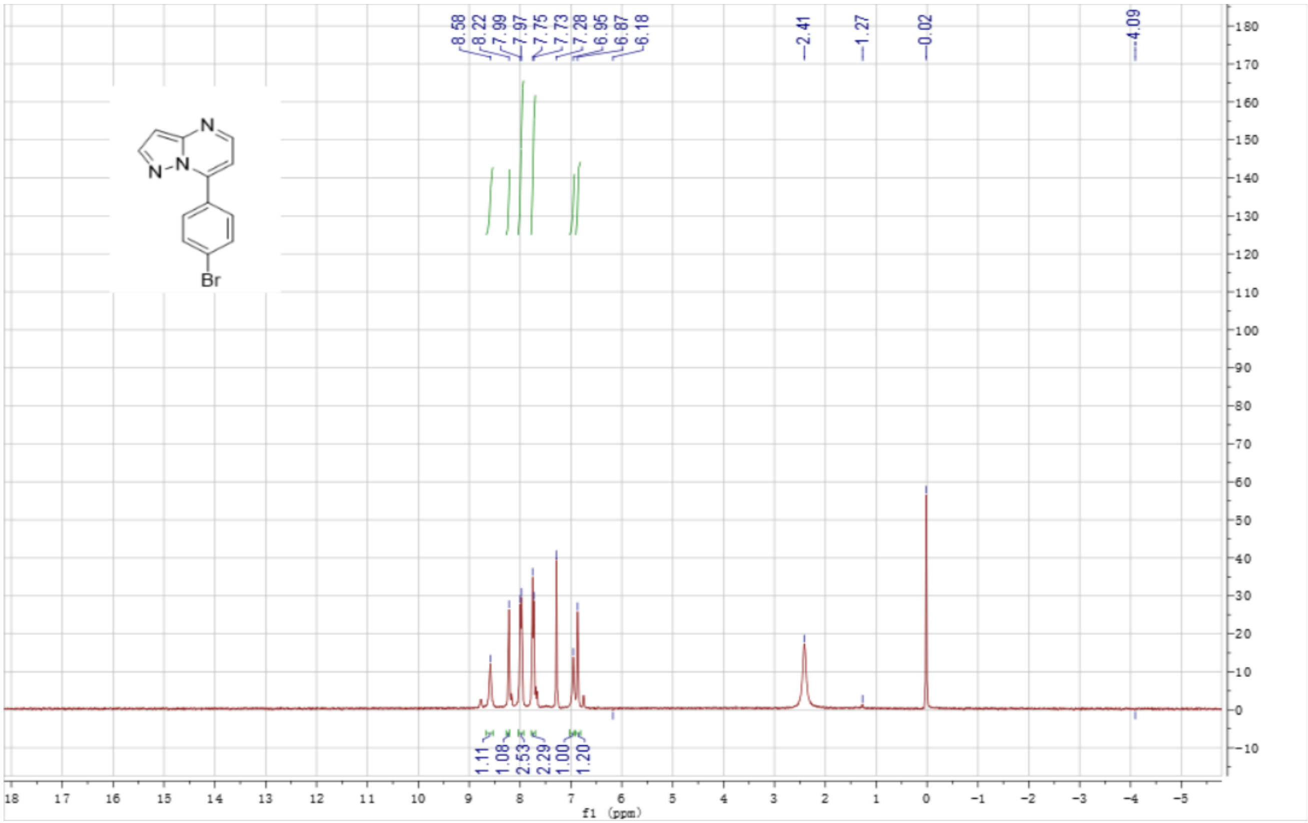

Figure 16 The H NMR spectrum of compound 4f

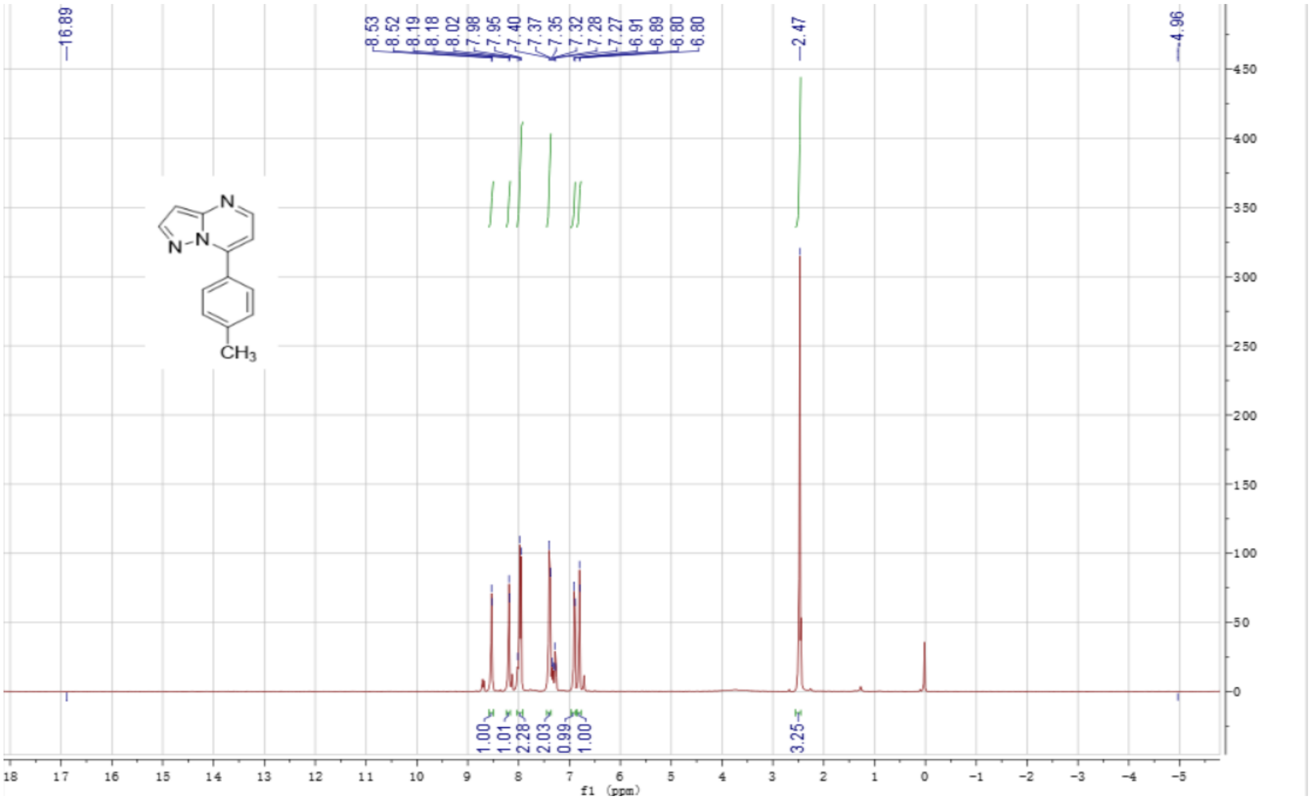

Figure 17 The H NMR spectrum of compound 4h

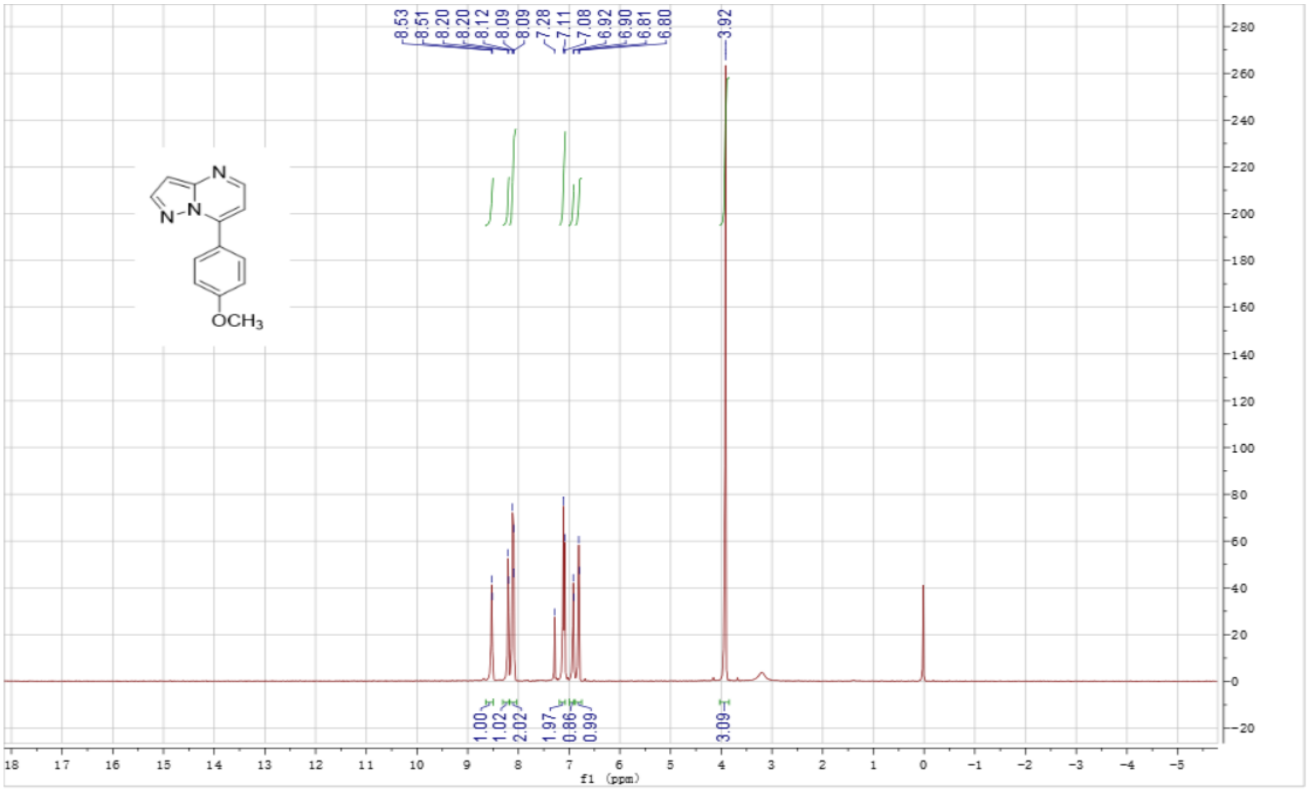

Figure 18 The H NMR spectrum of compound 4i

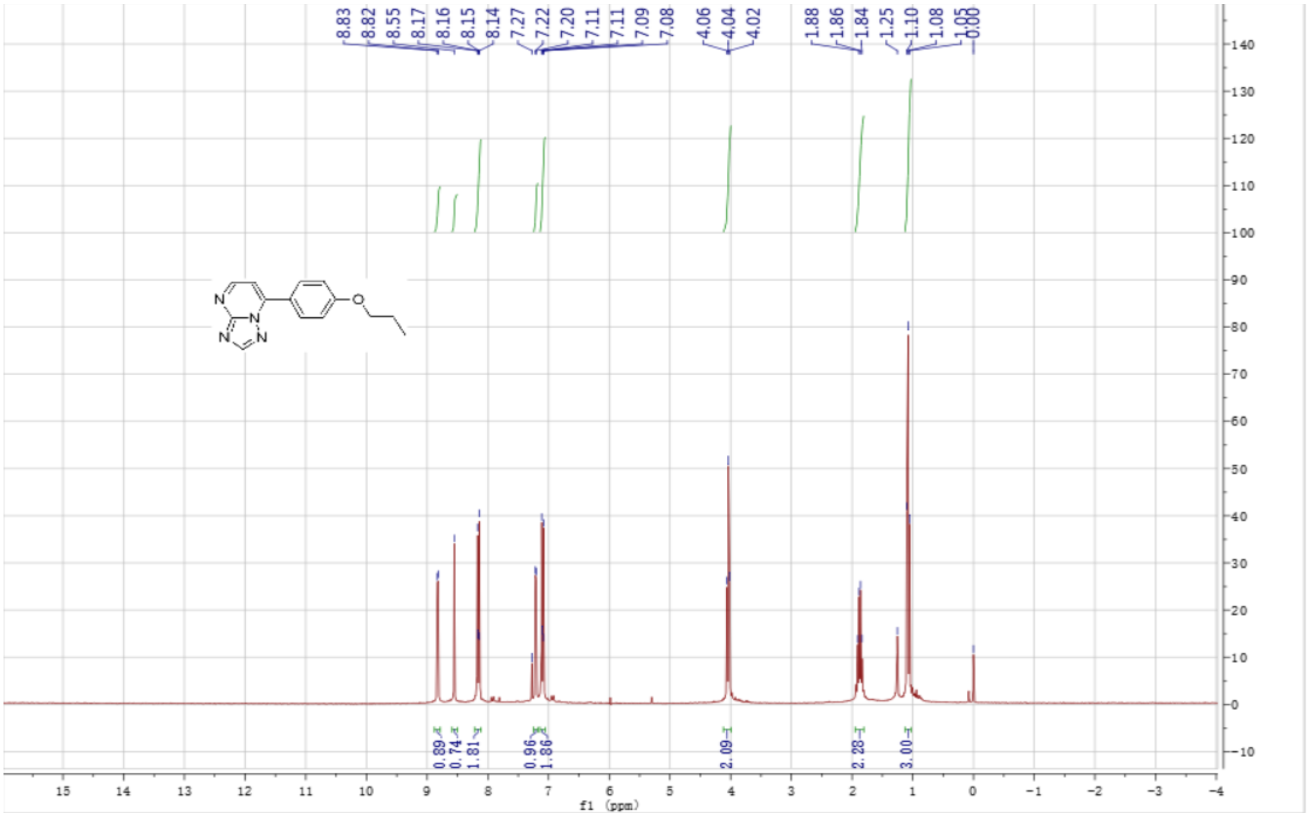

Figure 19 The H NMR spectrum of compound 6a

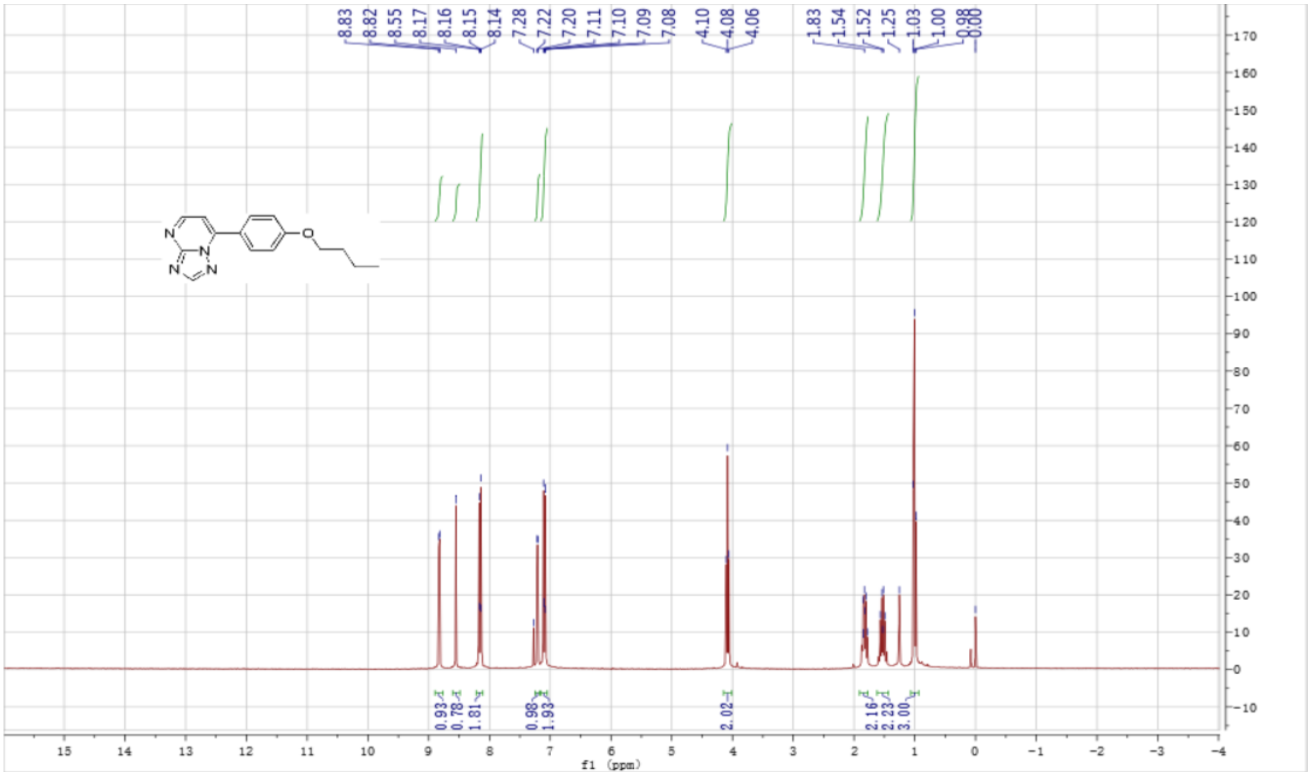

Figure 20 The H NMR spectrum of compound 6b

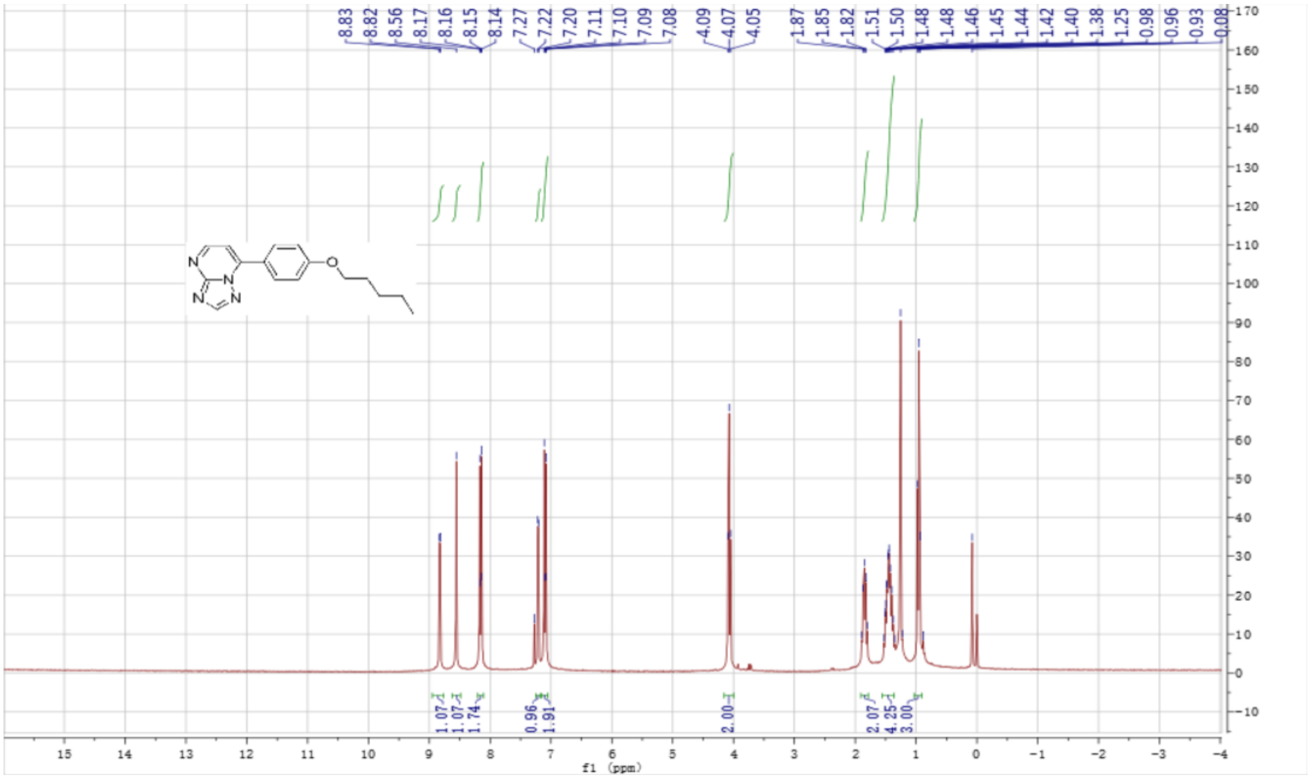


Figure 21 The H NMR spectrum of compound 6c

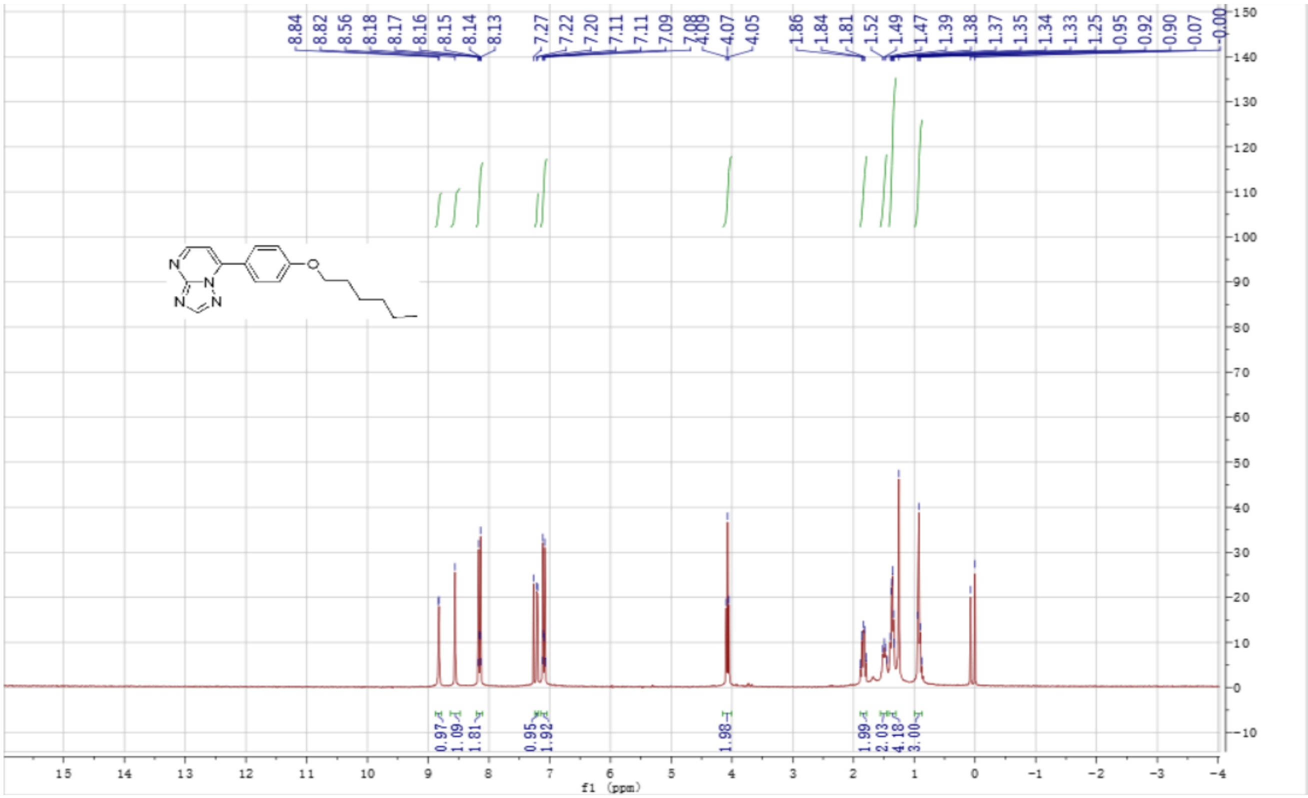


Figure 22 The H NMR spectrum of compound 6d

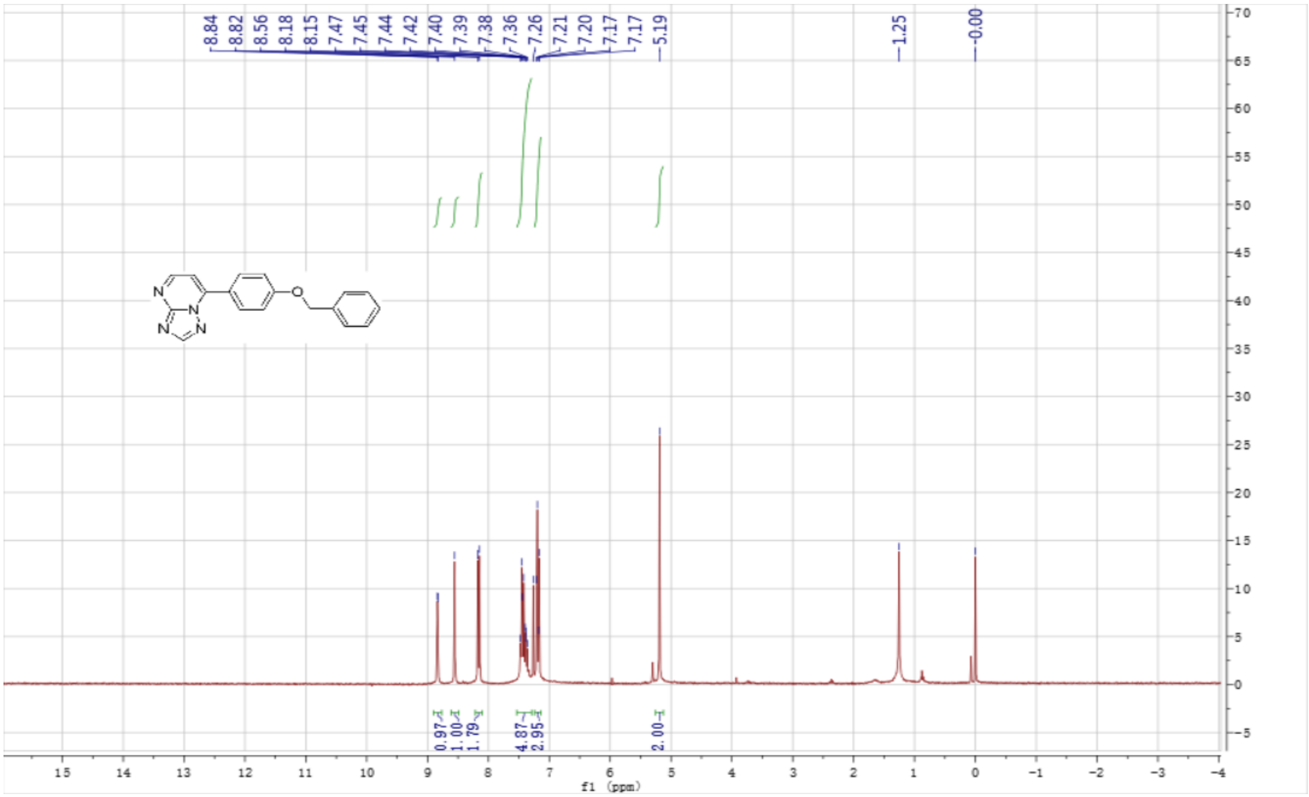

Figure 23 The H NMR spectrum of compound 6e

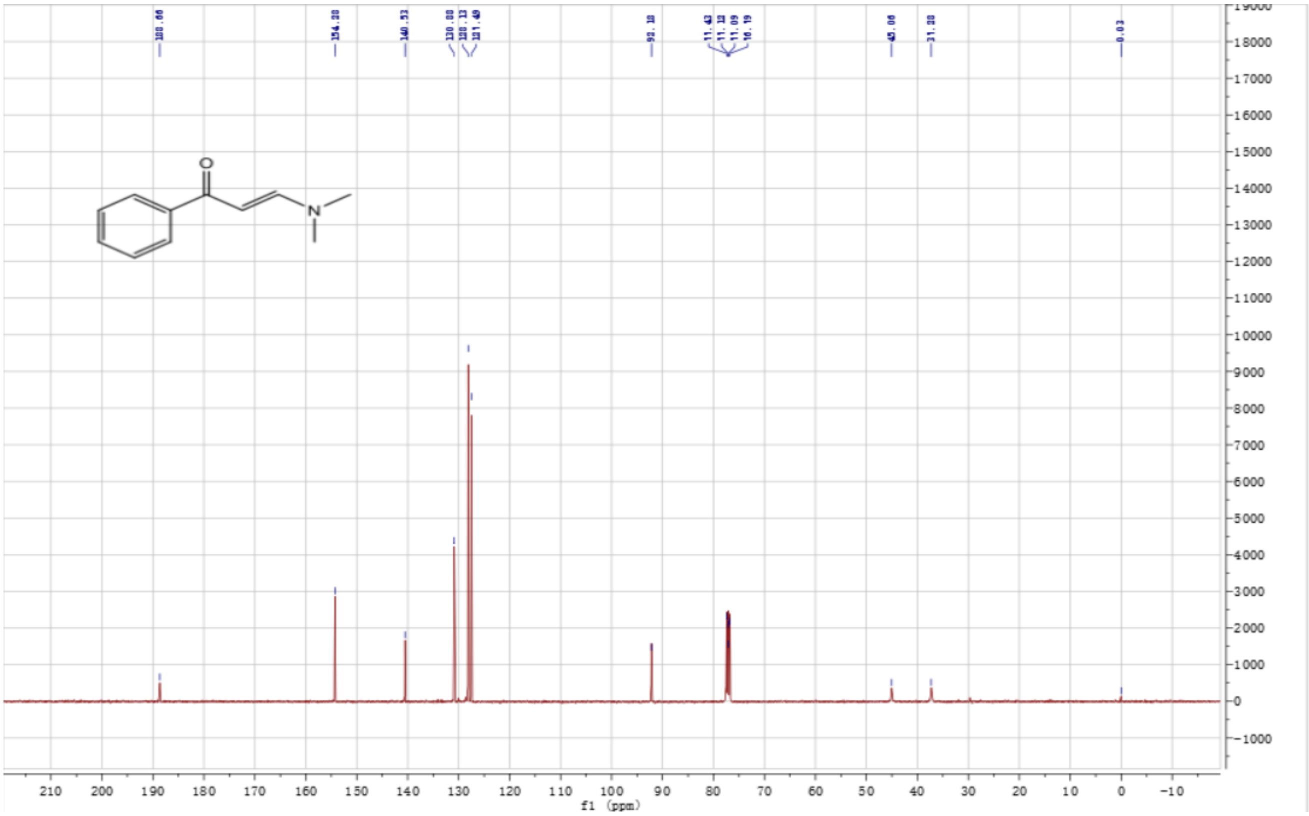

Figure 24 The C NMR spectrum of compound 2a

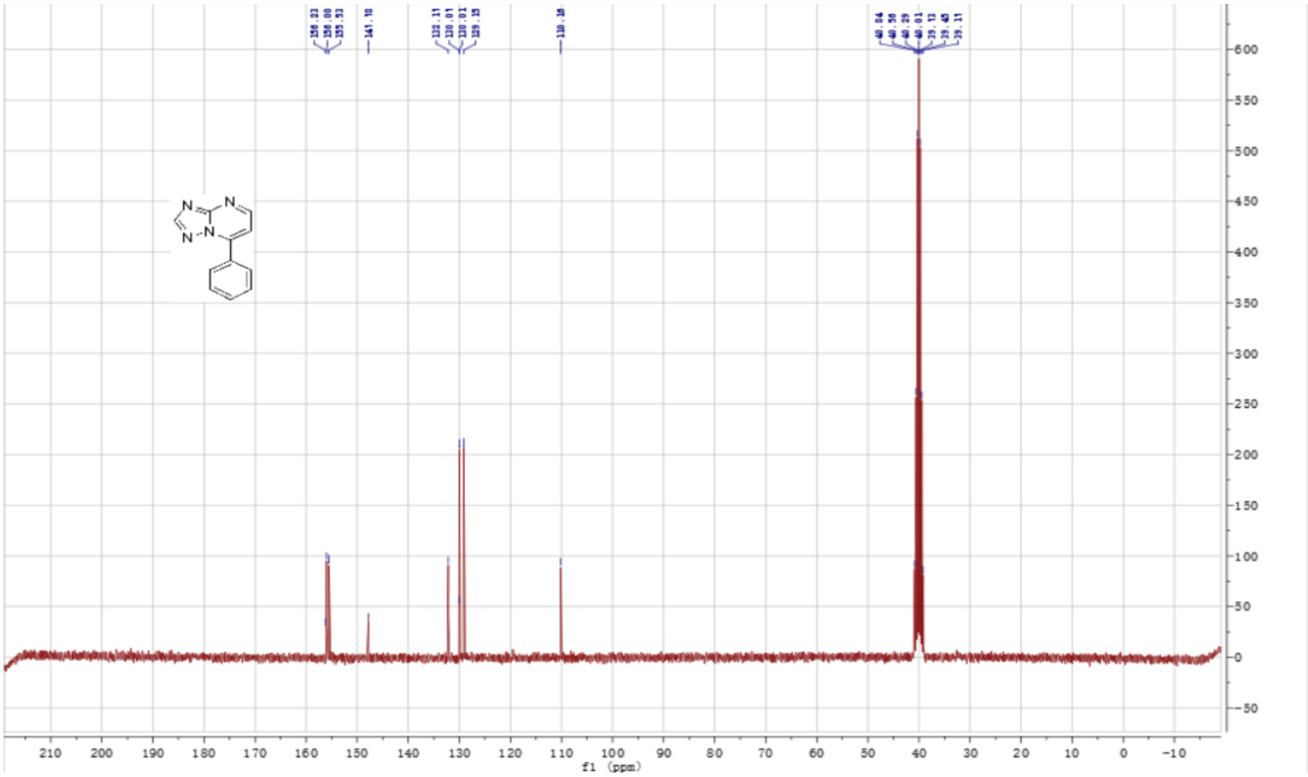

Figure 25 The C NMR spectrum of compound 3a

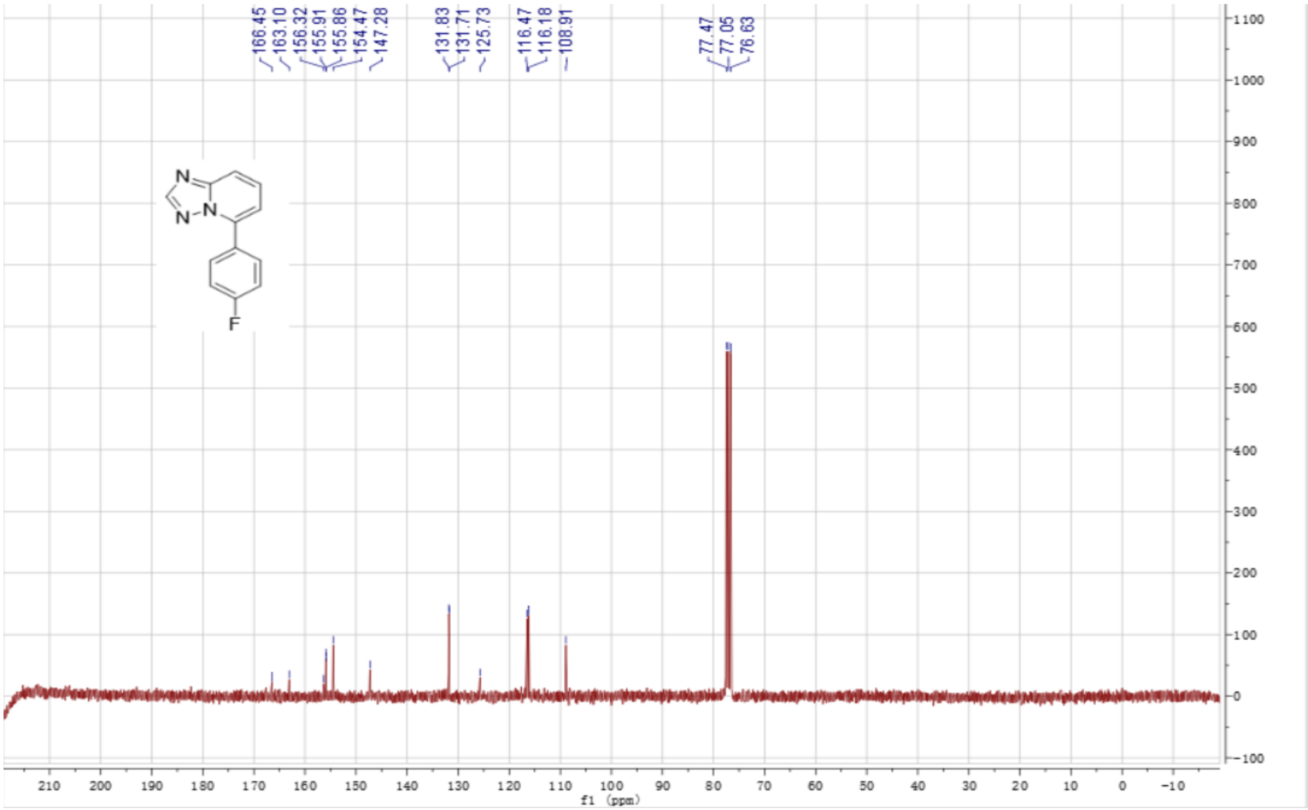


Figure 26 The C NMR spectrum of compound 3b

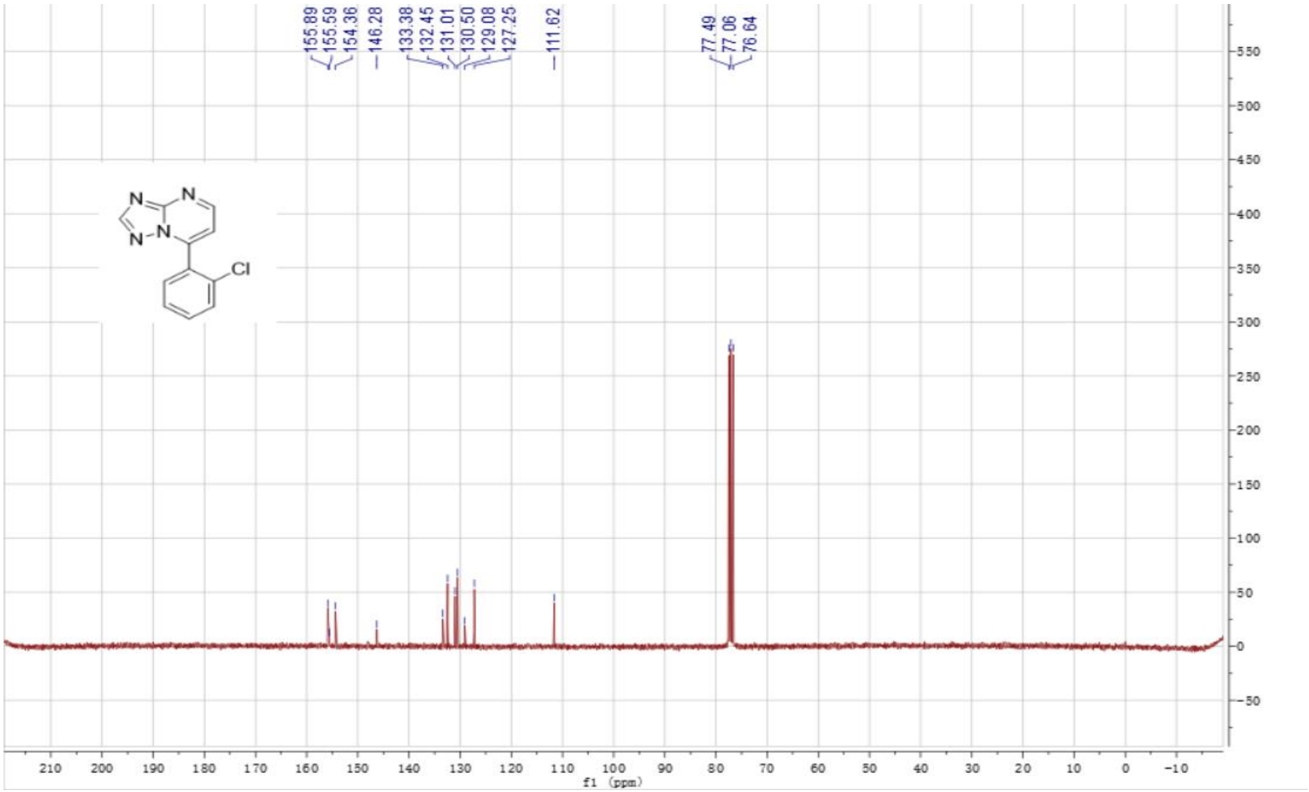


Figure 27 The C NMR spectrum of compound 3c

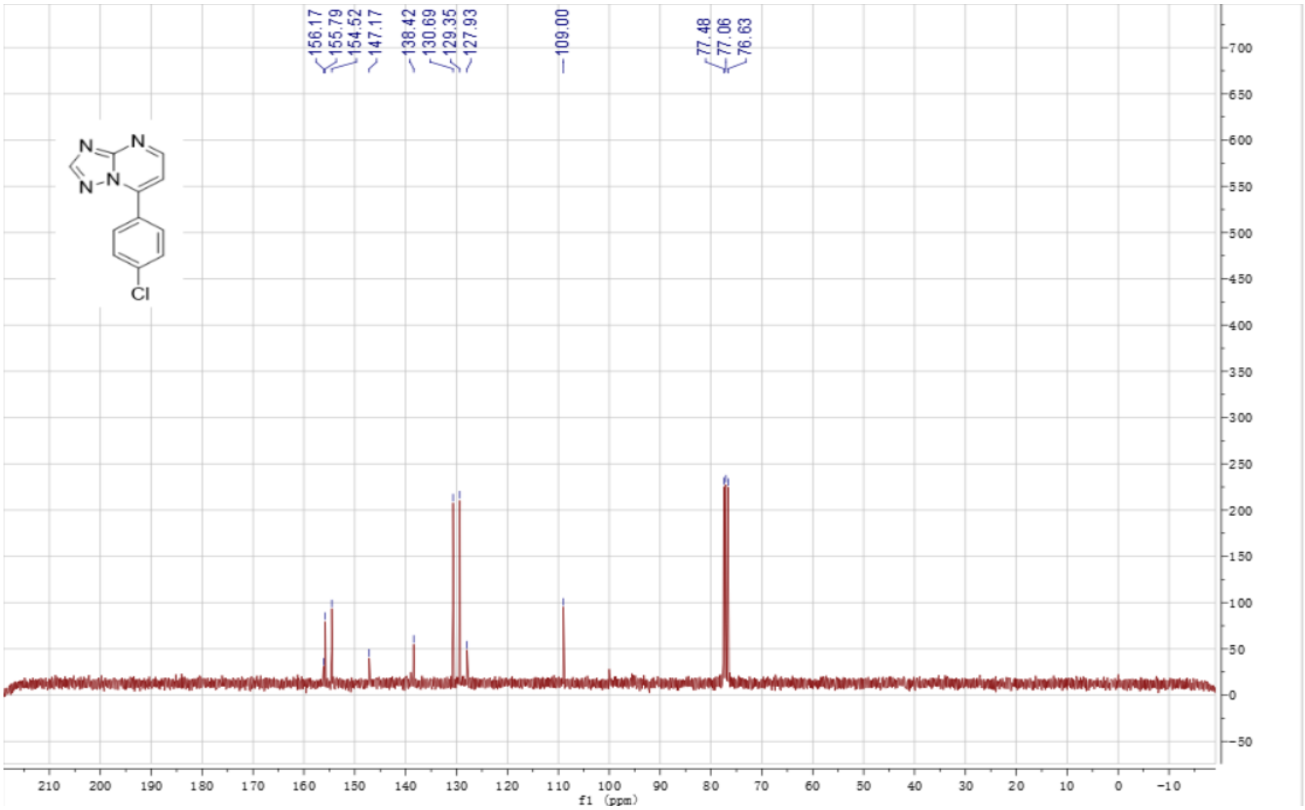


Figure 28 The C NMR spectrum of compound 3d

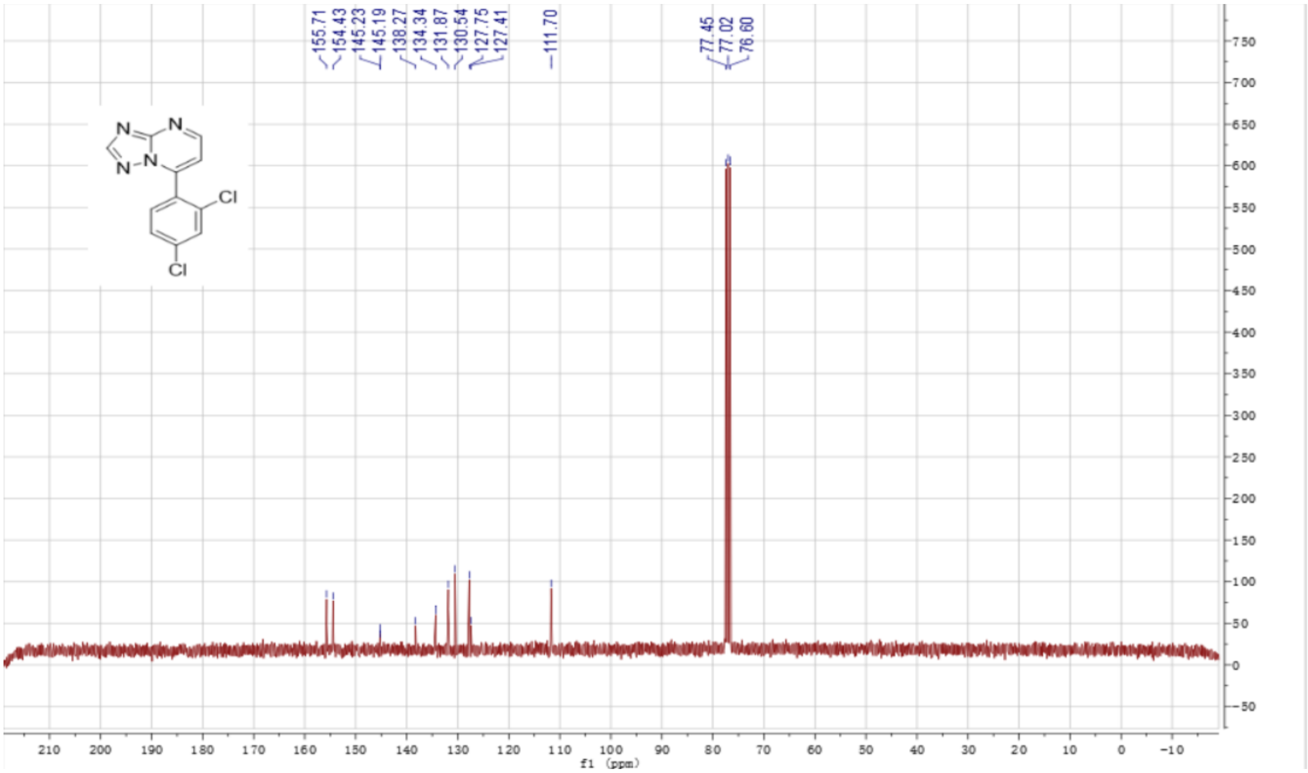


Figure 29 The C NMR spectrum of compound 3e

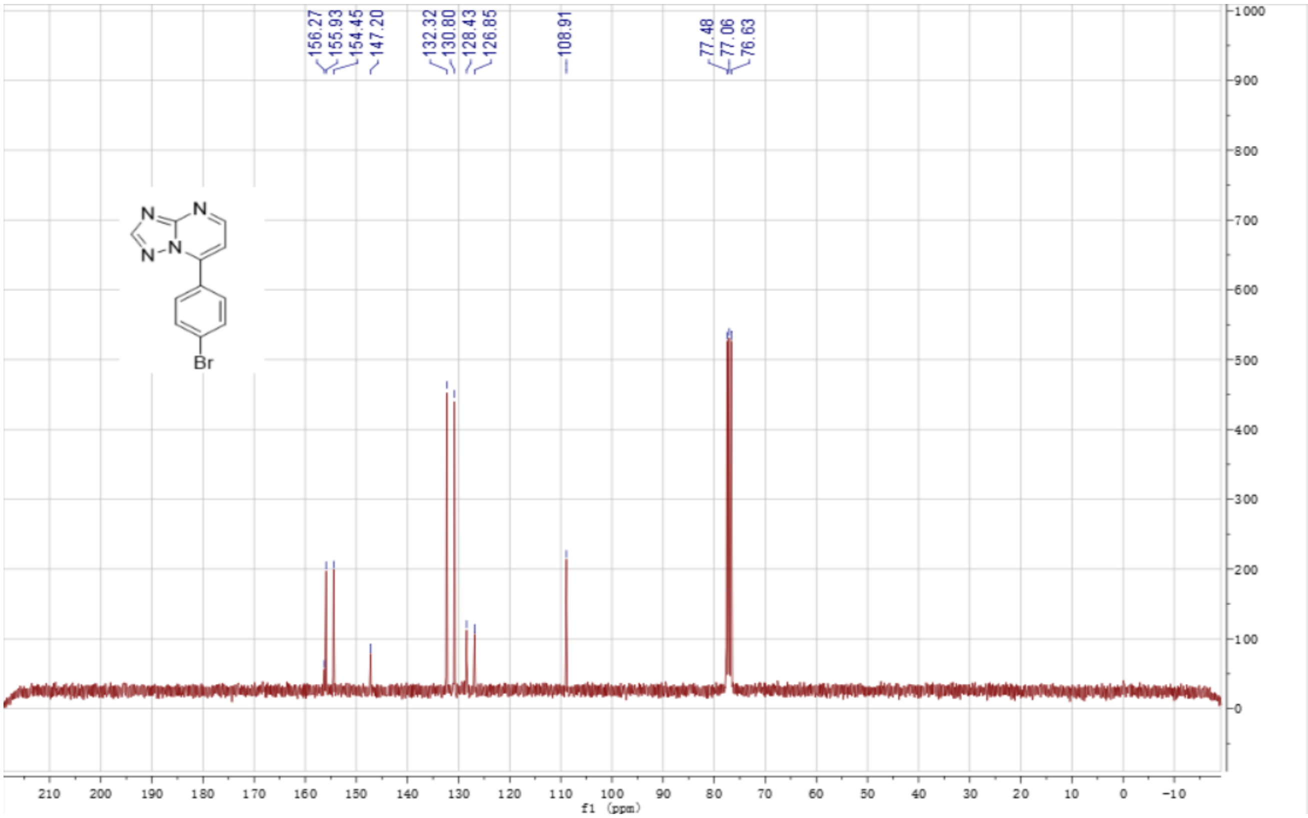


Figure 30 The C NMR spectrum of compound 3f

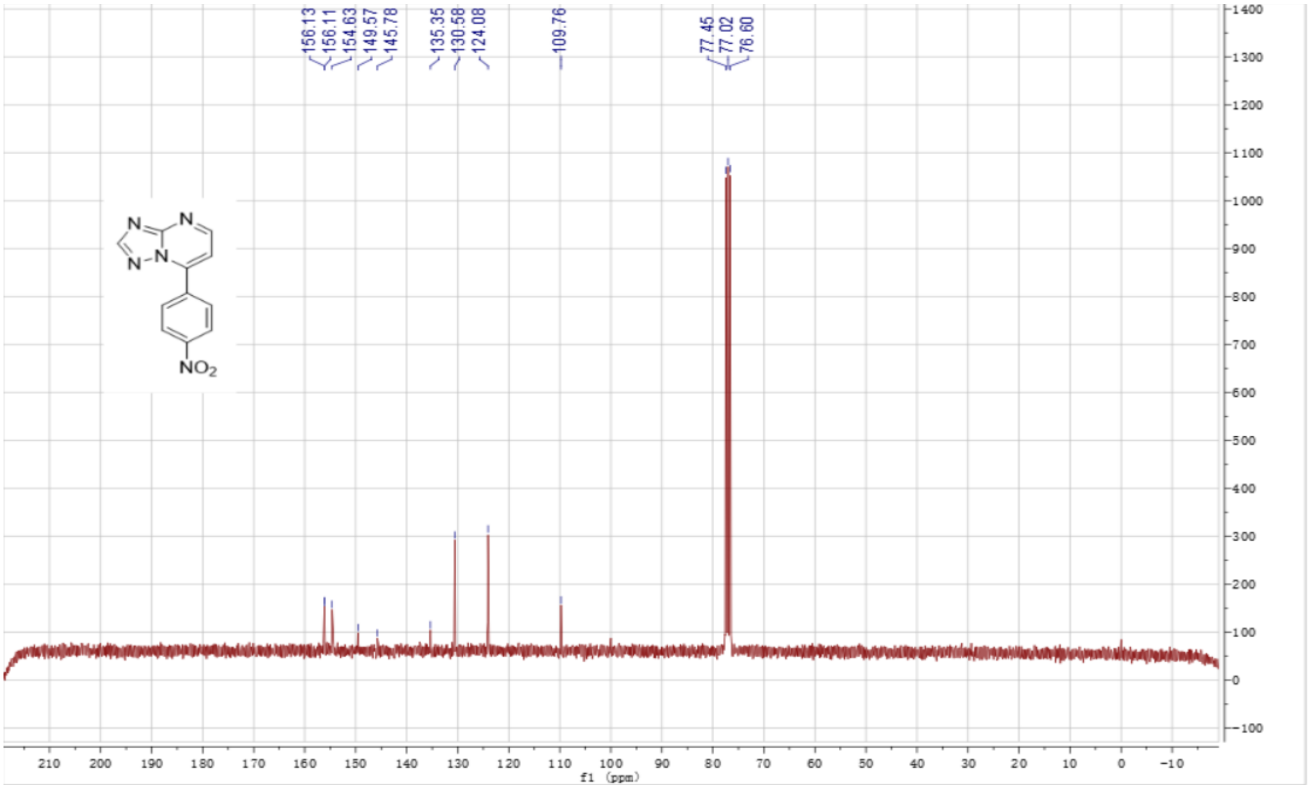


Figure 31 The C NMR spectrum of compound 3g

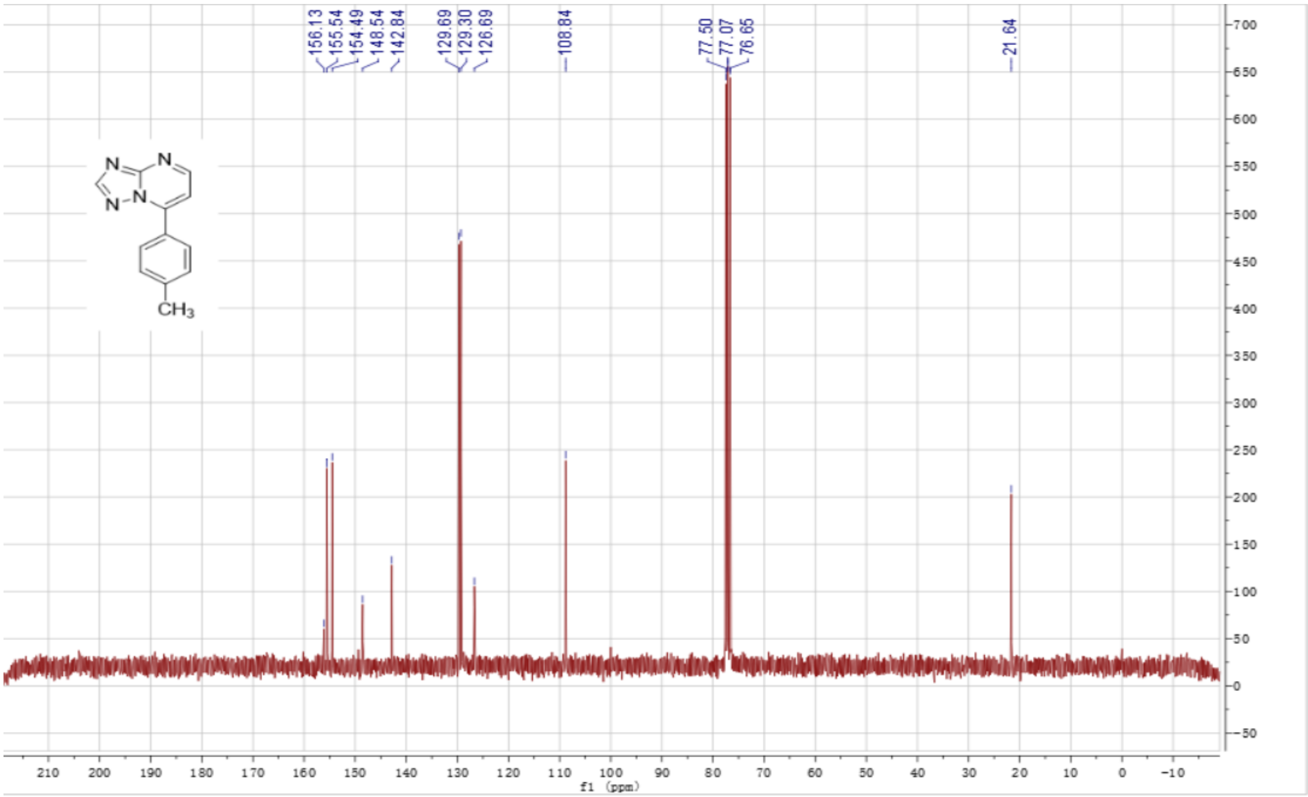


Figure 32 The C NMR spectrum of compound 3h

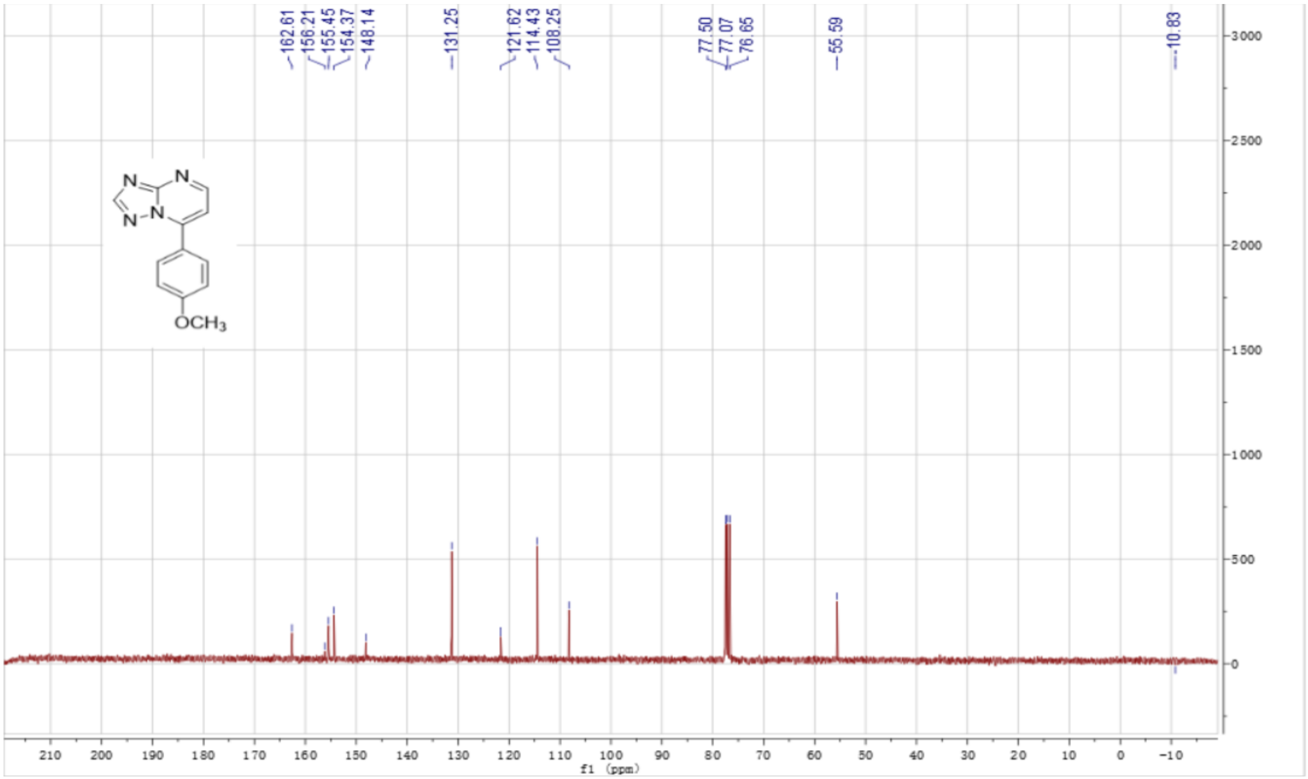


Figure 33 The C NMR spectrum of compound 3i

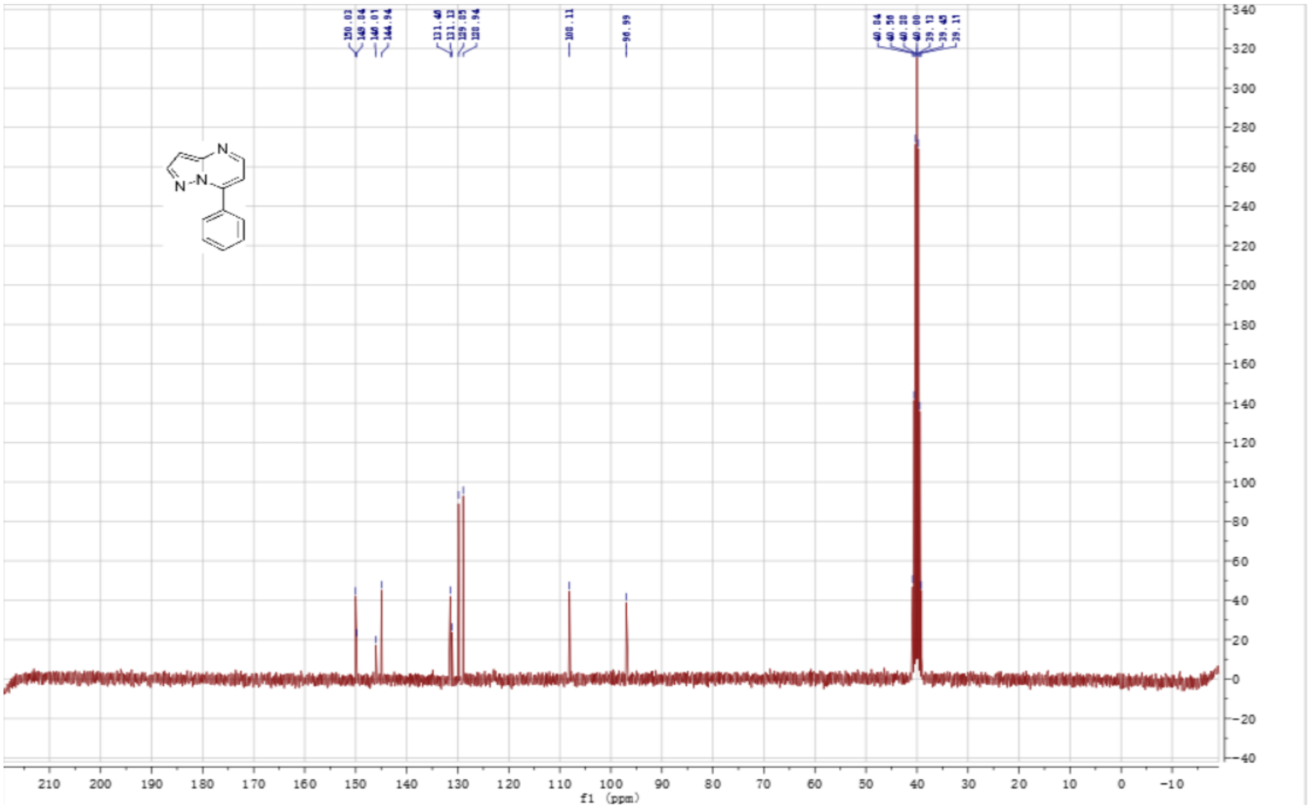


Figure 34 The C NMR spectrum of compound 4a

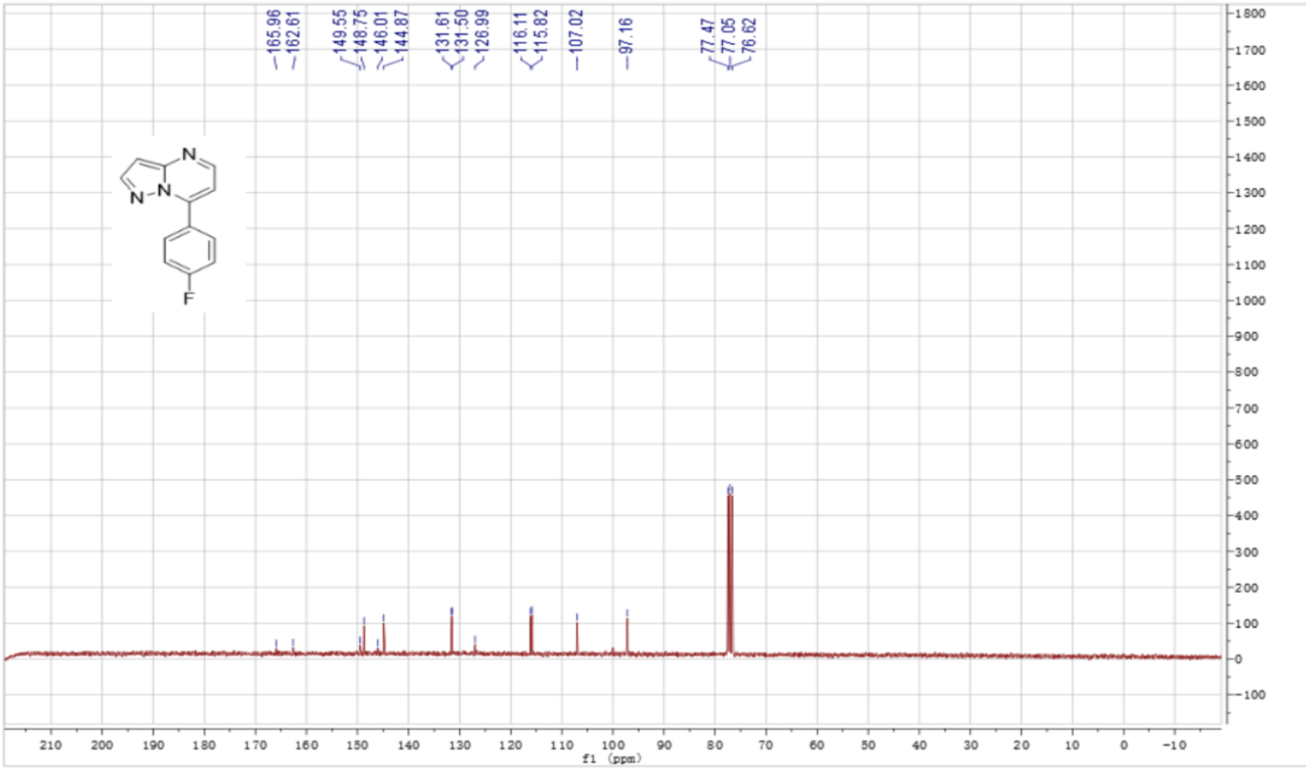

Figure 35 The C NMR spectrum of compound 4b

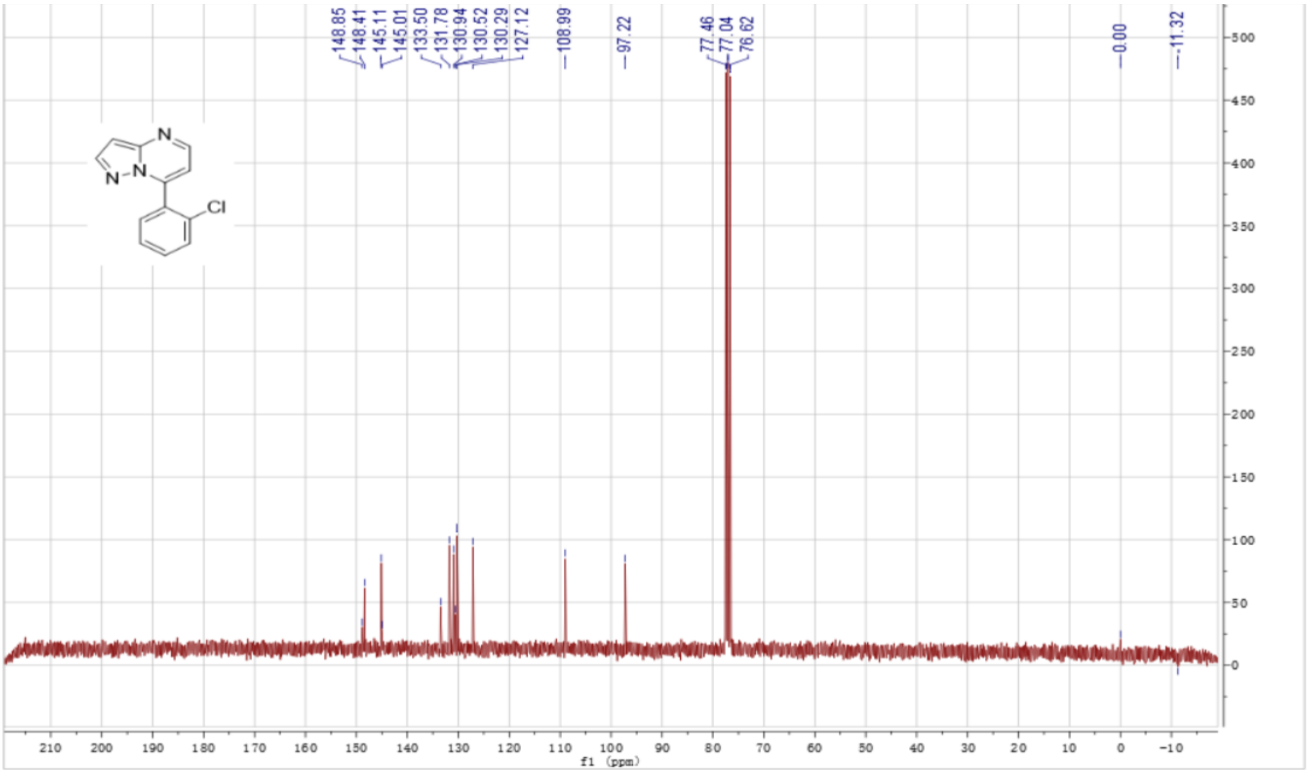

Figure 36 The C NMR spectrum of compound 4c

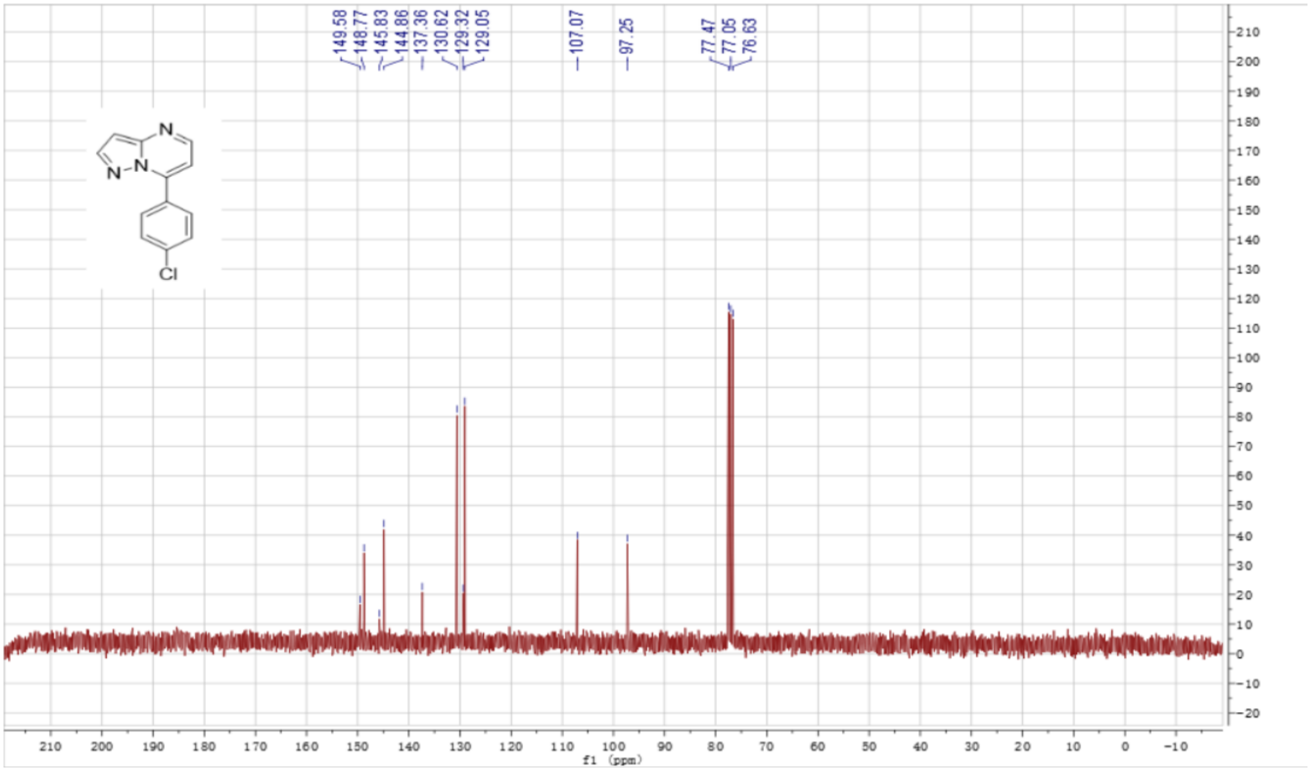


Figure 37 The C NMR spectrum of compound 4d

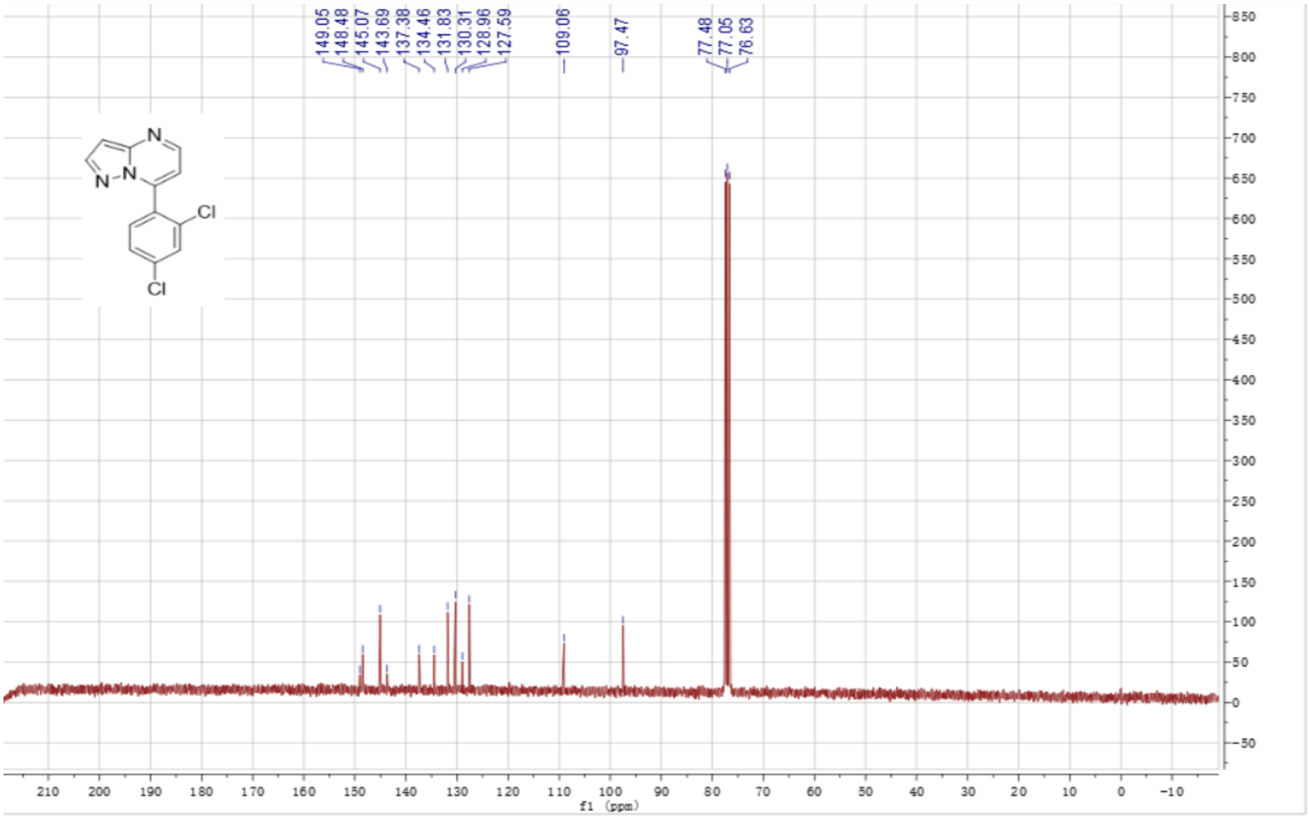


Figure 38 The C NMR spectrum of compound 4e

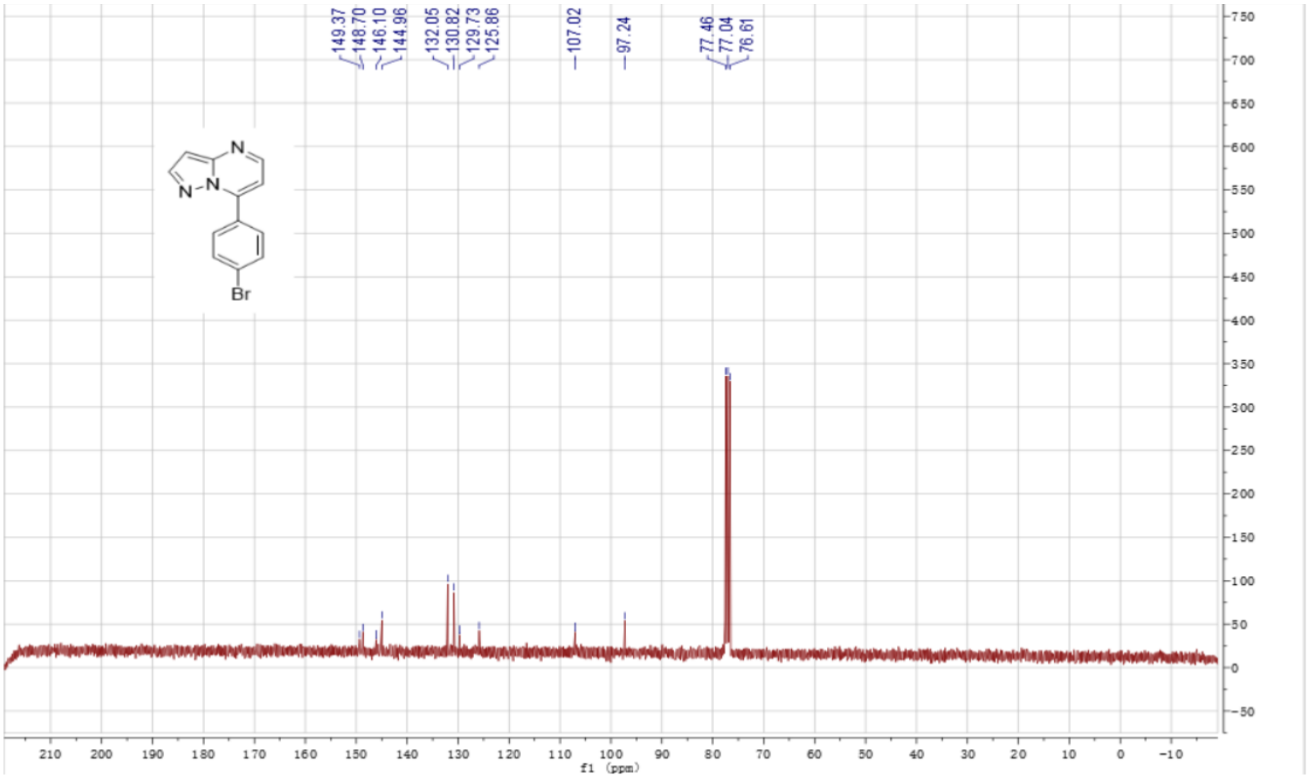


Figure 39 The C NMR spectrum of compound 4f

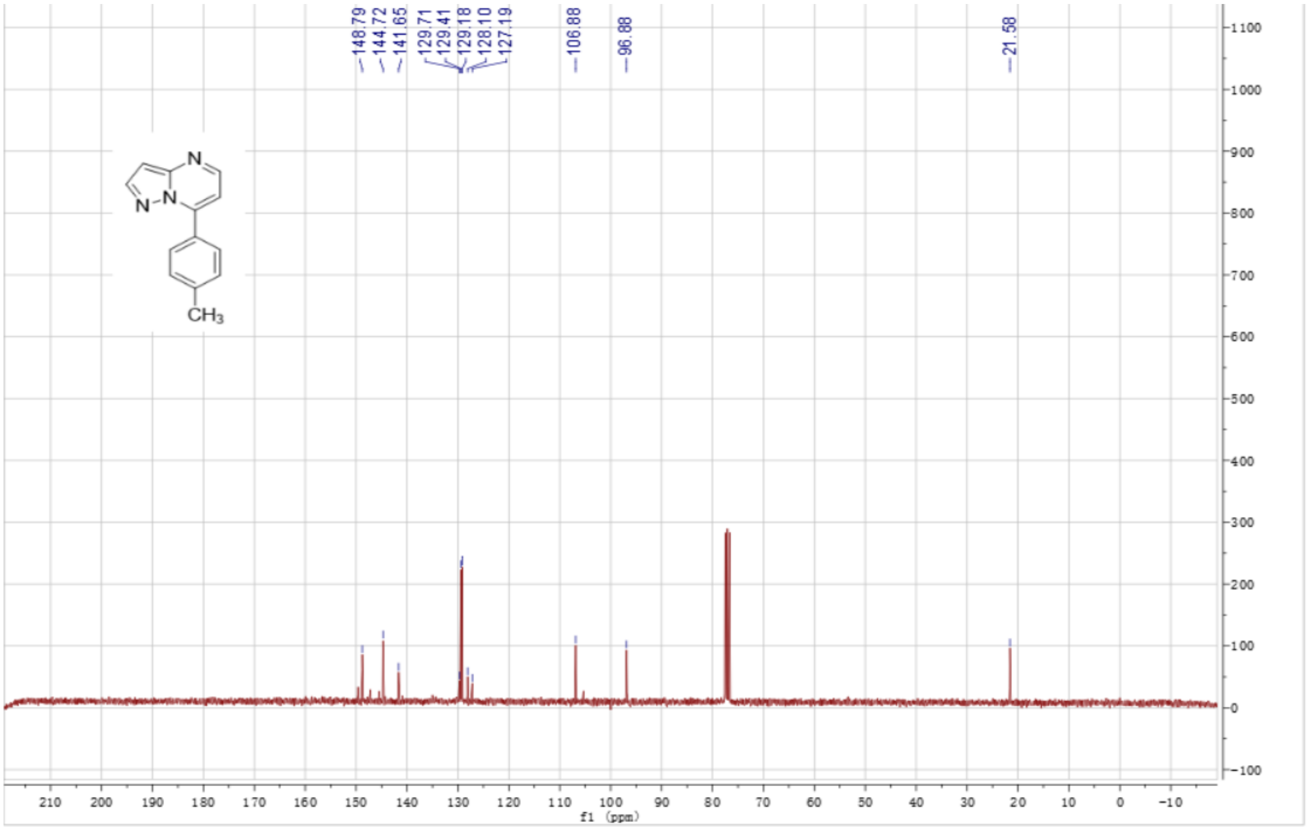


Figure 40 The C NMR spectrum of compound 4h

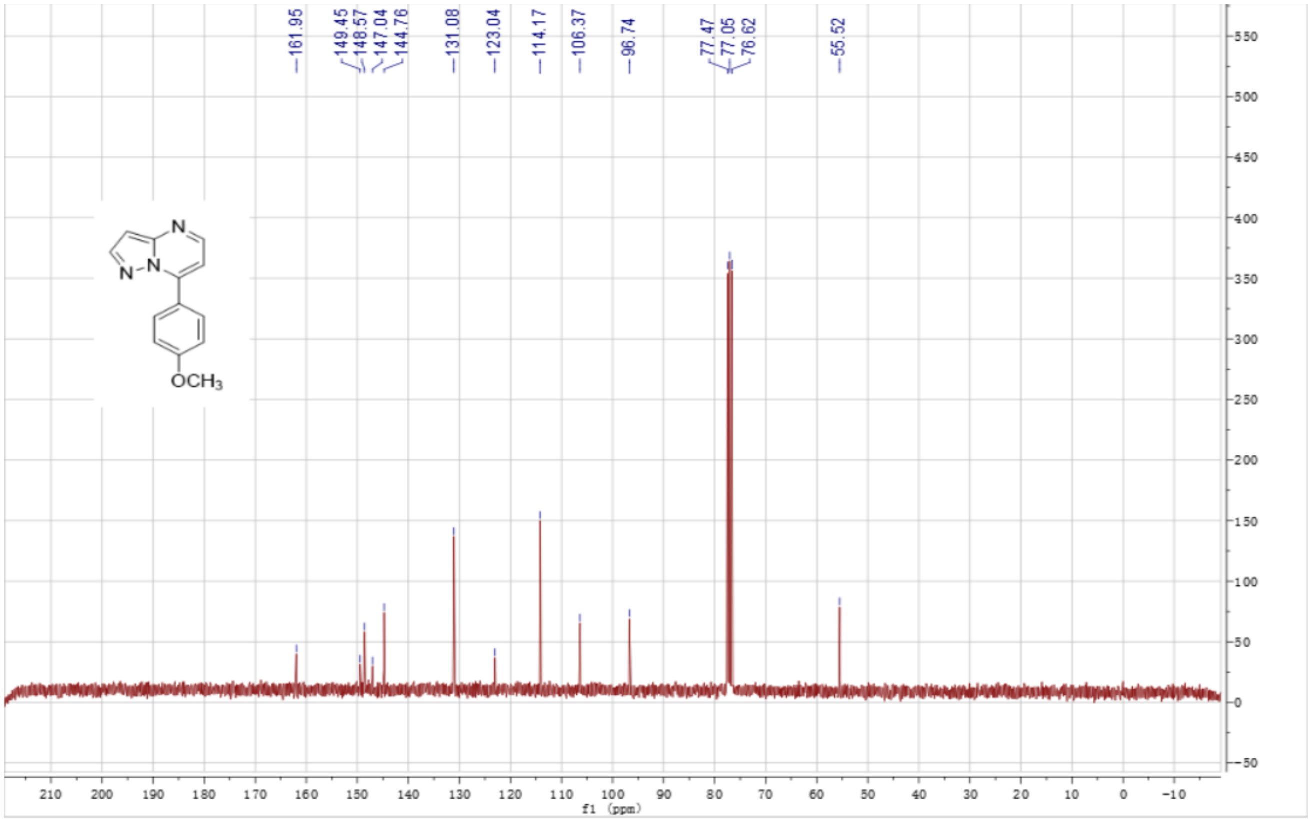


Figure 41 The C NMR spectrum of compound 4i

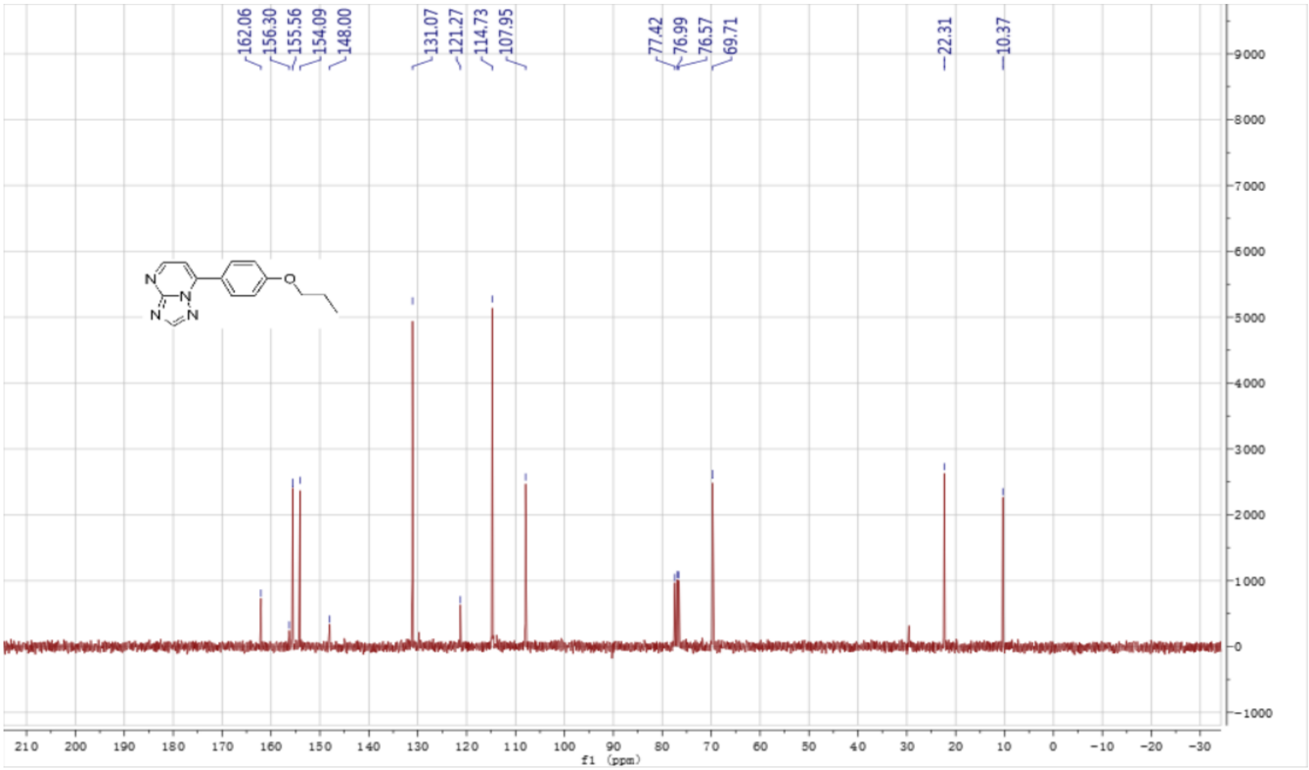


Figure 42 The C NMR spectrum of compound 6a

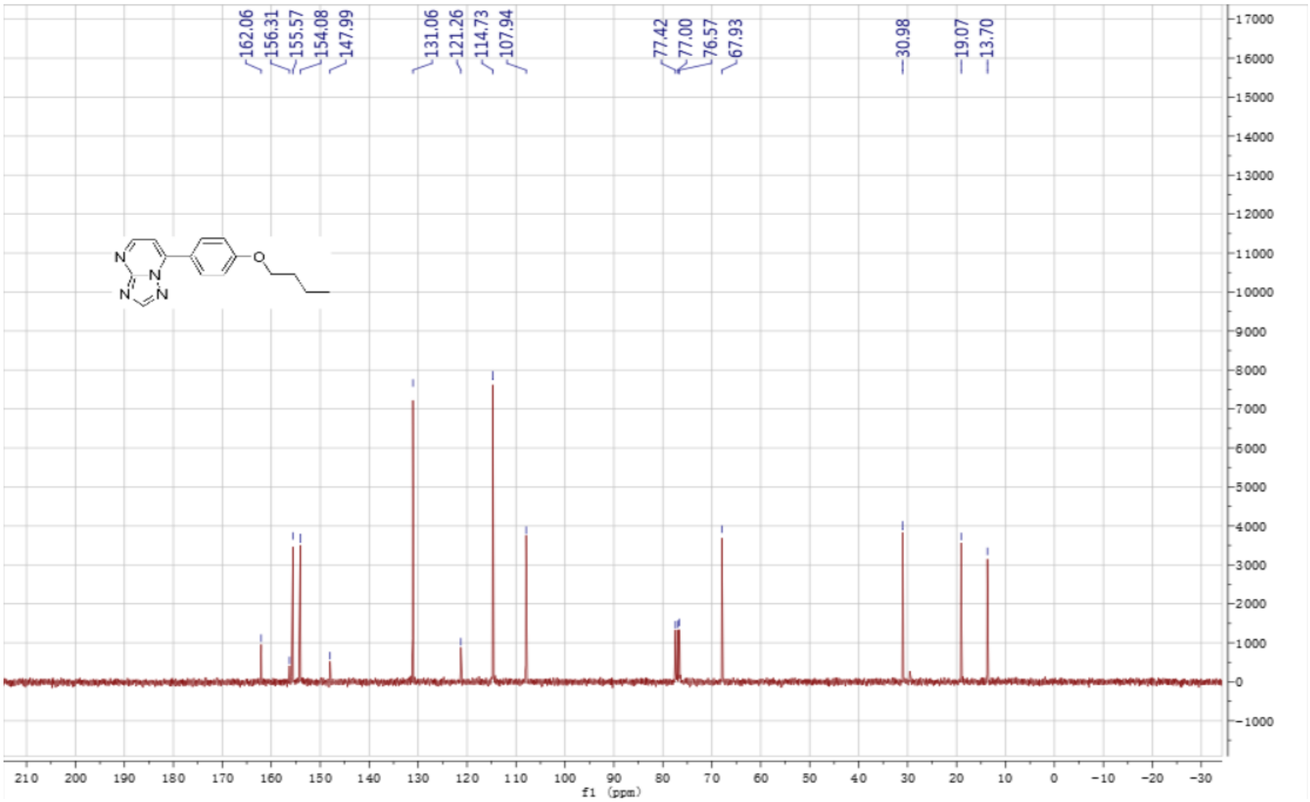


Figure 43 The C NMR spectrum of compound 6b

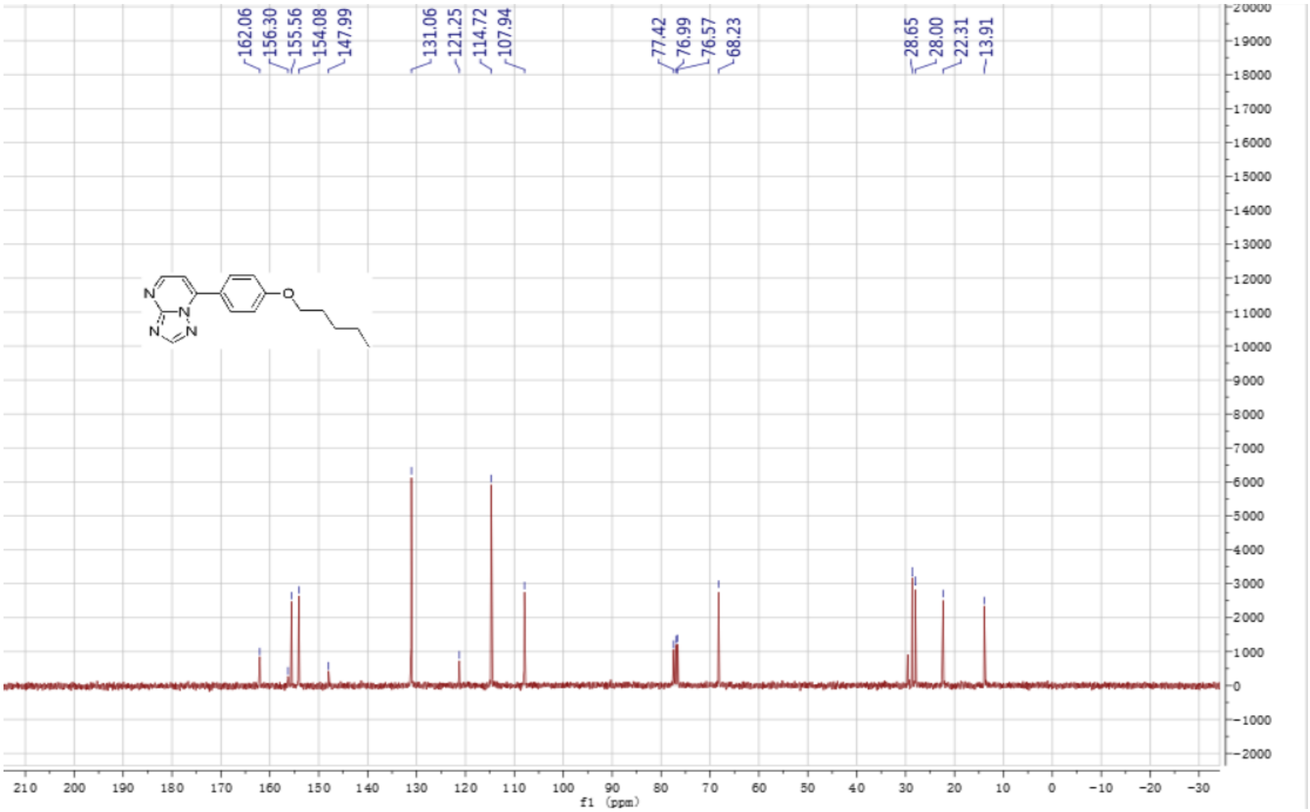


Figure 44 The C NMR spectrum of compound 6c

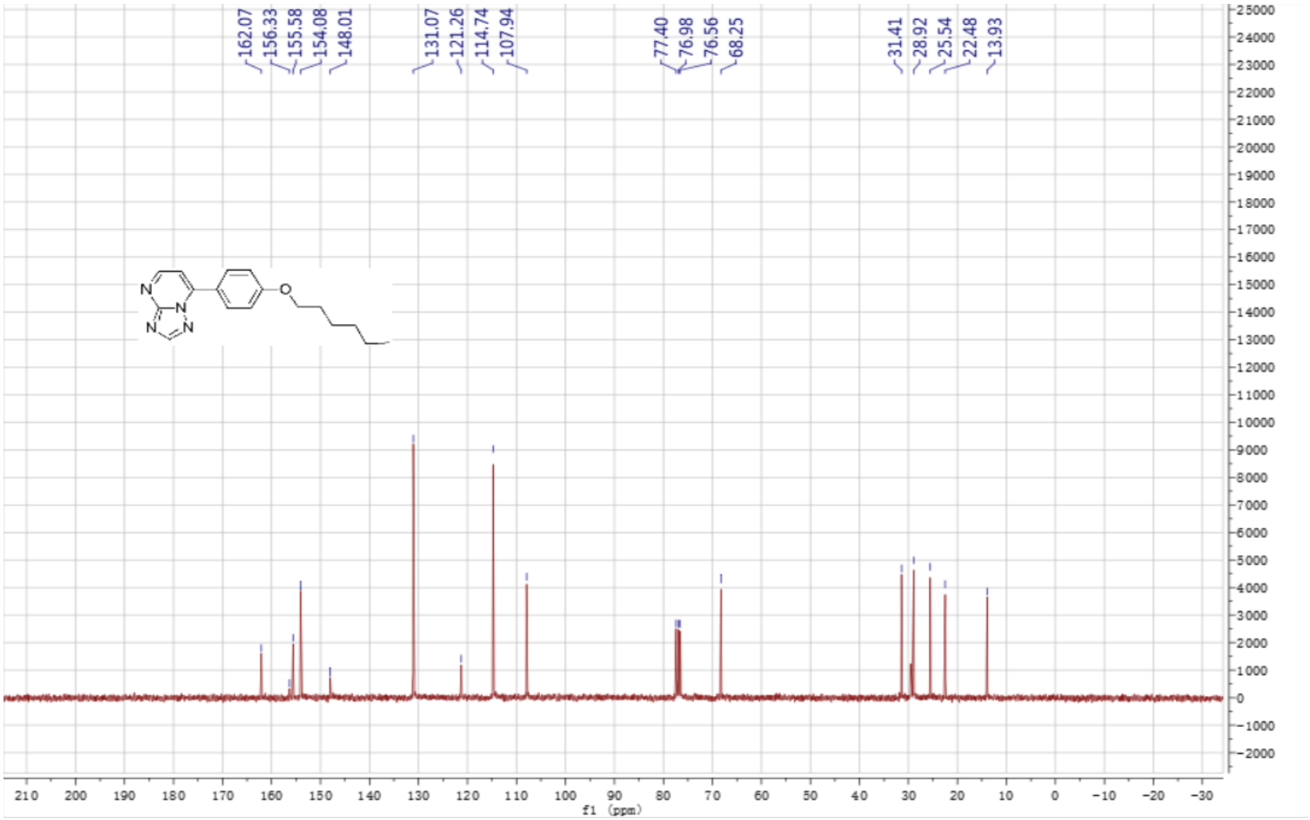


Figure 45 The C NMR spectrum of compound 6d

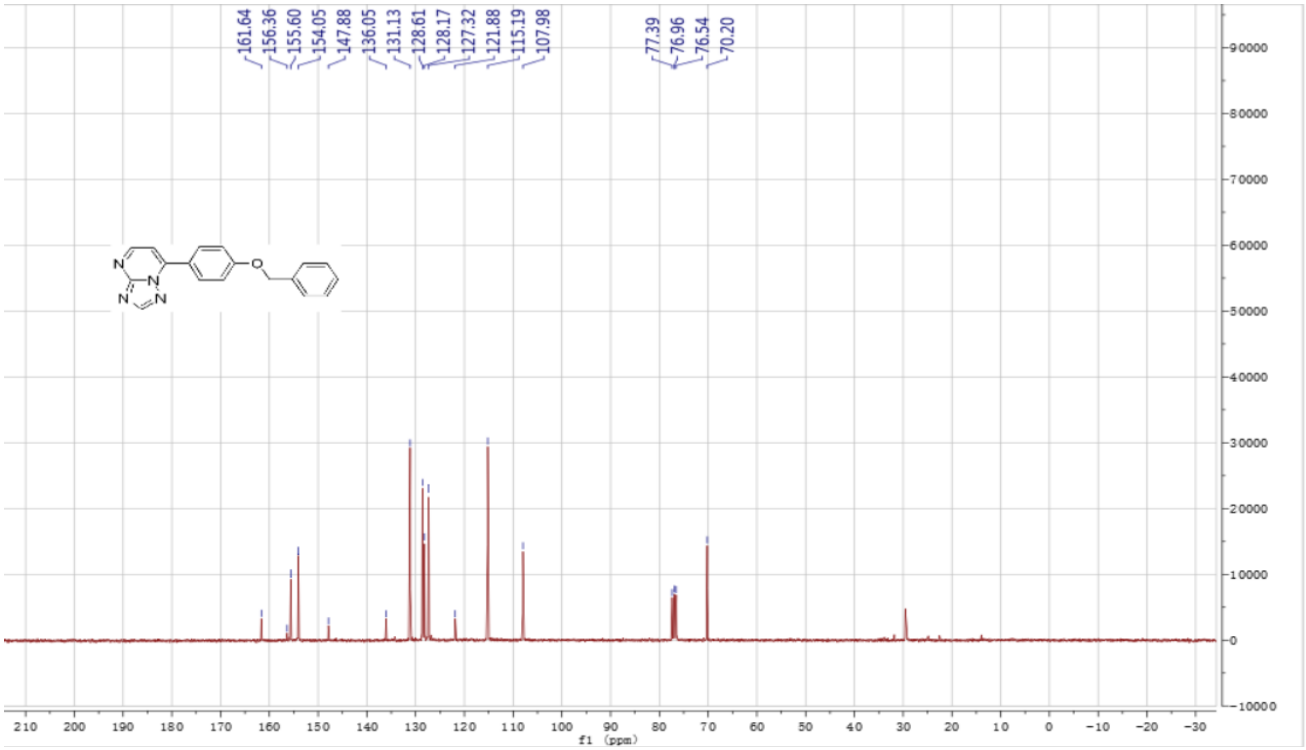


Figure 46 The C NMR spectrum of compound 6e

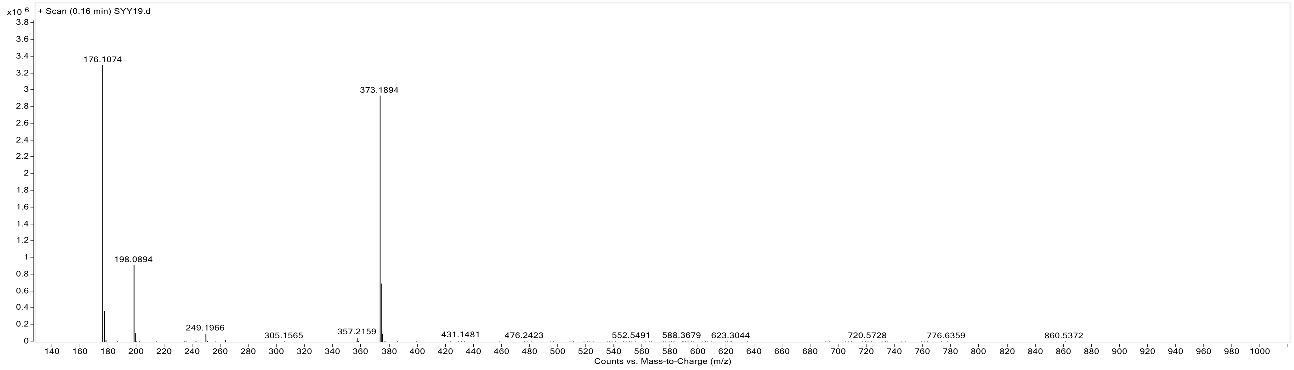


Figure 47 The HR-MS spectrum of compound 2a

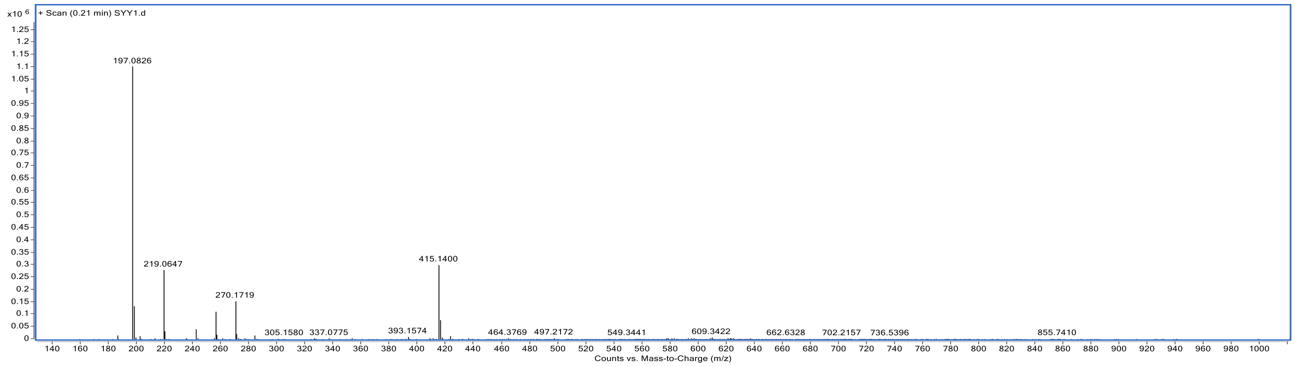


Figure 48 The HR-MS spectrum of compound 3a

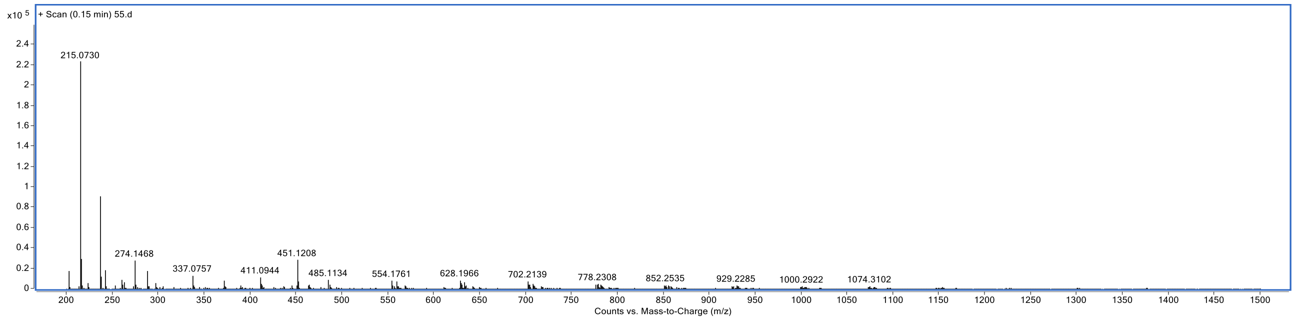


Figure 49 The HR-MS spectrum of compound 3b

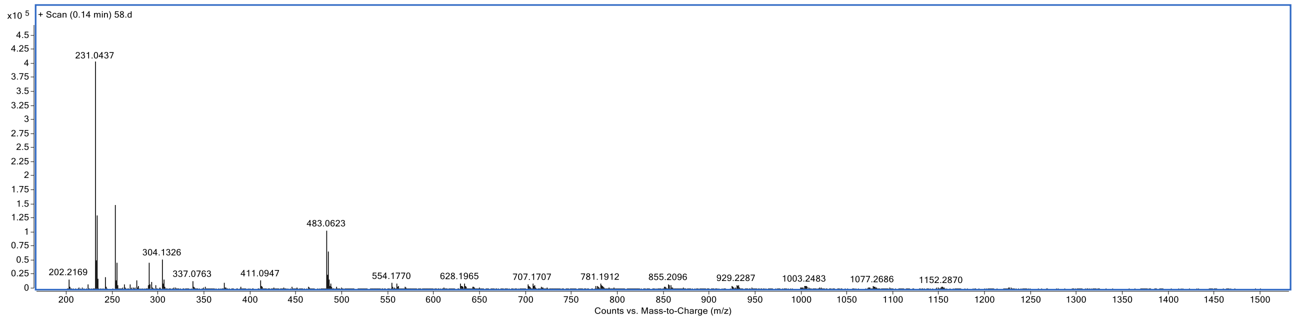


Figure 50 The HR-MS spectrum of compound 3c

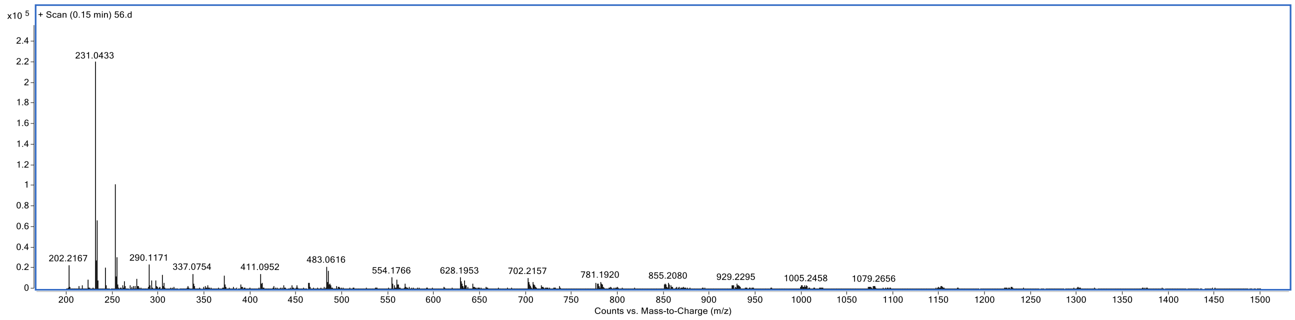


Figure 51 The HR-MS spectrum of compound 3d

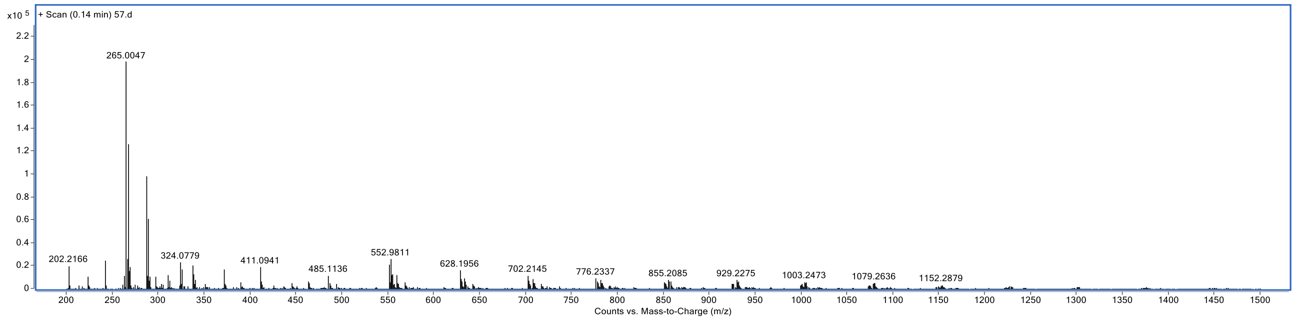


Figure 52 The HR-MS spectrum of compound 3e

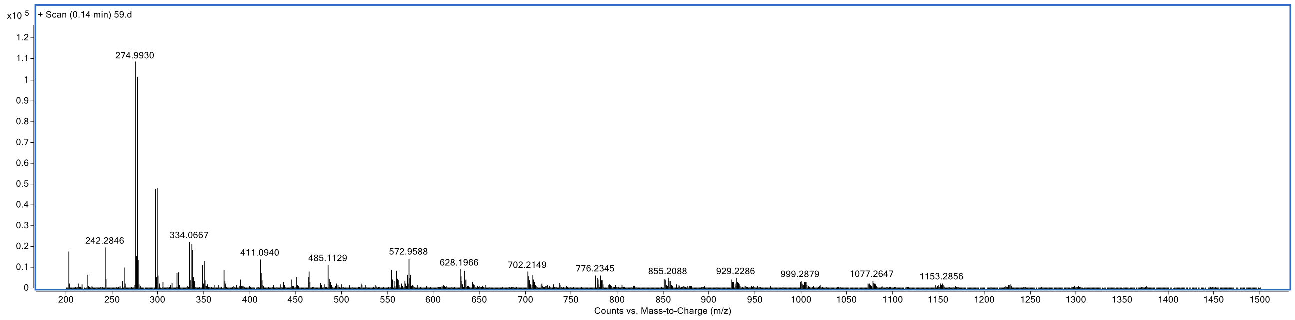


Figure 53 The HR-MS spectrum of compound 3f

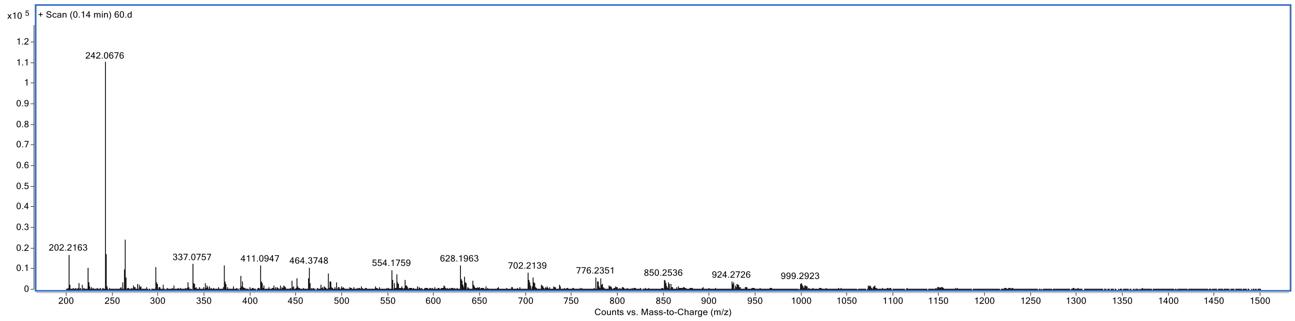


Figure 54 The HR-MS spectrum of compound 3g

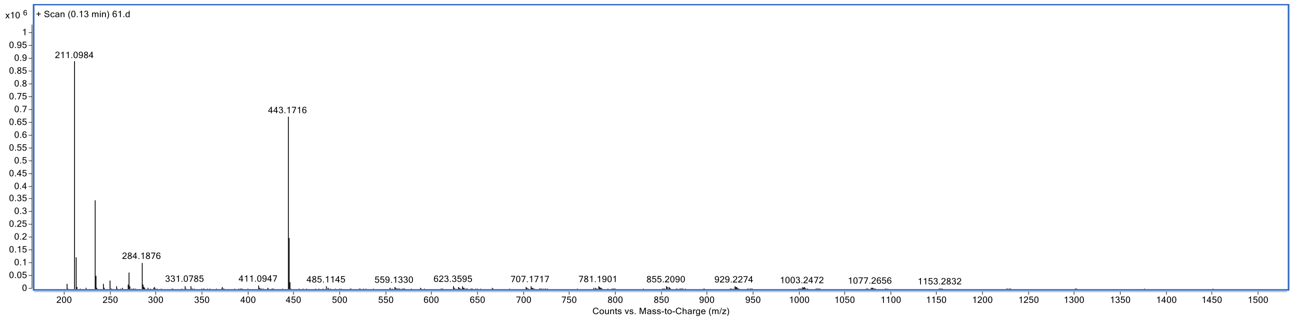


Figure 55 The HR-MS spectrum of compound 3h

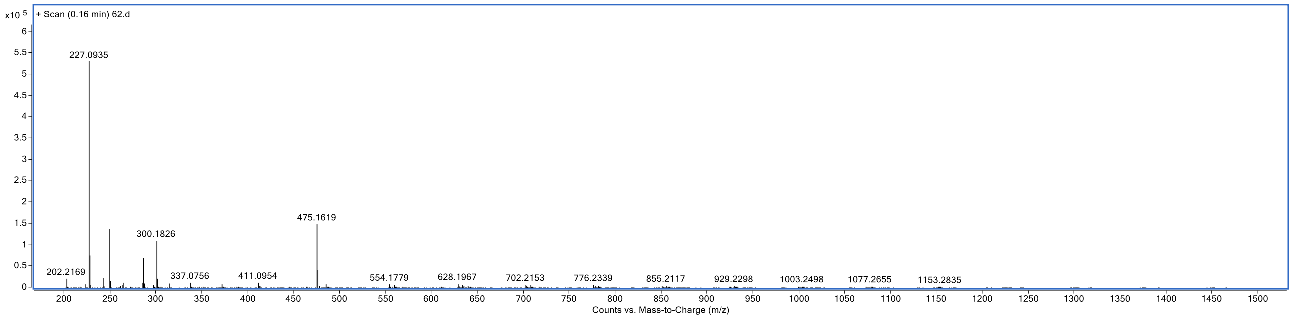


Figure 56 The HR-MS spectrum of compound 3i

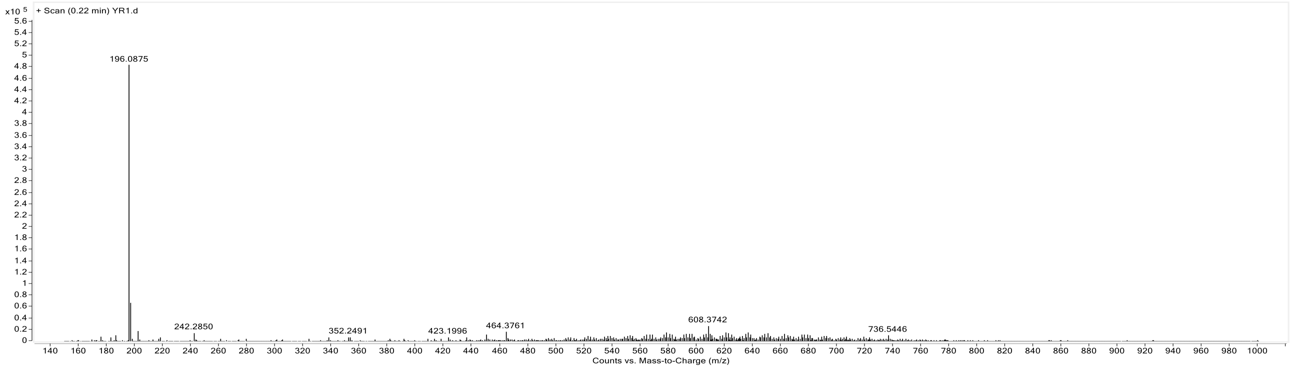


Figure 57 The HR-MS spectrum of compound 4a

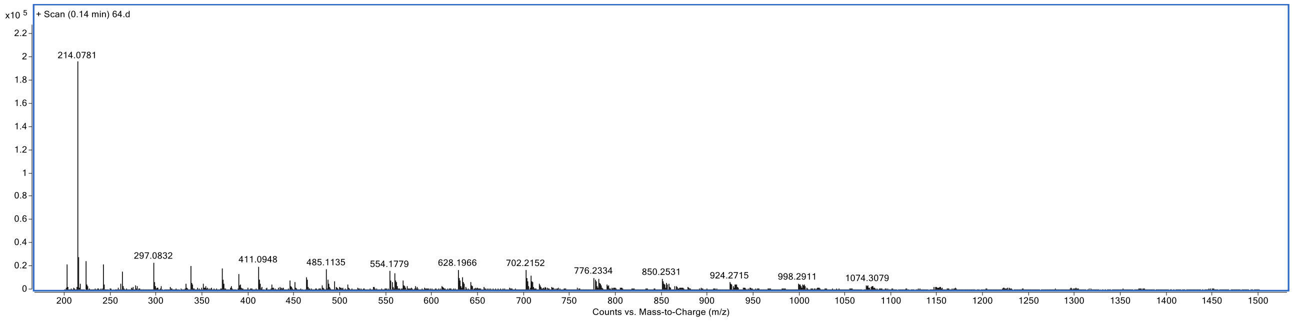


Figure 58 The HR-MS spectrum of compound 4b

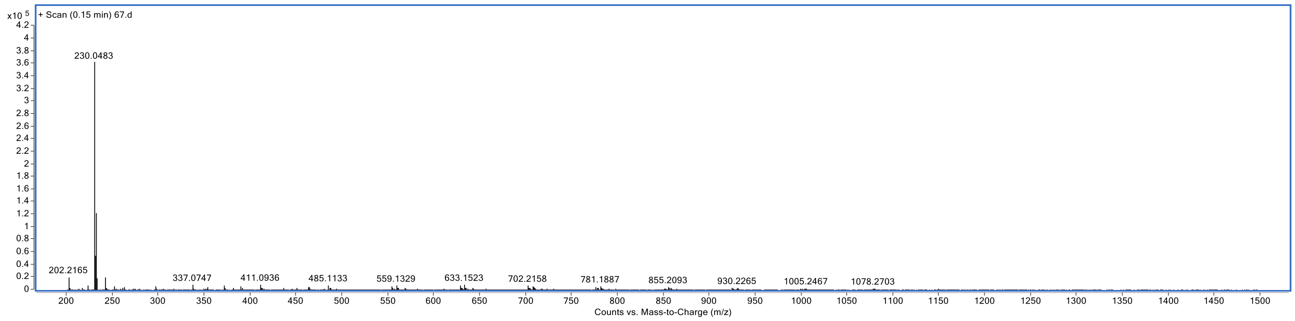


Figure 59 The HR-MS spectrum of compound 4c

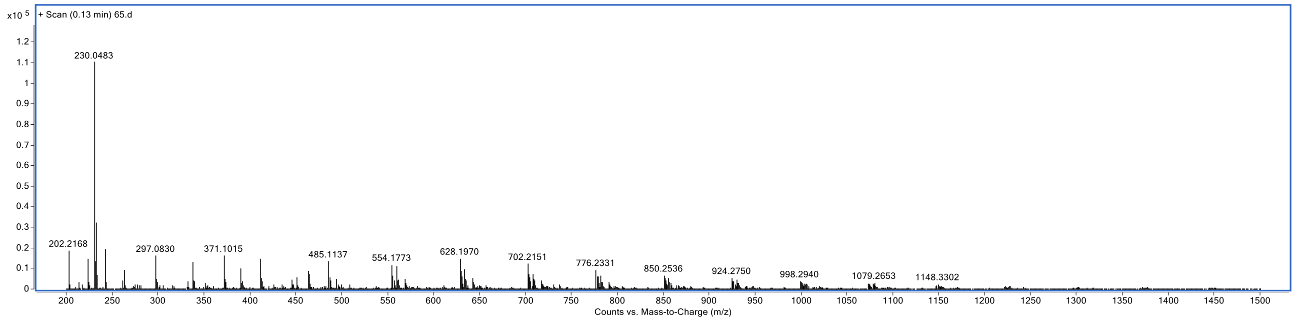


Figure 60 The HR-MS spectrum of compound 4d

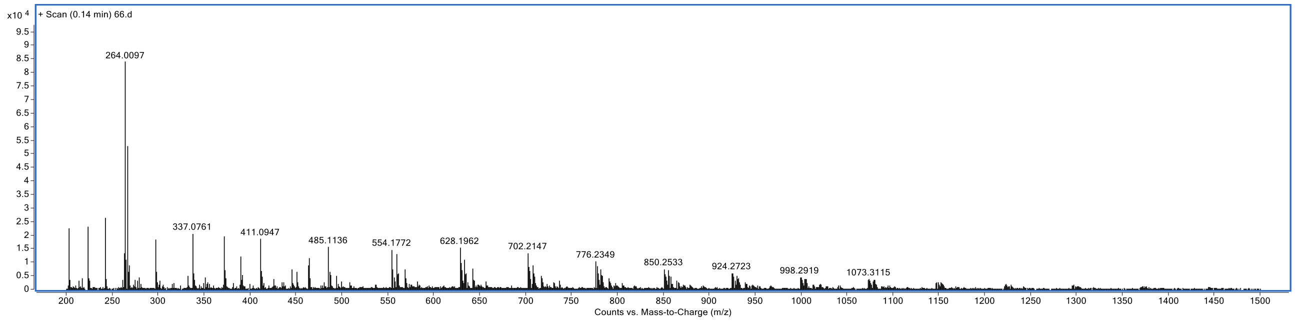


Figure 61 The HR-MS spectrum of compound 4e

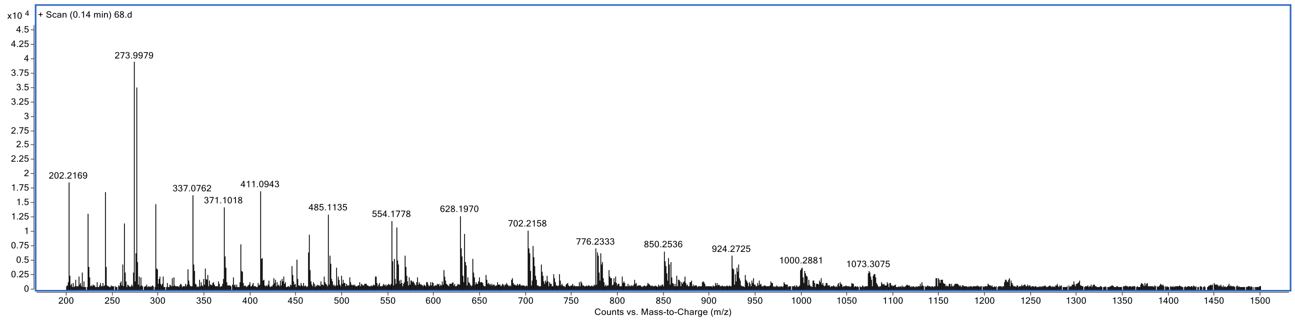


Figure 62 The HR-MS spectrum of compound 4f

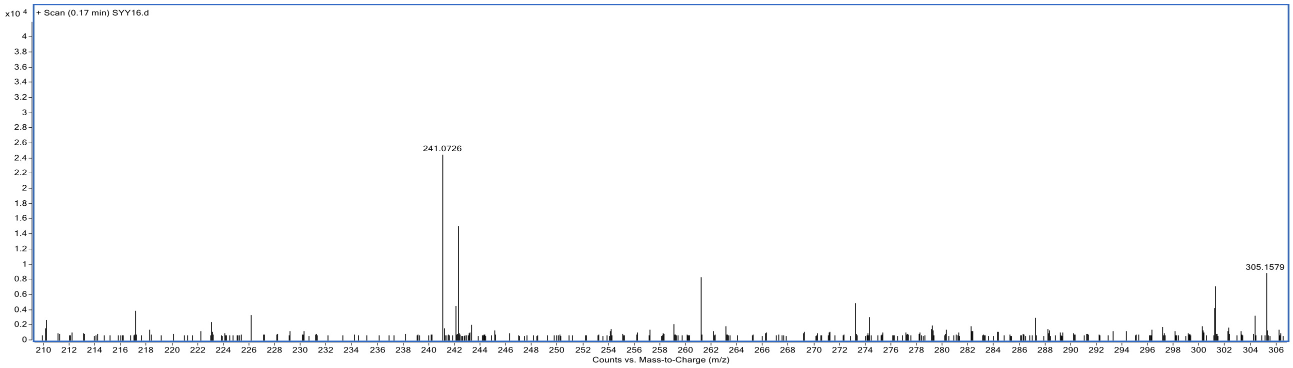


Figure 63 The HR-MS spectrum of compound 4g

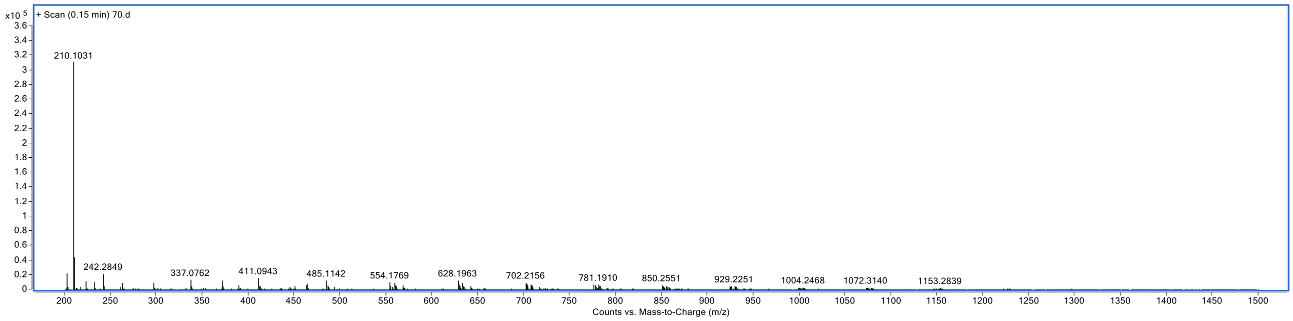


Figure 64 The HR-MS spectrum of compound 4h

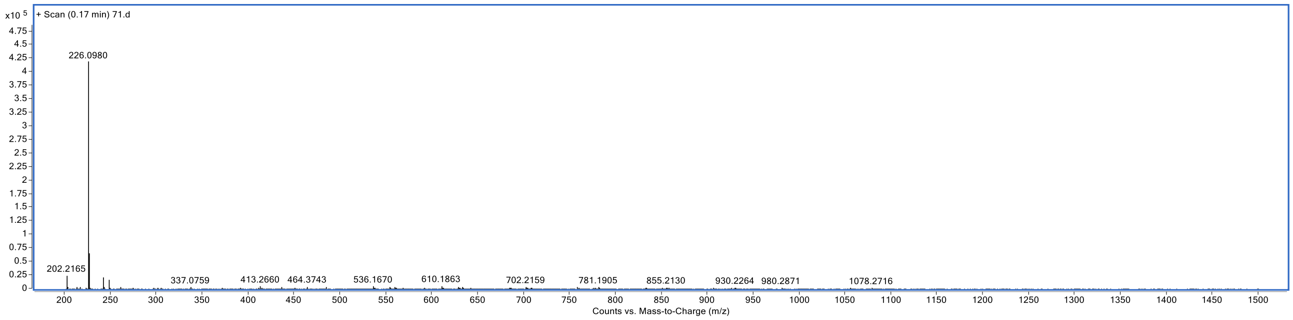


Figure 65 The HR-MS spectrum of compound 4i

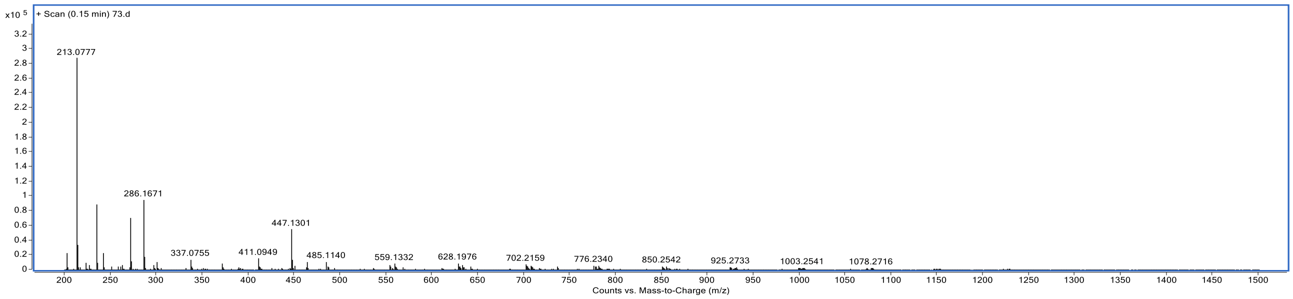


Figure 66 The HR-MS spectrum of compound 5

D:\Data\ZUCI\gc\171212\YSW-14\0_E14\1\1Ref

*Comment 1*
*Comment 2*

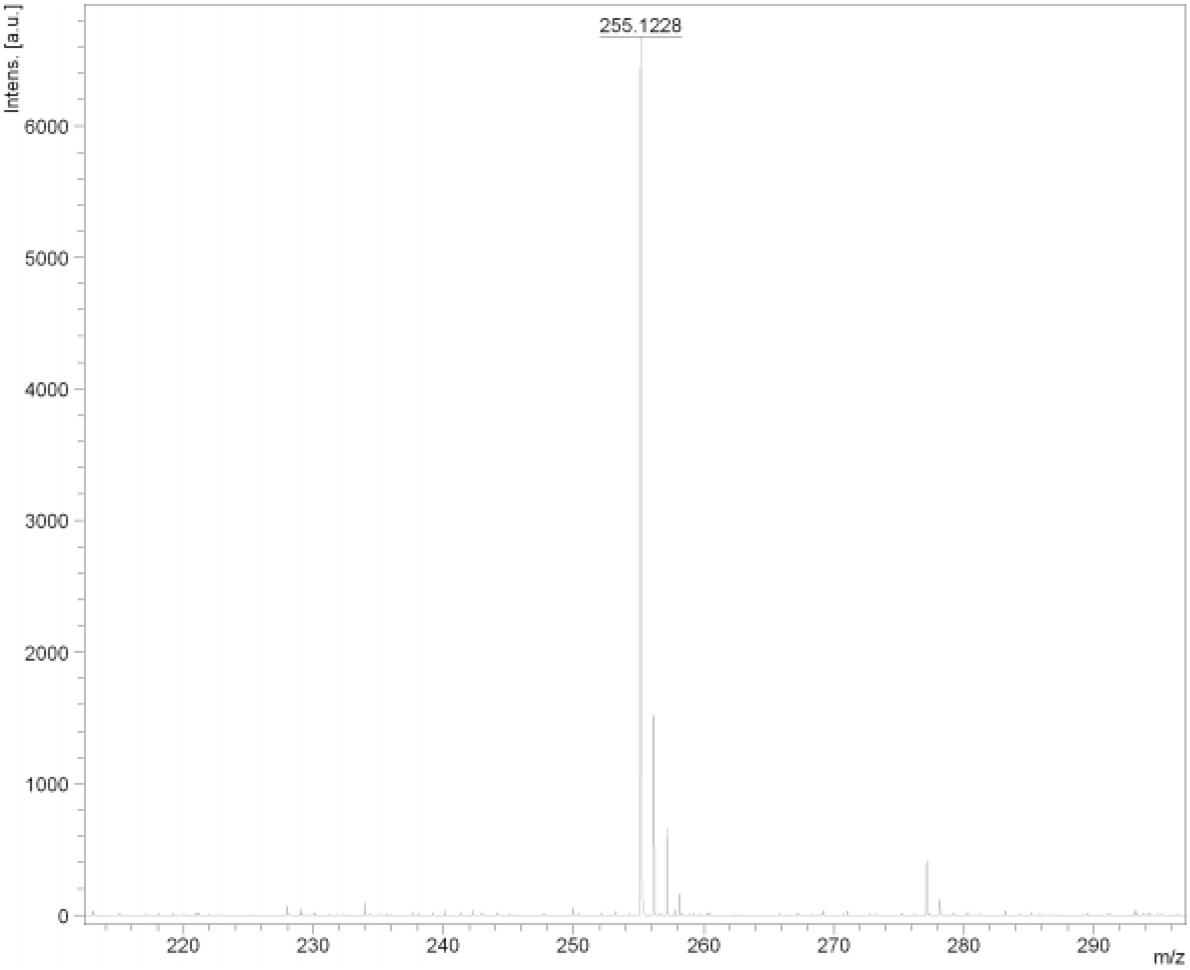


**Acquisition Parameter**

| | |
|---|---|
| *Date of acquisition* | 2017-12-12T16:36:55.297+08:00 |
| *Acquisition method name* | D:\Methods\flexControlMethods\gc-RP_100-1500_Da.par |
| *Aquisition operation mode* | Reflector |
| *Voltage polarity* | POS |
| *Number of shots* | 500 |
| *Name of spectrum used for calibration* | |
| *Calibration reference list used* | sample |

**Instrument Info**

Bruker Daltonics flexAnalysis printed: 12/13/2017 8:29:52 PM

Figure 67 The HR-MS spectrum of compound 6a

D:\Data\ZUCI\gc\171212\YSW-15\0_E15\1\1Ref

*Comment 1*

*Comment 2*

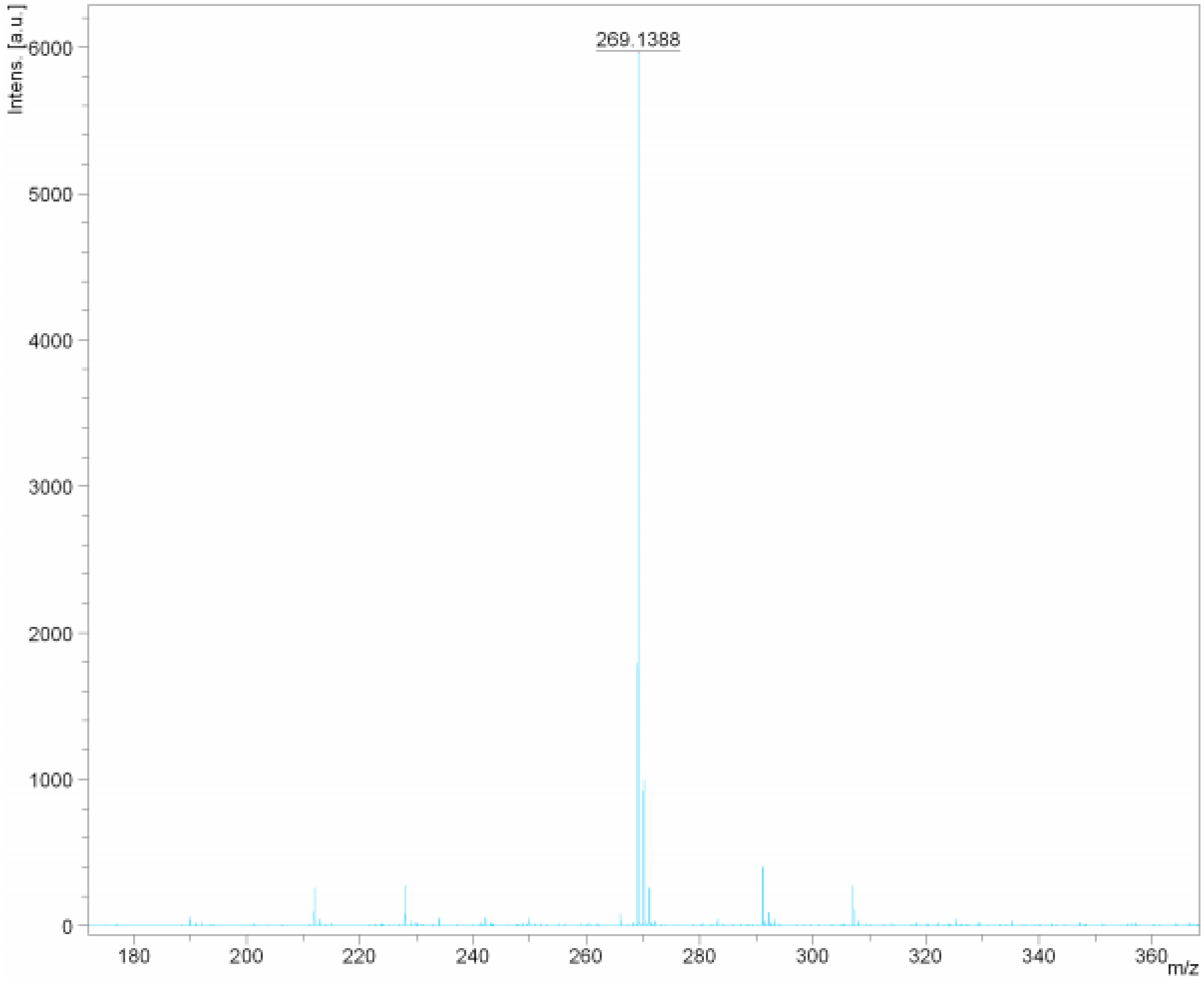


**Acquisition Parameter**

| | |
|---|---|
| *Date of acquisition* | 2017-12-12T16:37:05.500+08:00 |
| *Acquisition method name* | D:\Methods\flexControlMethods\gc-RP_100-1500_Da.par |
| *Aquisition operation mode* | Reflector |
| *Voltage polarity* | POS |
| *Number of shots* | 500 |
| *Name of spectrum used for calibration* | |
| *Calibration reference list used* | sample |

**Instrument Info**

Bruker Daltonics flexAnalysis printed: 12/12/2017 5:10:53 PM

Figure 68 The HR-MS spectrum of compound 6b

D:\Data\ZUCI\gc\171212\YSW-16\0_E16\1\1Ref

*Comment 1*

*Comment 2*

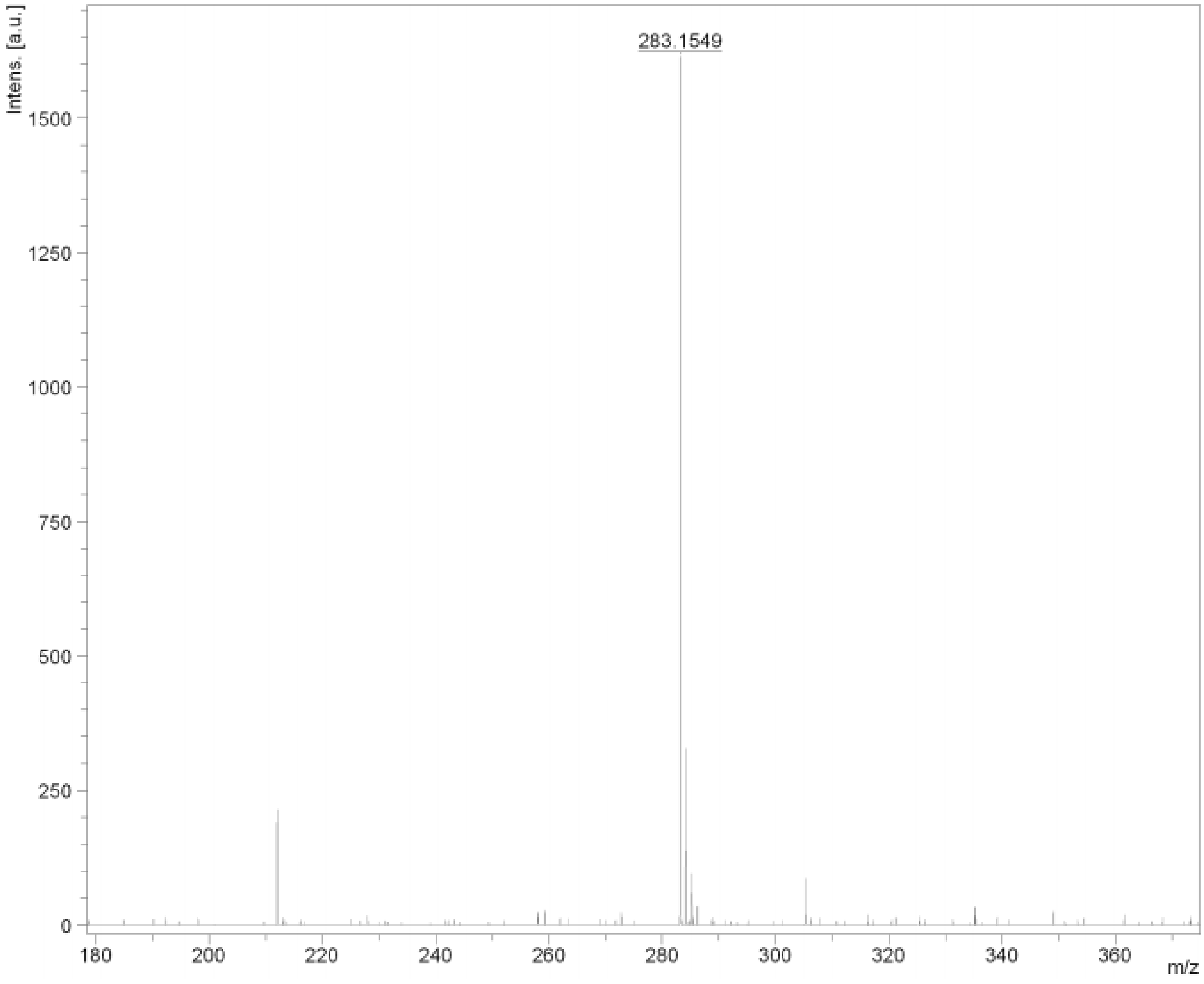


**Acquisition Parameter**

| | |
|---|---|
| *Date of acquisition* | 2017-12-12T16:37:17.454+08:00 |
| *Acquisition method name* | D:\Methods\flexControlMethods\gc-RP_100-1500_Da.par |
| *Aquisition operation mode* | Reflector |
| *Voltage polarity* | POS |
| *Number of shots* | 500 |
| *Name of spectrum used for calibration* | |
| *Calibration reference list used* | sample |

**Instrument Info**

Figure 69 The HR-MS spectrum of compound 6c

D:\Data\ZUCI\gc\171212\YSW-17\0_E17\1\1Ref

*Comment 1*

*Comment 2*

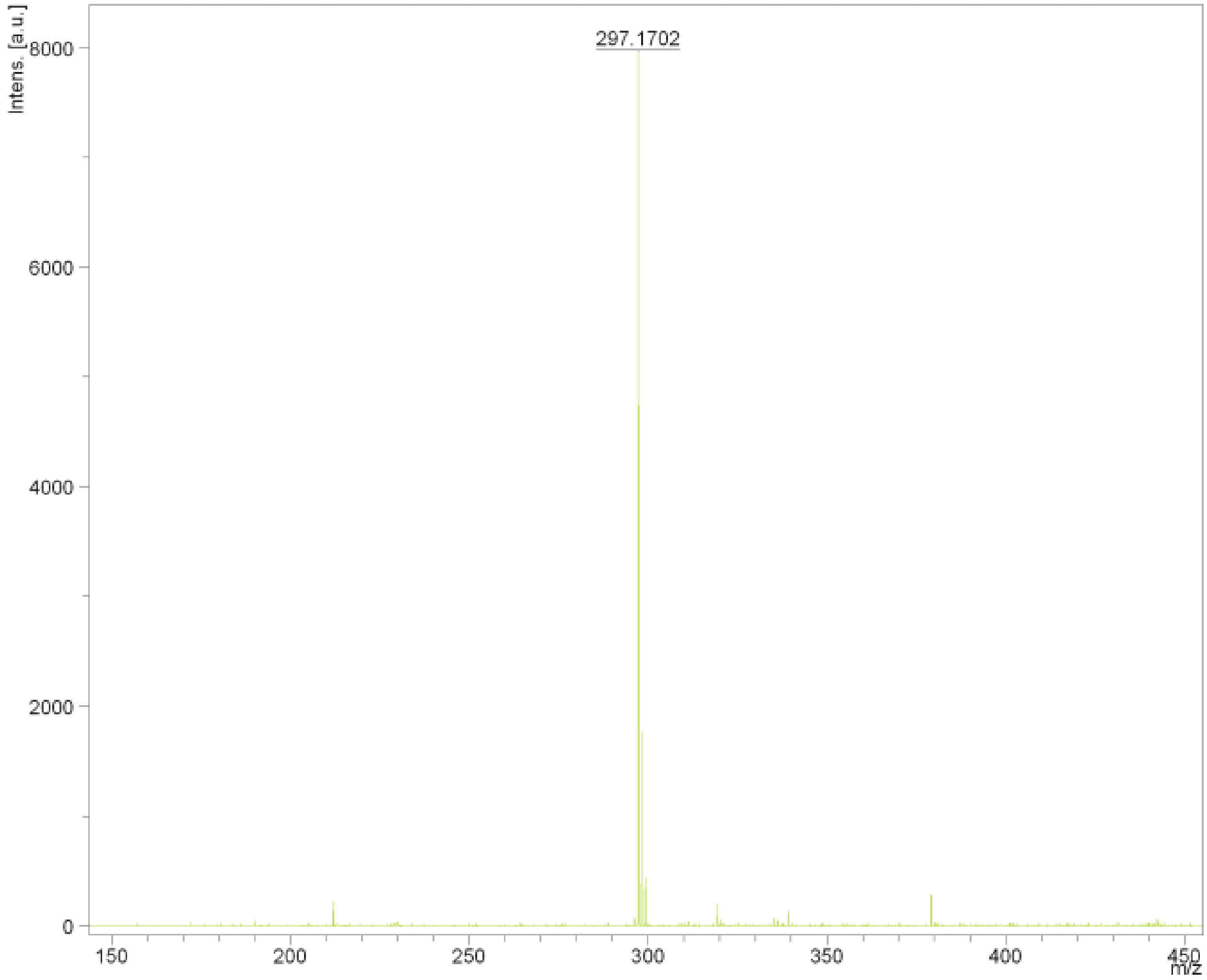


**Acquisition Parameter**

| | |
|---|---|
| *Date of acquisition* | 2017-12-12T16:37:37.813+08:00 |
| *Acquisition method name* | D:\Methods\flexControlMethods\gc-RP_100-1500_Da.par |
| *Aquisition operation mode* | Reflector |
| *Voltage polarity* | POS |
| *Number of shots* | 500 |
| *Name of spectrum used for calibration* | |
| *Calibration reference list used* | sample |

**Instrument Info**

Figure 70 The HR-MS spectrum of compound 6d

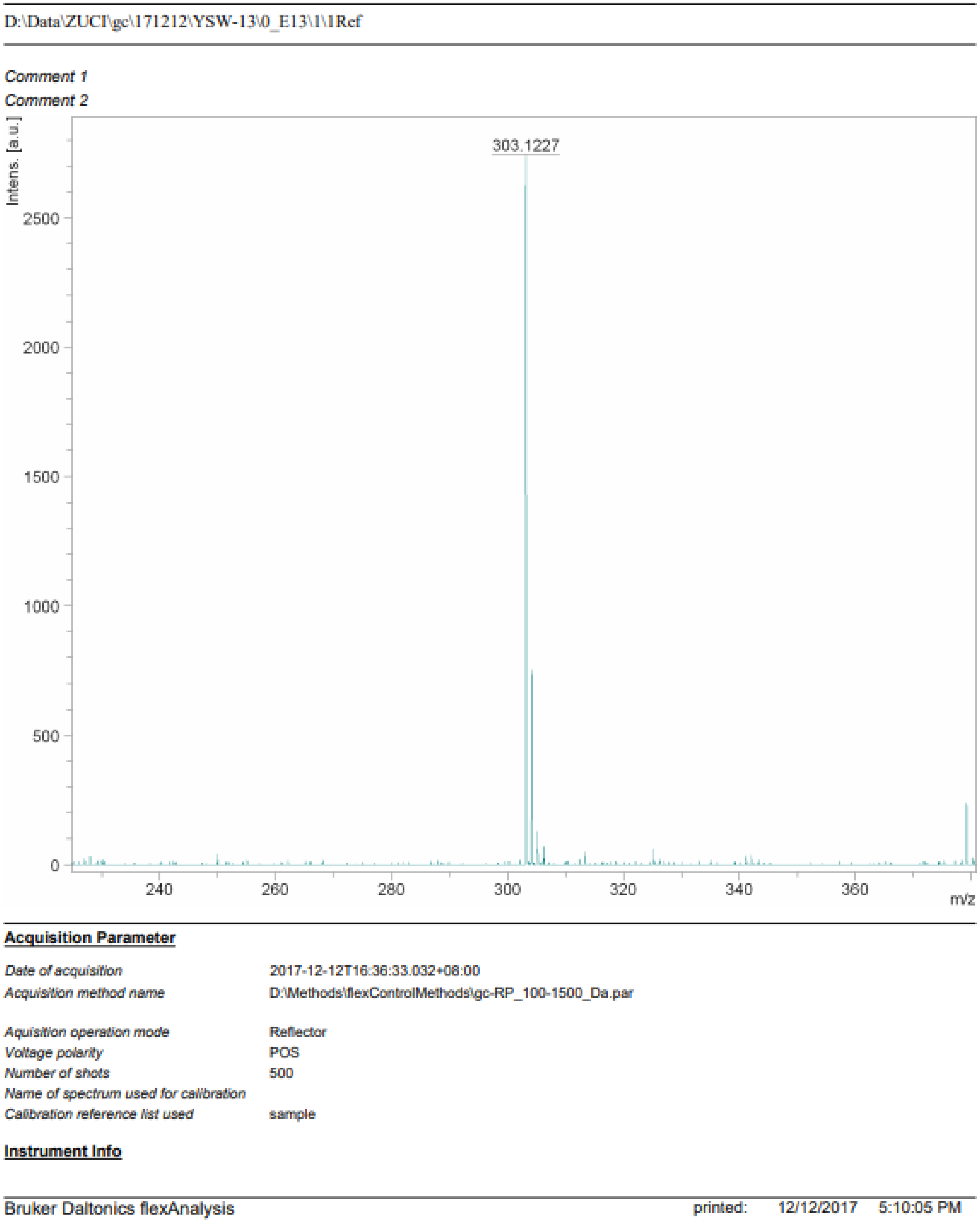


Figure 71 The HR-MS spectrum of compound 6e
